# Terminal Alkenes from Acrylic Acid Derivatives via Non‐Oxidative Enzymatic Decarboxylation by Ferulic Acid Decarboxylases

**DOI:** 10.1002/cctc.201800643

**Published:** 2018-07-17

**Authors:** Godwin A. Aleku, Christoph Prause, Ruth T. Bradshaw‐Allen, Katharina Plasch, Silvia M. Glueck, Samuel S. Bailey, Karl A. P. Payne, David A. Parker, Kurt Faber, David Leys

**Affiliations:** ^1^ Manchester Institute of Biotechnology School of Chemistry University of Manchester 131 Princess Street Manchester M1 7DN United Kingdom; ^2^ Austrian Centre of Industrial Biotechnology (ACIB) 8010 Graz Austria) c/o; ^3^ Department of Chemistry University of Graz Heinrichstrasse 28 8010 Graz Austria).; ^4^ Innovation/Biodomain Shell International Exploration and Production Inc. Westhollow Technology Center Houston USA

**Keywords:** Biocatalysis, Ferulic acid decarboxylase, Prenylated flavin, Decarboxylation, Terminal alkenes

## Abstract

Fungal ferulic acid decarboxylases (FDCs) belong to the UbiD‐family of enzymes and catalyse the reversible (de)carboxylation of cinnamic acid derivatives through the use of a prenylated flavin cofactor. The latter is synthesised by the flavin prenyltransferase UbiX. Herein, we demonstrate the applicability of FDC/UbiX expressing cells for both isolated enzyme and whole‐cell biocatalysis. FDCs exhibit high activity with total turnover numbers (TTN) of up to 55000 and turnover frequency (TOF) of up to 370 min^−1^. Co‐solvent compatibility studies revealed FDC's tolerance to some organic solvents up 20 % v/v. Using the in‐vitro (de)carboxylase activity of holo‐FDC as well as whole‐cell biocatalysts, we performed a substrate profiling study of three FDCs, providing insights into structural determinants of activity. FDCs display broad substrate tolerance towards a wide range of acrylic acid derivatives bearing (hetero)cyclic or olefinic substituents at C3 affording conversions of up to >99 %. The synthetic utility of FDCs was demonstrated by a preparative‐scale decarboxylation.

## Introduction

The production of organic building blocks from renewable carbon sources is a current trend in synthetic organic chemistry.^1^, ^2^, ^3^, ^4^ The major primary intermediates of traditional industrial‐scale synthesis are light alkenes such as ethylene, propylene and butadiene which are produced from crude oil *via* steam‐cracking, which has been described as the single most energy‐demanding process in the petrochemical industry.^5^,^6^

In view of the fact that biocatalytic transformations are operational under mild and environmentally‐friendly conditions and proceed with high chemo‐, regio‐ and stereoselectivity,^7^ there is an increasing interest in expanding the scope and efficiency of enzymatic reactions.^8^, ^9^, ^10^, ^11^, ^12^, ^13^ Biological routes towards alkenes are rare and have been investigated only recently.^14^, ^15^, ^16^, ^17^, ^18^, ^19^, ^20^ For instance, oxidative decarboxylation of (saturated) fatty acids by the P450 mono‐oxygenase OleT^[21–23^] and the non‐heme oxygenase UndA^24^ yields terminal alkenes on a small scale.^25^ In order to avoid the requirement for sophisticated and sensitive electron‐transfer proteins, redox‐neutral decarboxylation of *p*‐hydroxycinnamic acids (′phenolic acids′) derived from the breakdown of lignin catalysed by phenolic acid decarboxylases was investigated.^7^ The latter enzymes act *via* simple acid‐base catalysis,^26^ which requires the presence of a phenolic ′activating′ group in the substrate, which severely limits their applicability. Furthermore, the electron‐rich *p*‐hydroxystyrenes thus obtained are not very stable and are prone to (spontaneous) oxidation and polymerisation.

Ferulic acid decarboxylases (FDCs) acting on ′non‐phenolic′ cinnamic acids are an intriguing new class of decarboxylases.^27^, ^28^, ^29^ They are distinct members of the UbiD family of decarboxylases and catalyse the non‐oxidative decarboxylation of acrylic acid derivatives such as cinnamic, ferulic and sorbic acid yielding the corresponding terminal alkenes.^30^,^31^ Recent structural and mechanistic studies revealed that these enzymes utilise a prenylated derivative of flavin (prFMN), a cofactor synthesised by UbiX.^32^ FDC‐catalysed decarboxylation of cinnamic acid derivatives mediated by prFMN is proposed to proceed *via* a 1,3‐dipolar cycloaddition,^27^,^28^ in which prFMN acts as 1,3‐dipolar diene owing to its azomethine ylide character.^28^,^33^, ^34^, ^35^ While this type of transformation – commonly referred to as ′Huisgen‐reaction′^36^,^37^ – is widely utilised in heterocyclic synthesis, enzymatic equivalents to this reaction are rare.^38^, ^39^, ^40^, ^41^

**Scheme 1:**
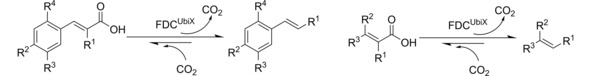
Enzymatic decarboxylation of α,β‐unsaturated carboxylic acids.

Herein, we report on the broad substrate scope and high activity of three FDCs (Scheme 1). Crucial reaction parameters such as co‐solvent compatibility, temperature‐ and pH‐optima of these enzymes were investigated. Furthermore, we also performed a preparative‐scale biotransformation and tested *Sc*FDC in the (reverse) carboxylation of terminal alkenes utilising KHCO_3_ or pressurized CO_2_ as C_1_ source.^4^,^42^, ^43^, ^44^, ^45^

## Results and Discussion

### Optimisation of Biotransformation Conditions

In order to assess the biocatalytic potential of FDCs, three previously described representatives^28^ from *Aspergillus niger (An*FDC*), Saccharomyces cerevisae (Sc*FDC*) and Candida dubliniensis* (*Cd*FDC) were each co‐expressed with the native *E. coli* UbiX in *E. coli* to produce the holo‐enzymes *An*FDC^UbiX^, *Sc*FDC^UbiX^ and *Cd*FDC^UbiX^. In this system, the FDCs were fused with a polyhistidine tag, whereas UbiX was co‐expressed untagged to enable *in vivo* production of prFMN, allowing for the purification of the prFMN‐bound FDC to homogeneity by Ni affinity chromatography.

Using purified *An*FDC^UbiX^ as the catalyst, biotransformation conditions were optimised for the decarboxylation of 20 mM **1 a** as a model reaction. The enzyme displayed a broad pH window (pH 6.0–9.0) with highest conversions of >99 % achieved at pH 7.5 (phosphate buffer) and pH 8.0 (Tris‐HCl buffer) (Supporting information Section S1.1). *An*FDC^UbiX^ showed high activity between 20 and 45 °C with highest rates obtained at 37–42 °C, however protein precipitation was observed upon incubation at ≥37 °C for 1 h. Hence, subsequent reactions were performed at 30 °C. Under the optimised conditions, biotransformations were performed with i) freshly purified enzyme preparations (snap‐frozen or lyophilised), ii) *E. coli* whole cells containing *An*FDC either as fresh resting whole cells or in lyophilised form, and iii) using fresh cell‐free extract (snap‐frozen or lyophilised). In all cases, conversions of >80 % were achieved highlighting the suitability of FDCs in isolated form or as whole cell biocatalyst. Similarly, lyophilised whole‐cell *Sc*FDC showed a broad temperature optimum between 30 °C and 45 °C, with a sharp drop beyond this value, while the pH‐profile peaked at 6.0 (Supporting information Section S1.3). Monitoring *Sc*FDC‐catalysed decarboxylation of (aromatic) ferulic and (non‐aromatic) sorbic acid over time revealed a typical hyperbolic decline of the substrate concentration, where ∼90 % conversion was reached within ∼8 h, and the reaction was complete after ∼16 h (Supporting Information, Figure S5). Control reactions featuring all reaction conditions but containing *E. coli* whole cells harbouring an empty pET vector revealed no conversion of **1 a**.

### Substrate Tolerance of FDCs

**Scheme 2:**
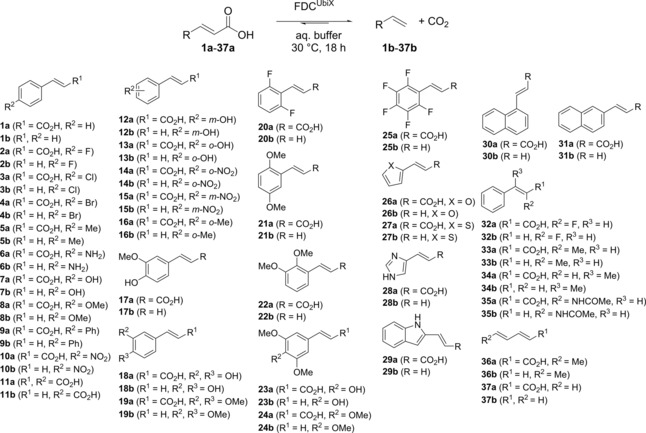
Substrates (**1 a**–**37 a**) decarboxylated by FDCs and their corresponding products (**1 b**–**37 b**).

**Figure 1:**
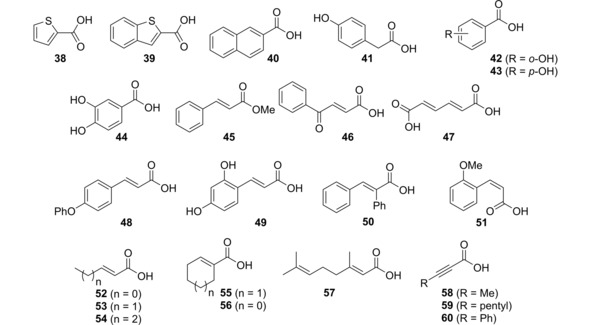
Substrates rejected by FDC (conversion <1 %), for standard conditions see Table 1.

To highlight the synthetic utility of FDCs, the substrate scope of *An*FDC, *Sc*FDC and *Cd*FDC was investigated. An array of 60 different α,β‐unsaturated carboxylic acids were tested in the decarboxylation direction encompassing substituted cinnamic acids and heterocyclic analogs thereof, as well as non‐aromatic acrylic acid derivatives and α,β‐acetylenic substrates (Scheme 2 & Figure 1). Initially, isolated enzymes were used for the substrate profiling study (Table 1). In addition, *Sc*FDC was also applied as lyophilised whole cell preparation (overexpressed in *E. coli*) to evaluate its applicability on preparative‐scale for potential industrial use. Overall, a broad set of substrates covering different structural motifs and electronical properties were employed (Table 1).

**Table 1:** **Table 1** FDC‐catalysed decarboxylation of acrylic acid derivatives (1 a‐37 a).

Entry	Substrates	Conversion [%]			
		*An*FDC^UbiX^	*Sc*FDC^UbiX^		*Cd*FDC^UbiX^
			purified *Sc*FDC^UbiX^	*E. coli* whole cells^[a]^	
1	**1 a**	>99	>99	>99	96
2	**2 a**	>99	88	>99	84
3	**3 a**	n.d.	n.d.	>99	n.d.
4	**4 a**	>99	97	n.d.	98
5	**5 a**	>99	>99	>99	98
6	**6 a**	78	75	>99	61
7	**7 a**	68	73	86	80
8	**8 a**	n.d.	n.d.	>99	n.d.
9	**9 a**	40	<5	n.d.	<5
10	**10 a**	50	25	n.d.	18
11	**11 a**	5^[b]^	5^[b]]^	n.d.	8^[b]^
12	**12 a**	6	26	n.d.	38
13	**13 a**	8	5	n.d.	4
14	**14 a**	10	5	n.d.	4
15	**15 a**	61	17	n.d.	16
16	**16 a**	>99	>99	>99	97
17	**17 a**	35	47	>99	47
18	**18 a**	n.d.	n.d.	33	n.d.
19	**19 a**	n.d.	n.d.	>99	n.d.
20	**20 a**	n.d.	n.d.	82	n.d.
21	**21 a**	n.d.	n.d.	36	n.d.
22	**22 a**	n.d.	n.d.	31	n.d.
23	**23 a**	15	3	9	6
24	**24 a**	5	8	n.d.	6
25	**25 a**	91	94	n.d.	81
26	**26 a**	>99	>99	>99	>99
27	**27 a**	92	87	>99	68
28	**28 a**	5	n.d.	<1	n.d.
29	**29 a**	42	14	n.d.	31
30	**30 a**	>99	>99	n.d.	>99
31	**31 a**	>99	>99	>99	>99
32	**32 a**	97	58	n.d.	47
33	**33 a**	22	60	n.d.	20
34	**34 a**	85	77	n.d.	50
35	**35 a**	6	<3	n.d.	<3
36	**36 a**	95	80	>99	88
37	**37 a**	99	90	>99	87

Reaction conditions using purified enzymes: substrate (5 mM), purified enzyme (0.2 mg mL^−1^), NaP_*i*_ buffer (100 mM, pH 7.5), 30 °C, 180 rpm, 18 h; conversion values were determined by GC‐MS or HPLC analysis; [a] reaction conditions with *E. coli* whole cells: substrate (10 mM), *Sc*FDC^UbiX^ *E. coli* whole cells (30 mg mL^−1^), NaP_i_ buffer (100 mM, pH 6.0), 30 °C, 120 rpm, 18 h, 5 % v/v DMSO (20 % v/v DMSO for **31 a** and **48**); n.d.=not determined; [b] decarboxylation occurred at the acrylic acid moiety furnishing 4‐vinyl benzoic acid (**11 b**) as sole product.

First, a range of cinnamic acid derivatives with various substituents at the **p**‐position of the aromatic moiety (**1 a**–**11 a**) were examined. Substrates bearing weakly electron‐withdrawing groups such as *p*‐halogens (**2 a**–**4 a**) and weakly e^−^‐donating groups such as *p‐*methyl (**5 a**) were well tolerated by the enzymes affording >84 % conversion (Table 1, entries 2–5). Strong e^−^‐donating groups such as *p*‐NH_2_ **6 a**, *p*‐OH **7 a** and *p*‐OMe **8 a** were perfectly accepted by whole cells (c=86‐99 %, entries 6–8) while a drop in conversion was observed with purified enzymes as catalyst (c=61–80 %). A strong e^−^‐withdrawing *p*‐NO_2_ group (**10 a**) led to diminished conversions (c=18–50 %, entry 10) using purified enzymes. Steric restriction seems to appear with a larger *p*‐Ph group (**9 a**) which was only reasonably accepted by FDC from *A. niger* (c=40 %, entry 9). Complete loss of activity was observed with an even larger substituent (*p*‐OPh, **48**, Figure 1). Substrate **11 a** which carries two carboxyl groups was regioselectively decarboxylated yielding 4‐vinyl benzoic acid (**11 b**) as sole product, albeit in low conversions of up 8 % (entry 11). Remarkably enough, in contrast to phenolic acid decarboxylases (PADs), the confining requirement for an activating *p*‐hydroxy group proved to be dispensable which is in line with the proposed 1,3‐dipolar cycloaddition mechanism of FDCs.

The influence of the substitution pattern at the aromatic ring on the enzyme's performance has been further evaluated applying mono‐ (*o*‐ or *m*‐, for *p*‐ see above), di‐, tri‐ and penta‐functionalised cinnamic acid derivatives. A NO_2_‐substituent in *m*‐position was similarly tolerated as the *p*‐analogue (**10 a** versus **15 a**, entries 10, 15) whereas a strong e^−^‐donating group (such as OH) in *m*‐position led to reduced reaction rates compared to the *p*‐pendant (**7 a** versus **12 a**, entries 7, 12). Di‐substitution in *p*‐ and *m*‐position was well accepted (*p*‐OH and *m*‐OMe, ferulic acid, **17 a**, c up to >99 %; *p*‐ and *m*‐OMe, **19 a**, c >99 %, entries 17, 19) as long as the *m*‐substituent was not too e^−^‐pushing (*p*‐ and *m*‐OH, caffeic acid, **18 a**) which led to a significant drop in conversion (c=33 %, entry 18) correlating with the results from above. The *p*‐naphthyl derivatives (**30 a** and **31 a**) which formally correspond to a *p*‐/*m*‐di‐substitution with weak e^−^‐donating groups were excellent substrates, which were quantitatively decarboxylated (c >99 %, entries 30, 31). The size as well as the electronic nature of the *o*‐substituents seem to play a crucial role which were well tolerated as long as they were small (F, **20 a**, c=82 %, entry 20; F, **25 a**, c >81 %, entry 25; Me, **16 a**, c >99 %, entry 16). Sterically more demanding methoxy‐ (**21 a**, c=36 %; **22 a**, c=31 %, entries 21, 22) and nitro‐groups (**14 a**, c up to 10 %, entry 14) were less favoured which also applies to polar (strong e^−^‐donating) *o*‐substituents such as OH (**13 a**, c up to 8 %, entry 13) and led to a complete loss of activity in case of two polar (*o*‐ and *p*‐OH) groups (**49**, Figure 1). Tri‐substituted compounds with functional groups significantly larger than a F‐atom were poor substrates (sinapic acid, **23 a**, c=3‐15 %, entry 23; **24 a**, c=5‐8 %, entry 24).

The substrate profiling was further extended to α,β‐unsaturated carboxylic acids containing *O*‐, *S*‐ and *N*‐heteroaromatic systems at C3. The enzymes were excellent catalysts for the decarboxylation of 2‐furyl‐ (**26 a**) and 2‐thienyl acrylic acid (**27 a**) furnishing the corresponding vinyl products in up to >99 % conversion. *An*FDC^UbiX^ was also capable of decarboxylating the imidazole‐derivative **28 a** albeit with very low rate (c=5 %, entry 28), which is presumably caused by the high degree of protonation (∼90 %/100 %) at pH 6.0/7.5 creating a positive charge. The bicyclic indole‐derivative (**29 a**) was reasonably well accepted (c up to 42 %, entry 29).

In contrast to PADs which did not accept substitution (e. g. Me‐group) at the α‐ or β‐carbon atom to the carboxylate, FDCs showed a more relaxed behaviour tolerating small groups at these positions (α‐F, **32 a**, c up to >97 %; α‐Me, **33 a**, c=20–60 %; β‐Me, **34 a**, c=50–85 %; entries 32–34), whereas bulky substituents led to a marked decrease (α‐NHCOMe, **35** **a**, c=3‐6 %, entry 35) or even loss of FDC activity (α‐Ph, **50**, Figure 1).

In general, compounds lacking a C=C‐spacer between the carboxylate and the aromatic system were not converted (compound **38**–**44**, Figure 1).

Conjugated 2,4‐di‐unsaturated acids **36 a** (sorbic acid) and **37 a** were excellent substrates, which were quantitatively decarboxylated into the corresponding 1,3‐dienes by whole cells and only a minor decrease in rates were observed with isolated enzymes. The acceptance of unsaturated substrates lacking an aromatic system by FDCs constitutes a valuable extension of the substrate portfolio in the bio‐decarboxylation.

However, α,β‐mono‐unsaturated and 2,6‐dienoic acids were unreactive, regardless of their open‐chain (**52**–**54**, **57**) or cyclic structure (**55**, **56**). Likewise, acetylenic substrates (**58**–**60**) and symmetrical (*E,E)*‐muconic acid (**47**) did not react. A switch of the C=C‐bond configuration from (*E*) to (*Z*) (**51**) resulted in substrate rejection (Figure 1).

The results from Scheme 2, Table 1 and Figure 1 reveal a clear substrate structure‐activity pattern of the FDCs enzymes:

i) Minimal substrate requirements consist of an acrylic acid moiety with an extended π‐system in the β‐position, which is fulfilled by an aromatic system or a (minimal) second conjugated C=C bond.

ii) Compounds lacking an α,β‐C=C bond, which is an essential requirement to undergo 1,3‐dipolar cycloaddition with the prFMN cofactor, are unreactive, as well as acetylenic analogs.

iii) The (*E*) or (*Z*) configuration of the reactive C=C bond seems to be critical.

iv) Sterically demanding groups impede reaction rates.

v) Strongly electron‐donating groups impede reaction rates.

### Structural and Mechanistic Aspects

Azomethine ylides have been characterised as dipoles with pronounced nucleophilic character.^46^ Due to their inherent reactivity, they are usually prepared *in situ,* for example by ring‐opening of aziridines.^47^,^48^ Initial cycloadduct formation in the reaction mechanism of FDC is expected to proceed through interaction between the HOMO of prFMN and the substrate's LUMO.^49^ Thus, potential substrates must show a somewhat ambiguous character: the α,β‐unsaturated carboxylic acid molecule must be electrophilic enough to allow cycloadduct formation with the nucleophilic cofactor in the first place. However, after decarboxylation, the cycloadduct should dissociate easily into the olefinic decarboxylation product and cofactor, allowing a new catalytic cycle to initiate. This suggests that decarboxylation itself (the loss of one EWG as CO_2_) is the crucial step that raises electron density in the substrate‐cofactor adduct, promoting it to undergo cyclo‐elimination. Strongly electron‐deficient dipolarophiles are potent mechanistic inhibitors of FDC enzymes, which has been demonstrated experimentally.^35^ Additionally, the enzyme only accepted substrates with an extended π‐system conjugated to the acrylic acid moiety. This preference ensures diffuse electron density in both cofactor and substrate, which allows enhanced matching orbital energy levels according to HSAB and FMO principles.^50^, ^51^, ^52^, ^53^ These considerations are in excellent agreement with the observed substrate preference of FDC enzymes.

**Figure 2:**
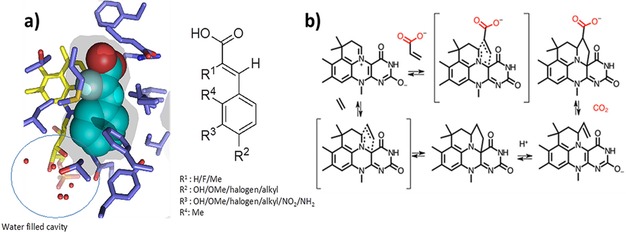
Mechanism and substrate scope of ferulic acid decarboxylases (FDCs). a) Active site of *Aspergillus niger* FDC (*An*FDC) in complex with α‐fluorocinnamic acid (PDB code 4ZAB). A transparent surface reveals the solvent accessible surface on the *re* side of the prFMN that is complementary in shape to the substrate. In addition, a water filled cavity is present near the cofactor ribityl moiety (indicated by circle), providing ample space for *m*‐ and *p*‐substitutions of the aromatic ring. Potential steric constraint occurs with cinnamic acid derivatives bearing bulky substituents at the α‐carbon (R^1^) to the carboxylate or *o*‐substitutions of the aromatic ring (R^4^). b) A general mechanism proposed for reversible decarboxylation of acrylic acid derivatives by prFMN in FDC enzymes via 1,3 dipolar cycloaddition.

An analysis of the *An*FDC active site architecture provides a rationale for FDC tolerance to cinnamic acid residues bearing small substituents (Figure 2a, R^1^=F/Me) at the α‐carbon to the carboxylate (Figure 2). The orientation of the substrate in the active site positions R^2^ and R^3^ substituents at a water filled cavity (Figure 2a), indicating that large groups can be accommodated at the *m*‐ and *p*‐positions of the aromatic ring. In contrast, the *An*Fdc1 structure highlights potential steric constraint with large R^1^ substituents and *o*‐substitutions of the aromatic ring (R^4^). These predictions are in excellent agreement with biotransformation data presented in Table 1.

### Catalytic Performance

To assess the relative activity of the three FDCs, we determined the total turnover numbers for 7 representative substrates. *An*FDC^UbiX^ displayed a high rate indicated by a turnover frequency (TOF) of 370 min^−1^ for cinnamic acid (**1 a**). In general, the enzymes showed highest activity towards acrylic acid derivatives bearing either an unactivated phenyl (**1 a**) or naphthyl group (**31 a**, Table 2, entries 1, 7), affording a total turnover number of up to 55,000 for these substrates. This activity value compares favourably with other industrially relevant enzymatic reactions.^54^,^55^ *Sc*FDC^UbiX^ and *An*FDC^UbiX^ displayed superior activity for the decarboxylation of cinnamic acid (**1 a**), however, the activity towards naphthylacrylic acid (**31 a**) were comparable for the three enzymes. Although *Cd*FDC^UbiX^ exhibited comparatively the lowest activity towards cinnamic acid (**1 a**) and **5 a** (*p*‐Me‐derivative, entry 2), it was the superior catalyst in the decarboxylation of *p*‐coumaric acid (**7 a**), ferulic acid (**17 a**) and the *O*‐heterocyclic derivative (**26 a**). *Sc*FDC displayed the highest tolerance to intensified reaction conditions, showing high activity even at increased substrate loading of **1 a** up to 100 mM. However, at >60 mM of **1 a,** decrease in reaction rate was observed, owing to substrate or product inhibition.

**Table 2:** Comparison of FDCs catalytic activity for representative substrates

**Entry**	Substrates	Total turnover number		
		*An*FDC^UbiX^ ×10^3^	*Sc*FDC^UbiX^ ×10^3^	*Cd*FDC^UbiX^ ×10^3^
**1**	**1 a**	33.0±4.0	55.0±0.5	8.0+1.3
**2**	**5 a**	11.0±0.1	11.0±0.2	6±0.5
**3**	**7 a**	4.0±0.8	5.1±0.4	7.5±0.2
**4**	**17 a**	4.2±0.6	5.2±0.3	10.0±0.4
**5**	**26 a**	6.3±0.8	6.0±1.0	11.0±0.2
**6**	**27 a**	6.3±0.5	11±2.0	11.0±1.0
**7**	**31 a**	17.0 ±0.2	13.0±0.2	15.0±0.10

Reaction conditions: substrate (20–1100 mM), purified enzyme (0.2 mg mL^−1^), NaP_*i*_ buffer (200 mM, pH 7.5), 30 °C, 180 rpm, 8 h. Total turnover numbers were calculated from conversions after 8 h incubation. Reactions were run in triplicate and errors represent the standard deviation from the mean.

### Co‐solvent Compatibility and Upscaling

**Figure 3:**
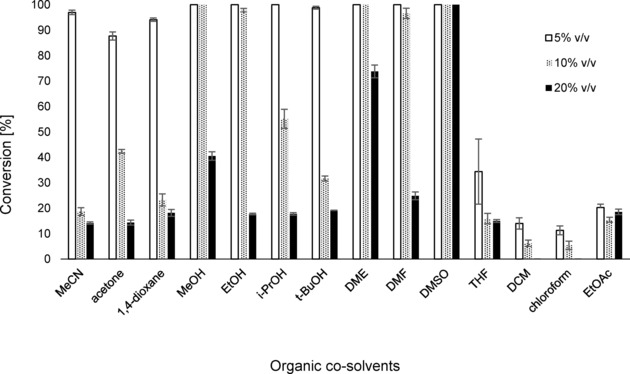
Decarboxylation of sorbic acid (**36 a**) by *Sc*FDC in the presence of organic solvents. Reaction conditions: NaP_i_ (100 mM, pH 6.0), whole lyophilised cells of *E. coli* containing *Sc*FDC (30 mg mL^−1^), substrate (10 mM), organic co‐solvents (5–20 % v/v), 30 °C, 120 rpm, 18 h. Conversions were determined by calibrated RP‐HPLC.

In order to overcome solubility problems of lipophilic substrates or products, the compatibility of *Sc*FDC with organic co‐solvents was tested using 14 water‐miscible and ‐immiscible (co‐)solvents at concentrations of 5 %, 10 % and 20 % v/v (Figure 3). While water‐immiscible biphasic systems containing dichloromethane, chloroform or ethyl acetate led to significant enzyme deactivation, water‐miscible co‐solvents were tolerated surprisingly well at 5 % v/v. MeOH, EtOH, 1,2‐dimethoxyethane and DMF could be employed at 10 % v/v and DMSO was even compatible at 20 % v/v.

In order to prove the applicability of this method on preparative scale, the decarboxylation of ferulic acid (**17 a**) was performed. The substrate load was increased from 10 to 16.8 mM in 20 mL reaction volume. HPLC‐analysis revealed incomplete conversion of the starting material (48 %). The product was isolated by extraction of the aqueous phase with EtOAc and was purified by flash chromatography yielding 19 mg (38 % yield) of **17 b**. Product identity and purity were confirmed by NMR spectroscopy (see Supporting Information).

### Carboxylation Experiments

**Scheme 3:**
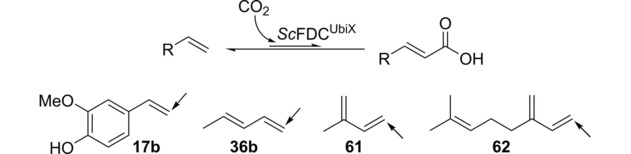
Substrates tested in the carboxylation direction with *Sc*FDC (conversions <1 %). The arrow indicates the expected carboxylation site. Reaction conditions using KHCO_3_: NaP_i_ (100 mM, pH 5.5), whole lyophilised cells of *E. coli* containing *Sc*FDC (30–50 mg mL^−1^), 10 mM substrate (**17 b**, **36 b**, **61**, **62**), KHCO_3_ (0.5–3 M), 30 °C, 120 rpm, 18–20 h, 5–20 % v/v DMSO or DME. Reaction conditions using CO_2_ (gas): NaP_i_ (250 mM, pH 7.5), whole lyophilised cells of *E. coli* containing *Sc*FDC (100 mg mL^−1^), 10 mM substrate (**17 b**), 30 bar CO_2_, 30 °C, 50 rpm, 18 h, 5 % v/v DMSO. Conversions were determined by calibrated RP‐HPLC.

Converting the decarboxylation of acrylic acid derivatives into the reverse carboxylation reaction has been demonstrated for *o*‐(de)carboxylases,^54^, ^55^, ^56^ phenolic acid (de)carboxylases^26^ and pyrrole‐2‐carboxylate^59^, ^60^, ^61^ or indole‐3‐carboxylate (de)carboxylases.^62^ 3‐Methoxy‐4‐hydroxystyrene (**17 b**), 1,3‐pentadiene (**36 b**) and two further 1,3‐dienes (**61**, **62**), which fulfil the minimal substrate requirements for *Sc*FDC, were subjected to carboxylation with *Sc*FDC using elevated concentrations of bicarbonate (0.5–3 M), as well as pressurized CO_2_ (30 bar) as CO_2_‐source (Scheme 3). Using varying amounts of *Sc*FDC preparation (30–50 mg mL^−1^ lyophilised cells) and DMSO (5 – 20 % v/v) as co‐solvent, no formation of the desired products was observed after 18 h.

Enhanced biocatalyst loading (100 mg whole cells mL^−1^) and CO_2_ (30 bar) produced small amounts of **17 a** from **17 b** within 18 h (c<1 %). Although this might be taken as proof‐of‐principle for the carboxylation of alkenes with *Sc*FDC, the reaction was plagued by decomposition of (sensitive) vinylphenol **1 b**, dimerization of structurally similar 4‐vinylphenol (**7 b**) has been reported.^63^ Using 1,3‐pentadiene (**36 b**), isoprene (**61**) or myrcene (**62**) did not result in any formation of carboxylation product using KHCO_3_.

## **Experimental Section**

### General

Commercially available chemicals and reagents of the highest purity were purchased from Sigma‐Aldrich (Poole, Dorset, UK) unless stated otherwise. Compounds **2 a**, **48**, **51**, **36 a**, **34 b**, **27 a**, **6 a**, **38**, **61** were donated by BASF (Ludwigshafen, Germany); **16 a** was obtained from abcr; **5 b**, **52**, **59**, **62** were purchased from Fluka; **8 a** and **8 b** were sourced from Lancaster and **7 a** and **7 b** were purchased from Alfa Aesar (Karlsruhe, Germany), while **58** was purchased from Acros Organics (Geel, Belgium). HPLC solvents were obtained from Sigma‐Aldrich (Poole, Dorset, UK) or ROMIL (Waterbeach, Cambridge, UK) and GC gases from BOC gases (Guildford, UK).

#### (a) Production and Preparation of Biocatalysts

Cloning, expression and purification of *An*FDC^UbiX^, *Sc*FDC^UbiX^ and *Cd*FDC^UbiX^ were performed as previously described.^26^,^30^ The purified enzymes were either snap‐frozen or stored at −80 °C until when needed or lyophilised and stored at −20 °C. For the preparation of the whole cell biocatalysts, cultivation was performed in 500 mL LB broth medium with kanamycin (30 μg mL^−1^) and ampicillin (50 μg mL^−1^). Cultures were initially incubated at 37 °C with shaking at 200 rpm. At an optical density (OD_600_) between 0.6 and 0.8, isopropyl β‐D‐1‐thiogalactopyranoside (IPTG) was added to a final concentration of 0.3 mM to induce protein expression and MnCl_2_ to the final concentration of 1 mM was added. Incubation was continued at 20 °C and 250 rpm for 18 h. Cells were then harvested by centrifugation and suspended in sodium phosphate buffer (100 mM, pH 7.5). The harvested cells were used as fresh resting cells or lyophilised preparation.

#### (b) General Procedure for Isolated Enzyme Decarboxylation

For FDC^UbiX^‐catalysed decarboxylation reaction using purified enzyme preparation, a 500 μL reaction mixture contained carboxylic acid substrate (5 mM), 2–10 % (v/v) DMSO, purified FDC^UbiX^ (0.2 mg mL^−1^) in sodium phosphate buffer (100 mM, pH 7.5). Reaction mixtures in 2 mL tightly‐closed glass vials were incubated at 30 °C with 180 rpm shaking for 18 h, after which the enzyme was inactivated by the addition of an equal volume of MeCN and vigorously mixed. The reaction mixtures were centrifuged (4 °C, 2,831 rcf, 5 min); the clear supernatant was filtered and analysed by reverse phase HPLC. Where analysis of biotransformation was performed on the GC‐MS, an equal volume of EtOAc (containing a known concentration of an internal standard where necessary) was added to biotransformation mixture, vigorously mixed, centrifuged and the organic layer was extracted twice. The aqueous layer was then acidified to a pH of ∼2 and further extracted with EtOAc with centrifugation (4 °C, 2,831 rcf, 5 min) to improve the separation of phases. The organic layers were combined and dried over anhydrous MgSO_4_ and samples were analysed by GC‐MS.

#### (c) General Procedure for whole‐cell Decarboxylation

Lyophilised whole cells of *E. coli* (30 mg) containing overexpressed *Sc*FDC were rehydrated for 30 min at 30 °C with 120 rpm shaking in phosphate buffer (950 μL, 100 mM, pH 6.0) in 1.5 mL plastic Eppendorf tubes. Substrates were supplied by adding 50 μL of 200 mM stock solution in DMSO to achieve a substrate concentration of 10 mM in 1 mL of total reaction volume, followed by incubation for 18 h at 30 °C with shaking in horizontal position at 120 rpm under exclusion of light. For substrates showing limited solubility (**48**, **31 a**), lyophilised cells were suspended in buffer (800 μL) and after rehydration, pure DMSO (150 μL) was supplemented followed by addition of a substrate stock (50 μL) and incubation. After given reaction time, samples were centrifuged at 14,000 rpm for 10 min and supernatant (100 μL) was diluted with 900 μL of H_2_O/MeCN/trifluoroacetic acid (TFA, 50 : 50 : 3) to precipitate residual protein. The diluted sample was centrifuged again, followed by analysis with HPLC. All reactions were performed in triplicate plus negative control without lyophilised cells.

### Co‐solvent Studies

Stock solutions of **36 a** (200 mM) were prepared in MeCN, acetone, 1,4‐dioxane, MeOH, EtOH, *i*‐PrOH, *t*‐BuOH, DME, DMF, DMSO, THF, DCM, chloroform and EtOAc. Lyophilised cells were rehydrated in 800, 900 or 950 μL phosphate buffer (100 mM, pH 6.0). 50 μL of the corresponding stock solution was added to the mixture and pure co‐solvent was added to achieve a reaction volume of 1 mL, followed by incubation. For water‐miscible co‐solvents, sample workup and analysis was performed as described above. For immiscible solvents, partial evaporation of the organic layer was observed and therefore, only the aqueous phases were analysed using HPLC.

#### (d) General Procedure for Carboxylation using KHCO_3_

Lyophilised cells (30–50 mg) were rehydrated in phosphate buffer (800–950 μL, 100 mM, pH 5.5). Pure co‐solvent (0–150 μL) followed by substrate stock (50 μL; **17 b**, **36 b** and **61** 200 mM in DMSO or **62** 200 mM in DME) was added to achieve a reaction volume of 1 mL, followed by transfer of the mixture into a screw‐neck glass vial containing KHCO_3_ (0.5–3 M). The vessels were swiftly closed to avoid the loss of emerging CO_2_ gas and were incubated for 18–20 h.

#### (e) General Procedure for Carboxylation using Pressurized CO_2_

Lyophilised cells (300 mg) were rehydrated in phosphate buffer (2850 μL, 250 mM, pH 7.5). 150 μL of a 200 mM stock solution of **17 b** in DMSO was added and the mixture was transferred into a steel pressure vessel equipped with a stirring bar. The reaction mixture was pressurized with technical CO_2_ gas (30 bar) and was stirred (50 rpm) at 30 °C for 18 h.

#### (f) Preparative Scale Biotransformation

560 mg lyophilised cells were rehydrated in a plastic vial (50 mL) with phosphate buffer (19 mL, 100 mM, pH 6.0). **17 a** (65.1 mg, 0.34 mmol) and hydroquinone (6.5 mg, 0.06 mmol, radical scavenger to inhibit product decomposition) were dissolved in MeOH (1 mL) and added to the mixture. The vessel was wrapped in aluminium foil to ensure protection from light and was incubated for 24 h with shaking at 120 rpm at 30 °C. Solids were separated by centrifugation at 4000 rpm at 4 °C for 20 min. The supernatant (100 μL) was diluted with H_2_O/MeCN/TFA and was subjected to HPLC analysis, which revealed incomplete turnover of the starting material (48 % conversion). The remaining liquid was extracted with EtOAc (4×20 mL). The combined organic phases were dried with Na_2_SO_4_ and filtered. After evaporation, a mixture of off‐white solids and dark yellow oil was obtained. The oil was diluted with DCM and purified by flash column chromatography (silica gel Merck 60, DCM), giving 19 mg (0.13 mmol, 38 % yield) of spectroscopically pure **17 b** as colourless oil with a distinct clove‐like odour.

**17 b**: ^1^H NMR (300 MHz, DMSO‐*d_6_*): δ=9.09 (s, 1H), 7.04 (d, *J*=1.9 Hz, 1H), 6.85 (dd, *J*=8.1, 1.9 Hz, 1H), 6.72 (d, *J*=8.1 Hz, 1H), 6.60 (dd, *J*=17.6, 10.9 Hz, 1H), 5.63 (dd, *J*=17.6, 1.1 Hz, 1H), 5.05 (dd, *J*=10.9, 1.0 Hz, 1H), 3.78 (s, 3H); ^13^C NMR (75 MHz, DMSO‐*d_6_*): δ=147.69, 146.71, 136.71, 128.79, 119.53, 115.36, 110.95, 109.57, 55.56.^62^,^63^

#### (g) Analyses of whole‐cell Biotransformations

**HPLC analysis: HPLC/UV experiments** were performed on a HPLC Agilent 1260 Infinity system with a diode array detector and a reversed‐phase Phenomenex Luna C18 column (100 Å, 250×4.6 mm, particle size 5 μm, column temperature 24 °C). All compounds were spectrophotometrically detected at 220, 254, 263, 280 and 310 nm, respectively. Method was run over 22 min with H_2_O/TFA (0.1 %) as the mobile phase (flow rate 1 mL min^−1^) and a MeCN/TFA (0.1 %) gradient (0–2 min 5 %, 2–15 min 5–100 %, 15–17 min 100 %, 17–22 min 100–5 %). Conversions were determined by comparison with calibration curves for products and substrates prepared with authentic reference material. Due to the instability of the decarboxylation products, concentrations were determined indirectly *via* the reduction of substrate peaks.

**Headspace GC‐MS analysis**: To verify the formation of volatile decarboxylation products not detectable on HPLC, reactions were performed in glass vials capped with rubber septa. Volatiles were analysed directly with an Agilent 7697A headspace sampler (oven temp. 80 °C, loop temp. 90 °C, transfer line temp. 100 °C, vial equilibration time 2 min, vial pressurization 15 psi). In addition, a 10 μL syringe (Agilent syringe FN 26/50/cone) was pre‐heated (10 min, 80 °C) to prevent condensation prior to injection. From headspace of reaction vials 9 μL were injected split‐less (for analysis of compound **36 b** and **37 b**). For separation and detection, an Agilent 7890A GC machine (oven temp. 50 °C) with a HP‐5 ms capillary column (30 m×0.25 mm×0.25 μm; stationary phase: bonded and cross‐linked 5 % phenyl‐methylpolysiloxane) equipped with a 5975C mass‐selective detector (electron impact ionisation, 70 eV; quadrupole mass selection) using helium as carrier gas was used.

**NMR analysis**: NMR spectra were recorded with a Bruker AVANCE III 300 MHz spectrometer using a 5 mm BBO probe at 300 K. Chemical shifts (δ) are expressed in ppm, coupling constants (*J*) are given in Hz.

#### (h) Analysis of Purified Enzyme Biotransformations

**GC‐MS analysis** was performed on an Agilent 5977A Series GC/MSD System with an Agilent 7890B Series GC coupled to Mass Selective Detector. Analysis was performed using GC/MSD MassHunter Data Acquisition and ChemStation Data Analysis. A 30 m DB‐WAX column with 0.25 mm inner diameter and 0.25 μm film thickness (Agilent, Santa Clara, CA, USA) was used. Analysis method: Inlet temperature: 240 °C, detector temperature: 250 °C, MS source 230 °C, helium flow: 1.2 mL min^−1^; oven temperature 40–240 °C, 15 °C min^−1^.

**Reverse phase HPLC** was performed on an Agilent system (Santa Clara, CA, USA) equipped with a G1379A degasser, G1312A binary pump, a G1367A well plate autosampler unit, a G1316A temperature controlled column compartment and a G1315C diode array detector. Columns used include: Kinetex C18; 250 mm length, 4.6 mm diameter, 5 μm particle size (Phenomenex, Macclesfield, Cheshire, UK) and Syncronis; C18; 250 mm length, 4.6 mm diameter, 5 μm particle size (Thermo Scientific; Waltham, MA USA).

Substrates standards and product markers, and the resulting biotransformation products were analysed by reverse phase chiral HPLC using isocratic methods with different solvent ratios of MeCN and H_2_O, with 0.1 % TFA as additive. The flow rate was maintained at 1 mL min^−1^ and elutes were detected by the UV detector at a wavelength of 245 nm (except for pyrrole which was monitored at 210 nm). To account for the variation in UV response between the starting material and the product, relative response factors were experimentally determined. Correction factors were calculated from the ratio of the slopes of standard curves plotted for varying concentrations of both the acid and the corresponding alkene at a UV detection wavelength of 245 nm.

## Conclusion

In summary, we elucidated the substrate scope and high activity of FDCs as reversible (de)carboxylation catalysts. The enzymes displayed broad substrate tolerance towards a variety of phenylacrylic acids and heteroaromatic analogues thereof, as well as non‐aromatic 2,4‐dienoic acids. The minimum structural requirement for substrate acceptance is a non‐aromatic or (hetero)aromatic conjugated π‐system linked to C3. The observed substrate‐activity pattern is in agreement with the proposed 1,3‐dipolar cycloaddition mechanism. Steric requirements and the (*E*/*Z*)‐configuration of the acrylic C=C bond had a strong impact on reaction rates. Attempts to reverse the reaction into the carboxylation direction in presence of bicarbonate or pressurized CO_2_ were unsuccessful.

### Associated Content

Data on pH study, co‐solvent compatibility study, analytical protocols including HPLC, GC‐MS analyses and representative traces of biotransformation products are available in the Supporting Information.

## Conflict of interest

The authors declare no conflict of interest.

## Supporting information

As a service to our authors and readers, this journal provides supporting information supplied by the authors. Such materials are peer reviewed and may be re‐organized for online delivery, but are not copy‐edited or typeset. Technical support issues arising from supporting information (other than missing files) should be addressed to the authors.

Supplementary
