# Supplementary material for: Terminal Alkenes from Acrylic Acid Derivatives via Non‐Oxidative Enzymatic Decarboxylation by Ferulic Acid Decarboxylases
Source: ChemCatChem. 2018 Jul 17;10(17):3736–45. doi: 10.1002/cctc.201800643 (PMC6175315; doi:10.1002/cctc.201800643)
Supplement: Supplementary file 1 — Supplementary [file CCTC-10-3736-s001.pdf]

Supporting Information

© Copyright Wiley-VCH Verlag GmbH & Co. KGaA, 69451 Weinheim, 2018

Terminal Alkenes from Acrylic Acid Derivatives via Non-Oxidative Enzymatic Decarboxylation by Ferulic Acid Decarboxylases

Godwin A. Aleku, Christoph Prause, Ruth T. Bradshaw-Allen, Katharina Plasch, Silvia M. Glueck, Samuel S. Bailey, Karl A. P. Payne, David A. Parker, Kurt Faber,* and David Leys*

Supporting Information

Table of Contents

Section S1. Extended data 3

Section S1.1. Biotransformation conditions: effect of pH and buffer systems 3

Section S1.2. Biotransformation with different biocatalyst preparations 4

Section S1.3. pH and temperature profile of *Sc*FDC using lyophilised whole *E. coli*/*Sc*FDC cells 5

Section S1.4. Time study of *Sc*FDC using lyophilised whole *E. coli*/*Sc*FDC cells 6

Section S5. Calculation of total turnover number for FDC-catalysed biotransformation reactions 7

Section S6. Analysis: Chromatograms 8

Section S7. Synthesis of reference material 28

Section S8. Spectra of reference material 30

Section S9. References 44

Section S10. Full list of References from Manuscripts 44

Section S1. Extended data

Section S1.1. Biotransformation conditions: effect of pH and buffer systems

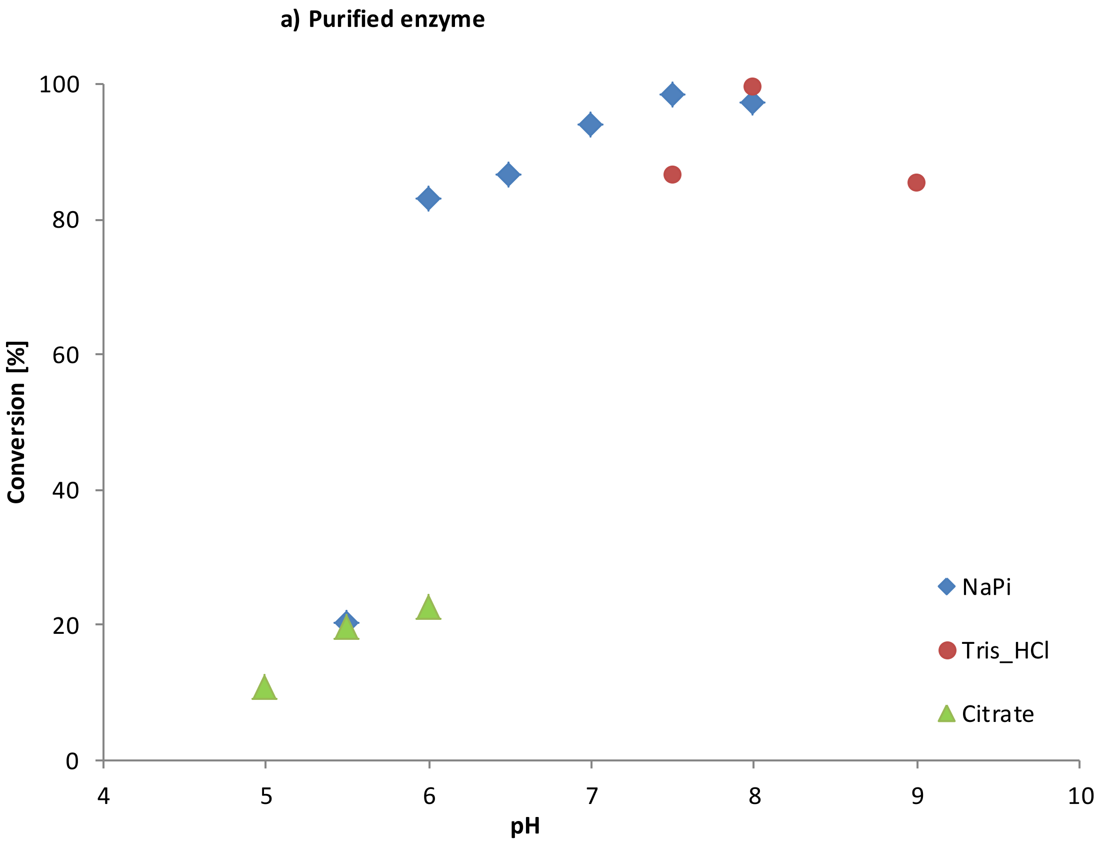

Figure S1. Effect of pH and buffer systems on conversion of *An*FDC^{UbiX}-catalysed decarboxylation of 20 mM cinnamic acid (1a). Data points represent conversions obtained with purified *An*FDC^{UbiX} after 12 h using different buffer systems. Buffers: citrate (100 mM, pH 5.0 – 6.0), sodium phosphate (NaP_i, 100 mM, pH 5.6 – 8.0), Tris-HCl (100 mM, pH 7.5 – 9.0).

Section S1.2. Biotransformation with different biocatalyst preparations

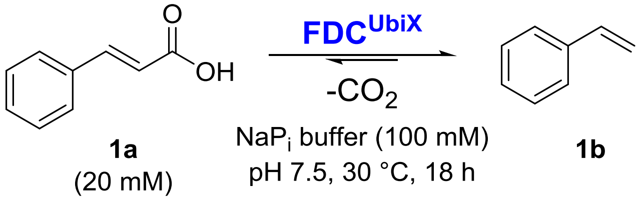

<u>Catalyst loading</u>

a) 0.2 mg mL^{-1} purified or lyophilised pure *An*FDC^{UbiX}
b) 1 mg mL^{-1} lyophilised cell free extract
c) 30 mg mL^{-1} lyophilised whole cells
d) Fresh resting cells at final OD_{600} of 30

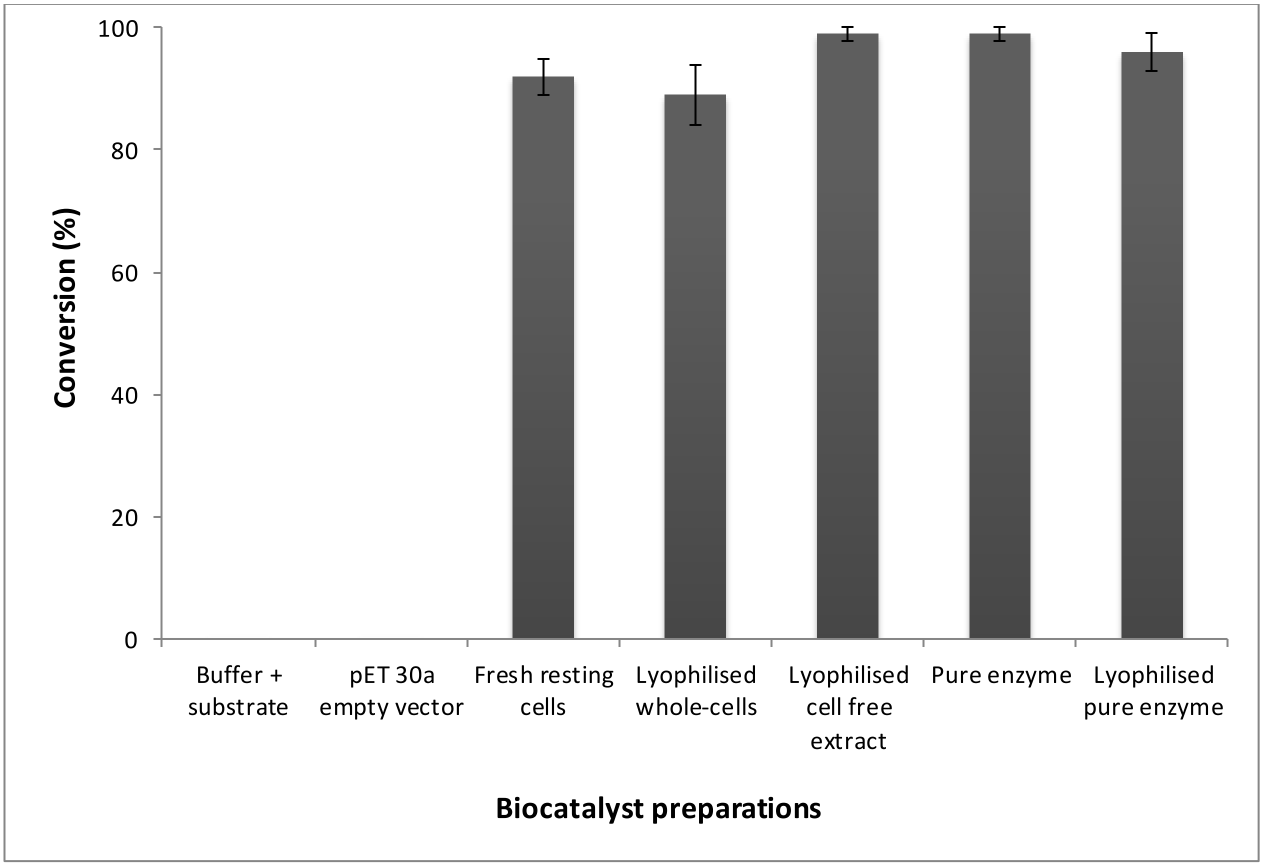

Figure S2. Conversion monitored for the *An*FDC^{UbiX}-catalysed decarboxylation of 20 mM cinnamic acid (1a) using different biocatalyst preparations. Conversions were determined by calibrated RP-HPLC.

Section S1.3. pH and temperature profile of *Sc*FDC using lyophilised whole *E. coli*/*Sc*FDC cells

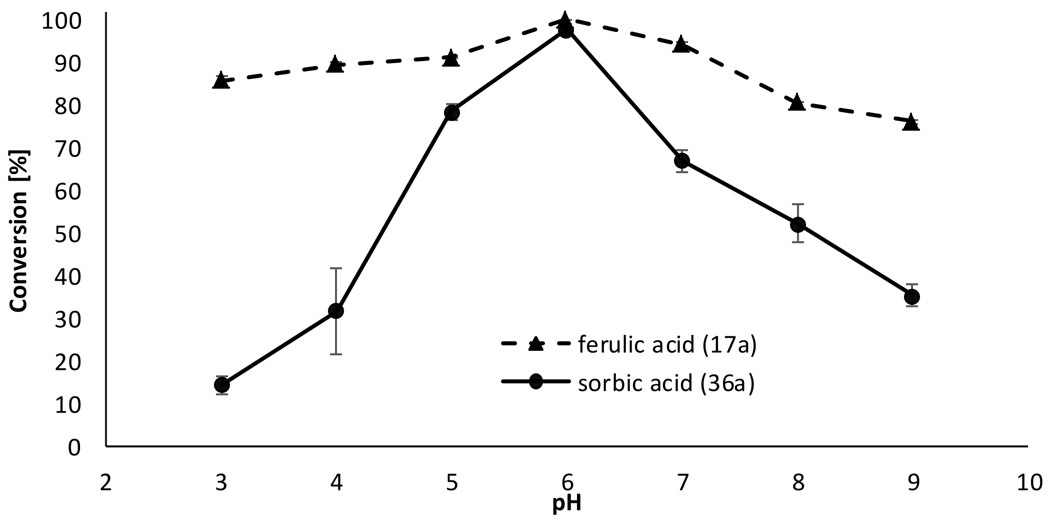

Figure S3. Decarboxylation of ferulic acid (17a) and sorbic acid (36a) at various pH. Reaction conditions: phosphate buffer (100 mM, pH 3.0 – 9.0), whole lyophilised cells of *E. coli* containing *Sc*FDC (30 mg mL^{-1} with 17a and 10 mg mL^{-1} with 36a, resp.), 10 mM substrate, 30 °C, 120 rpm, 18 h, 5% v/v DMSO. Conversions were determined by calibrated RP-HPLC.

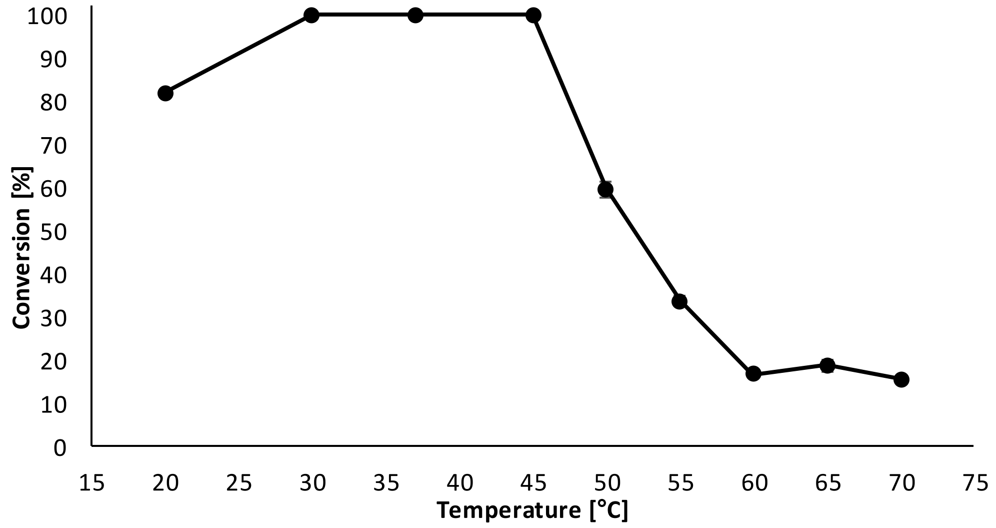

Figure S4. Conversion of sorbic acid (36a) at various temperature. Reaction conditions: phosphate buffer (100 mM, pH 6.0), whole lyophilised cells of *E. coli* containing Fdc1 (30 mg mL^{-1}), 10 mM substrate, 20 – 70 °C, 120 or 300 rpm, 18 h, 5% v/v DMSO. Conversions were determined by calibrated RP-HPLC.

Section S1.4. Time study of *Sc*FDC using lyophilised whole *E. coli*/*Sc*FDC cells

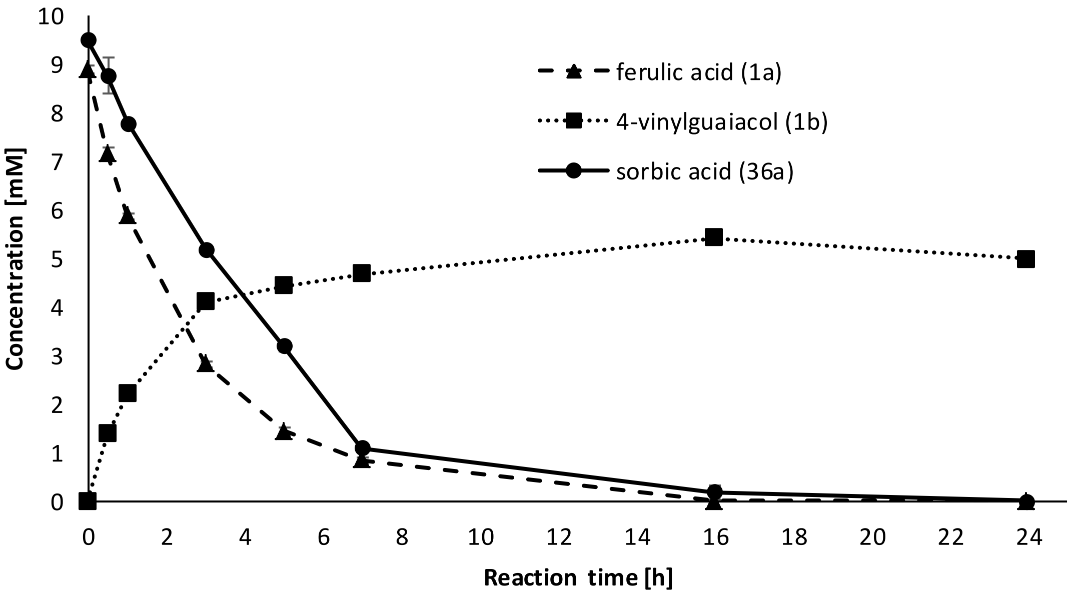

Figure S5. Decarboxylation of ferulic acid (1a) and sorbic acid (36a) over time. Reaction conditions: phosphate buffer (100 mM, pH 6.0), whole lyophilised cells of *E. coli* containing Fdc1 (30 mg mL^{-1}), 10 mM substrate, 30 °C, 120 rpm, 5% v/v DMSO; 0.5 – 24 h (data points at 0 h are the results of control experiments in the absence of biocatalyst). Conversions were determined by calibrated RP-HPLC.

Section S5. Calculation of total turnover numbers (TTN) for FDC-catalysed biotransformation reactions

$$TTN = \frac{mmol\ \ of\ product\ formed}{mmol\ of\ catalyst} \qquad (1)$$

$$mM\ of\ product\ formed = \text{mM of substrate x conversion facto}r \qquad (2)$$

$$mmol\ of\ product = mM\ of\ product\ formed \times\ reaction\ vol\ (L) \qquad (3)$$

(2) in (3)

$$mmol\ of\ product = \text{mM of substrate x conversion facto}r \times\ reaction\ vol\ (L) \qquad (4)$$

$$mmol\ of\ catalyst\ used\ = \frac{mg\ of\ \ enzyme\ used}{total\ Mwt\ per\ active\ site\ (\frac{mg}{mmol})} \qquad (5)$$

(4) and (5) in (1)

$$TTN = \frac{\text{mM of substrate x conversion facto}r \times reaction\ vol\ [L]}{\frac{mg\ of\ enzyme\ used}{total\ Mwt\ per\ active\ site\ [\frac{mg}{mmol}]}} \qquad (6)$$

- Approximate molecular weight of FDC enzyme = 57 kDa = 57 000 g mol^{-1}.
- 0.2 mg of enzyme was used in 1 mL biotransformation reaction.
- Up to 100 mM substrate loading was used.
- Up to 99% conversion was achieved.

Section S6. Analysis: Chromatograms

Representative chromatograms from GC and HPLC analyses of isolated enzyme biotransformation reactions are shown below.

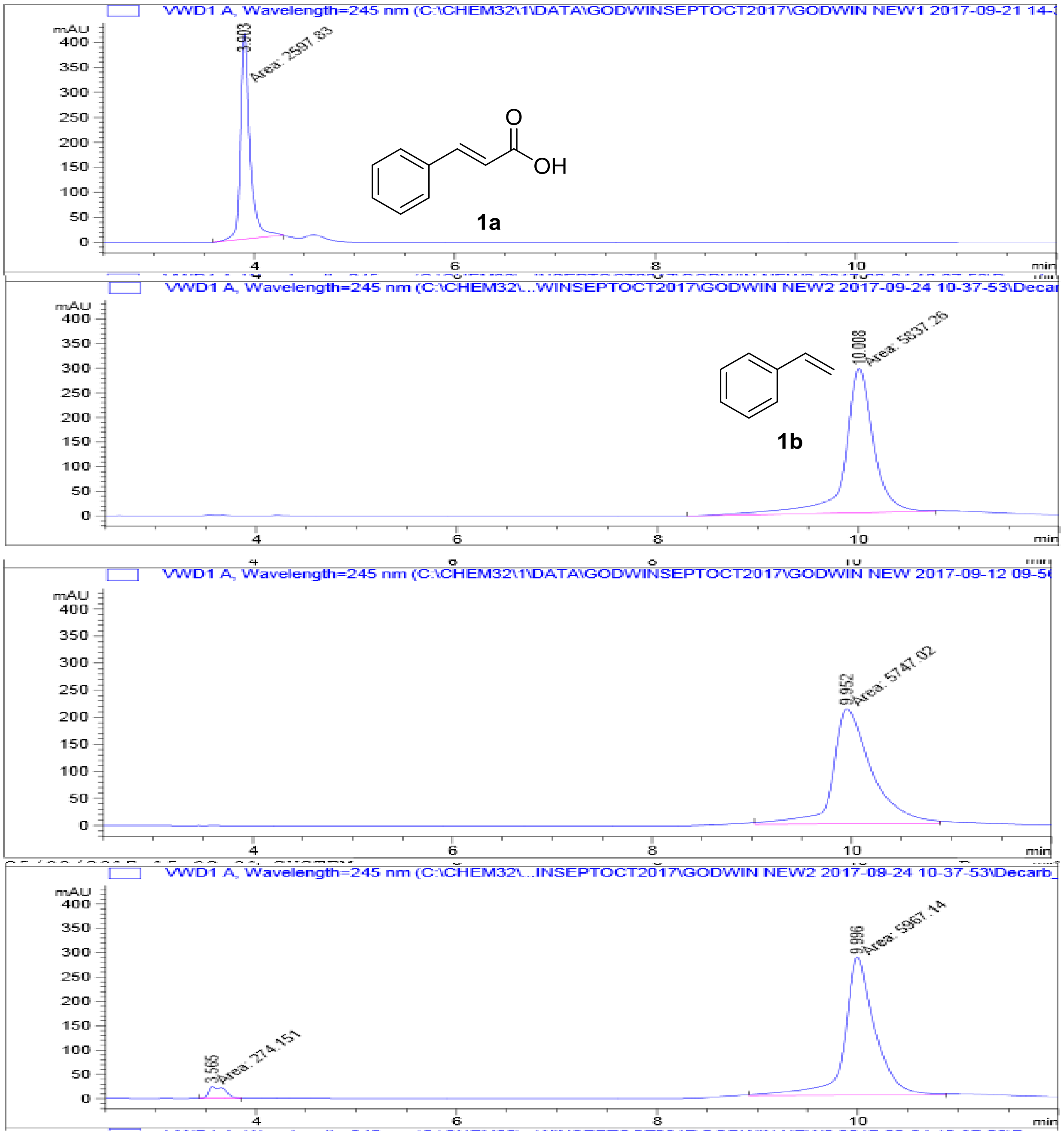

Figure S6. HPLC-analysis: FDC-catalysed biotransformations for the decarboxylation of 1a showing standard of cinnamic acid 1a (row 1), standard of styrene 1b (row 2), biotransformation of 1a by purified *An*FDC^{UbiX} and *Cd*FDC^{UbiX}, respectively (rows 3 & 4).

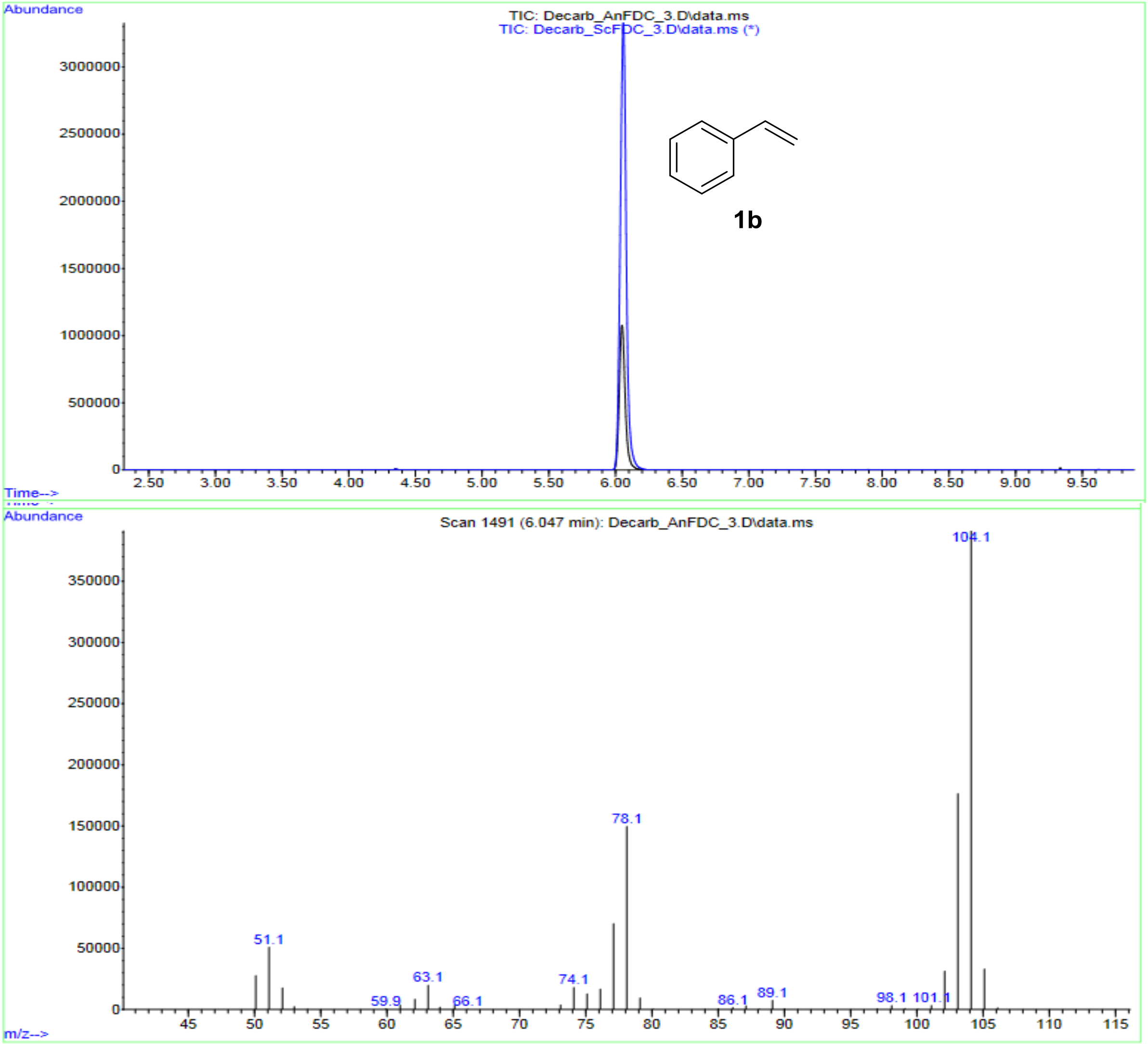

Figure S7. GC-MS-analysis: FDC-catalysed decarboxylation of 100 mM cinnamic acid 1a. Row 1: product of *An*FDC^{UbiX}-catalysed reaction (black) overlaid with product of *Sc*FDC^{UbiX}-catalysed reaction (blue). Row 2: Mass spectrum of product of *An*FDC-catalysed biotransformation. MS calcd. for C_8H_8 (styrene 1b), 104.06, found 104.10.

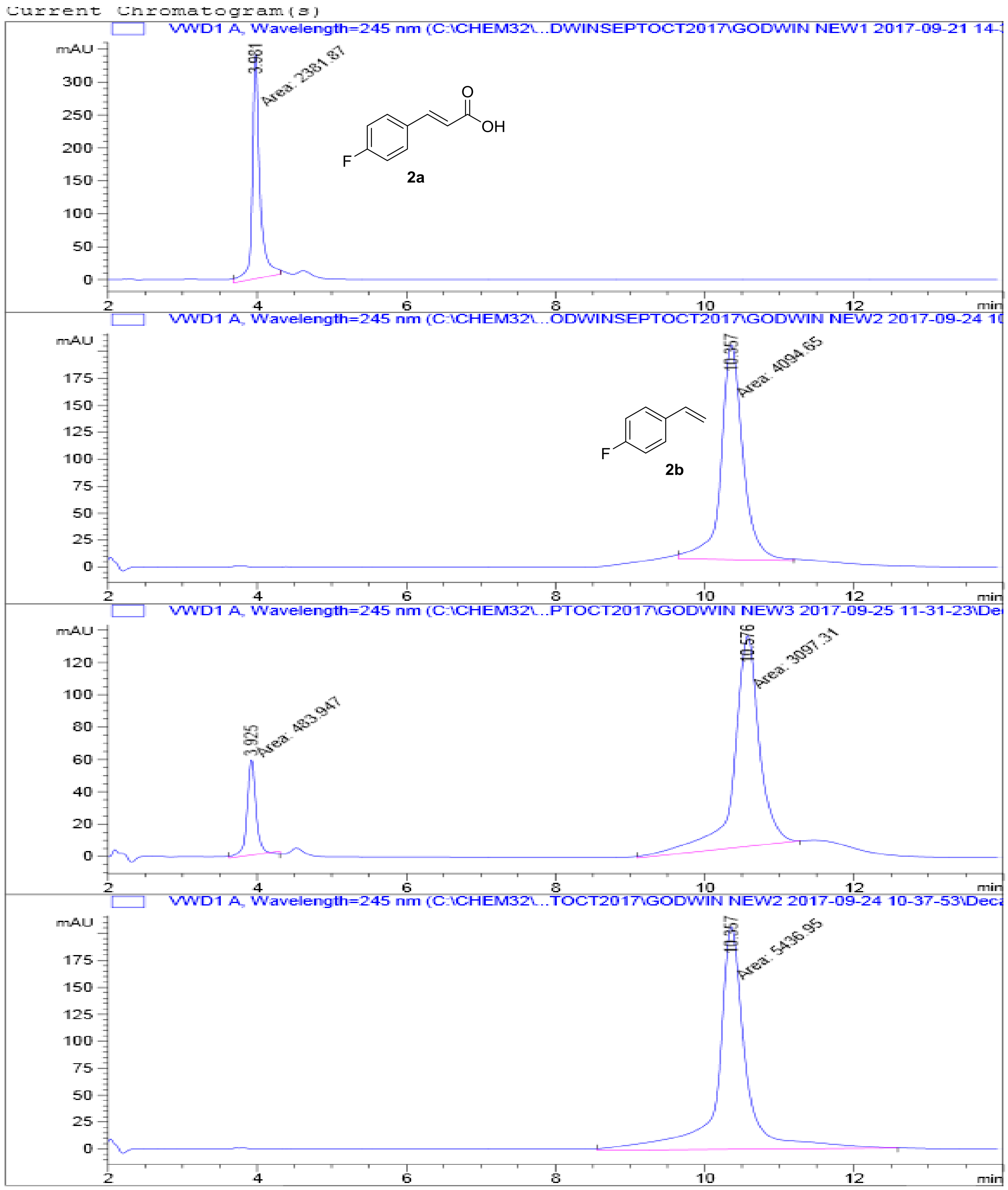

Figure S8. HPLC analysis: FDC-catalysed decarboxylation of *p*-fluorocinnamic acid 2a. Standard of starting material 2a (row 1), product *p*-fluorostyrene 2b (row 2), and traces from biotransformation catalysed by *Cd*FDC^{UbiX} *and An*FDC^{UbiX}, respectively (rows 3 & 4).

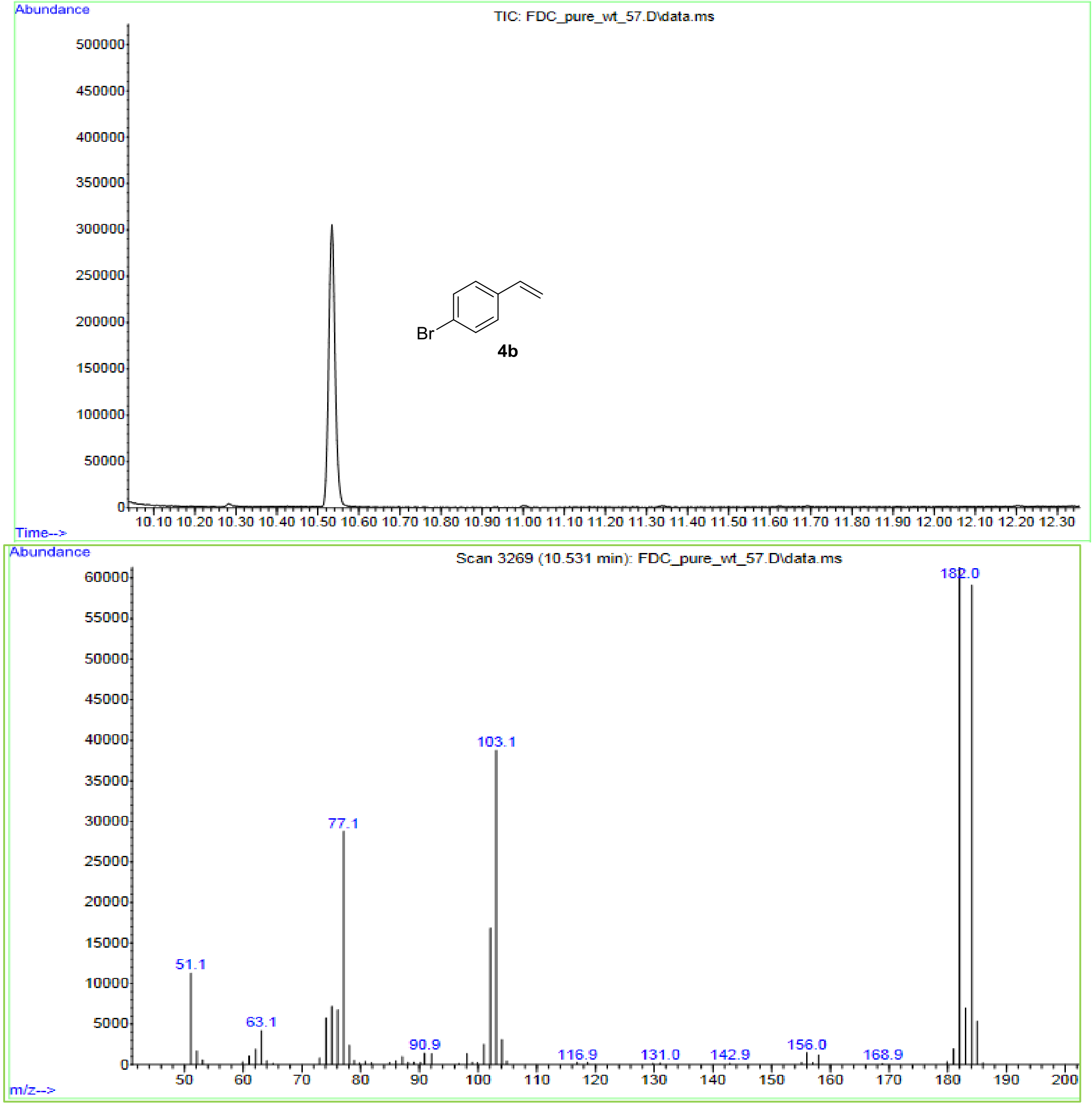

Figure S9. GC-MS-analysis: *An*FDC^{UbiX}-catalysed decarboxylation of *p*-bromocinnamic acid 4a showing the product (top) and a mass spectrum of product of *An*FDC-catalysed biotransformation (bottom). MS calcd. for C_8H_7Br (*p*-bromostyrene 4b) 181.97, found 182.00.

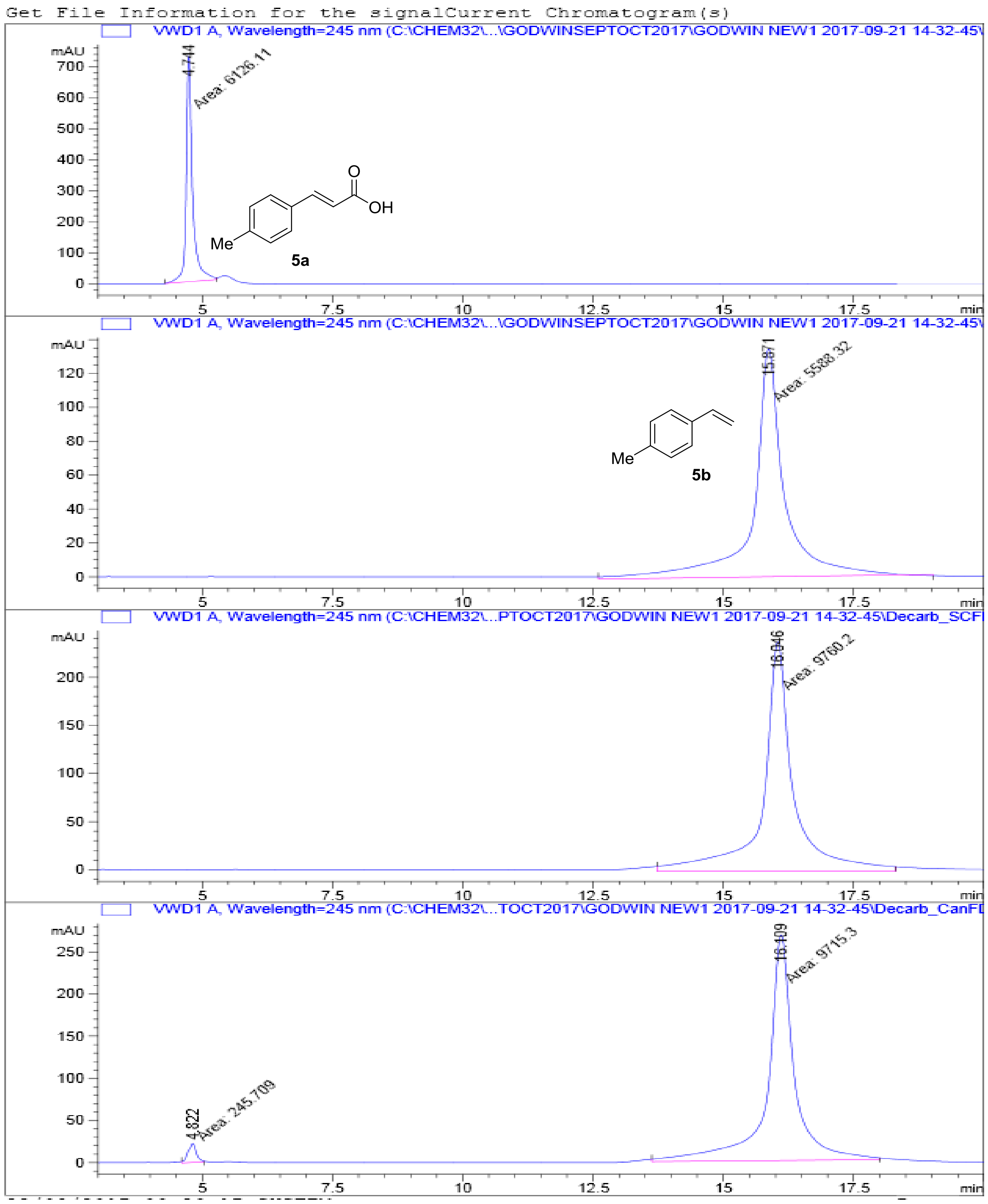

Figure S10. HPLC analysis: FDC-catalysed decarboxylation of *p*-methylcinnamic acid 5a, showing standards of starting material 5a (row 1) and product *p*-methylstyrene 5b (row 2), and biotransformation traces of reactions catalysed by *Sc*FDC^{UbiX} and *Cd*FDC^{UbiX}, respectively.

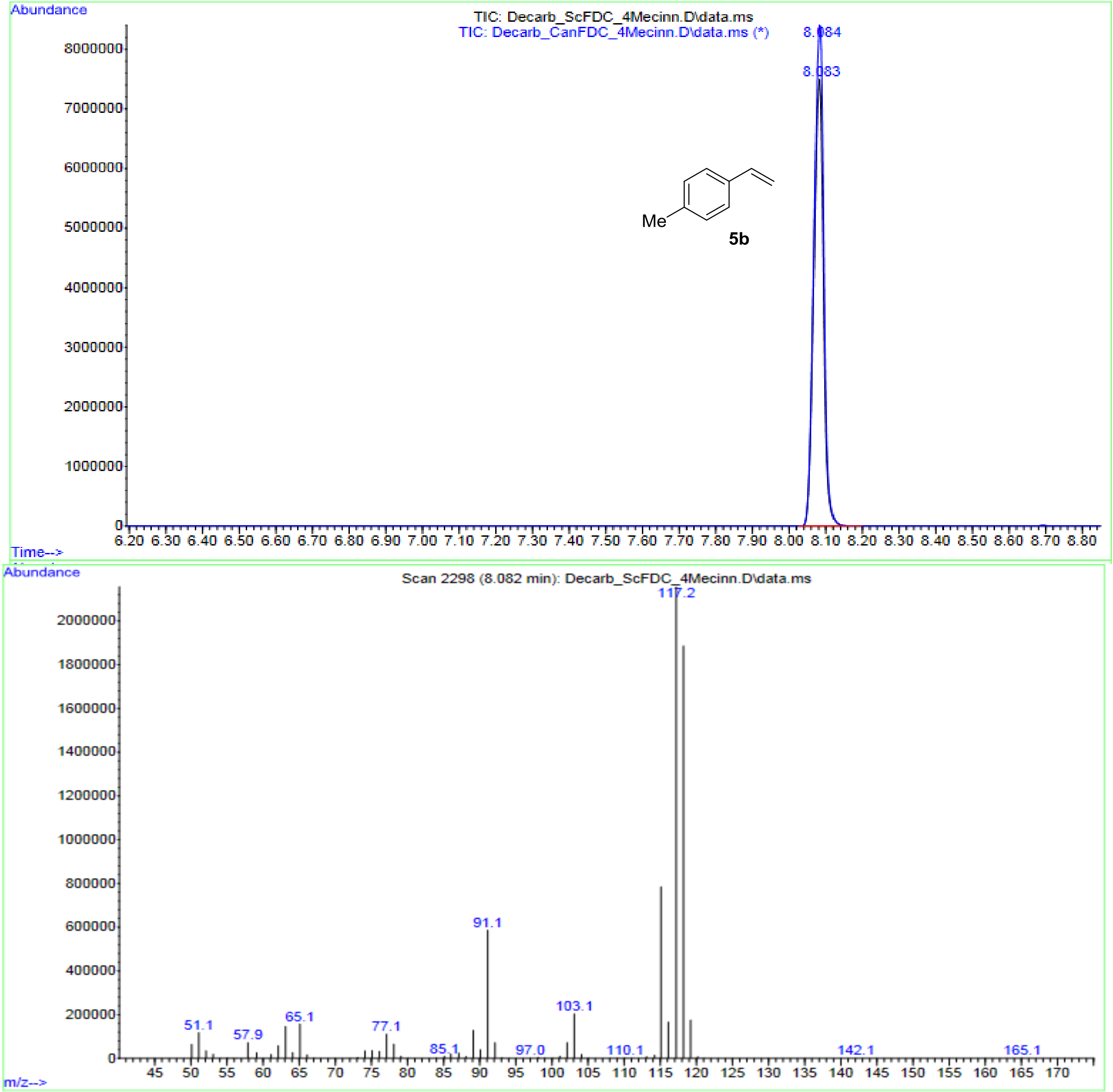

Figure S11. GC-MS-analysis: FDC-catalysed decarboxylation of 40 mM *p*-methyl cinnamic acid 5a showing, Row 1: product of *Sc*FDC^{UbiX}-catalysed reaction (black) overlaid with a trace of *CdF*DC^{UbiX}-catalysed reaction (blue). Row 2: mass spectrum of product of *Sc*FDC-catalysed biotransformation. MS calcd. for C_9H_{10} (*p*-methylstyrene 5b) 118.18, found 117.20.

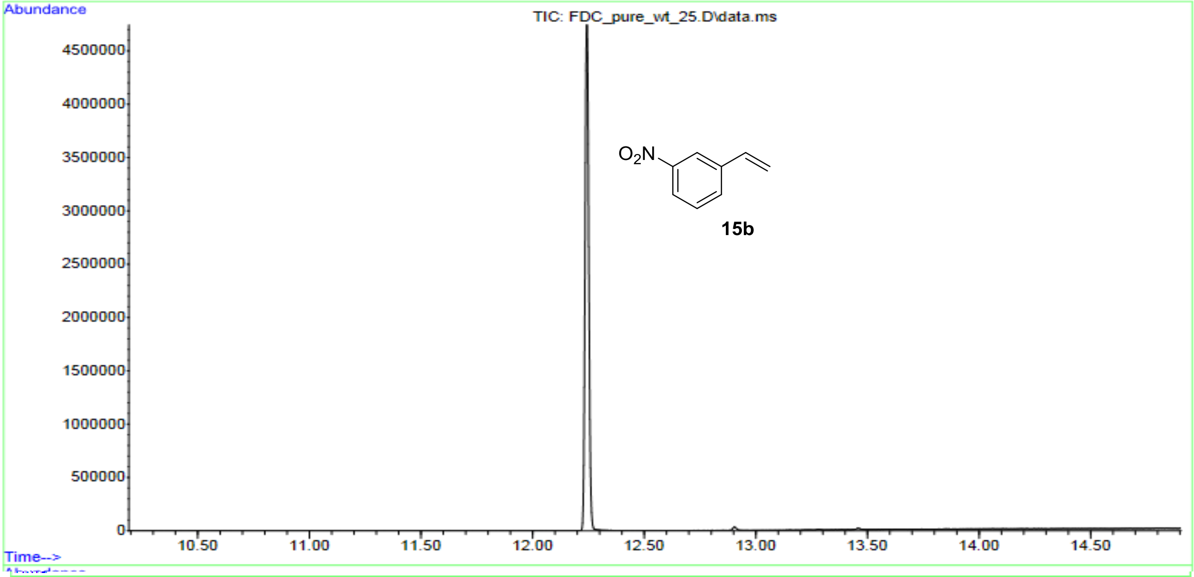

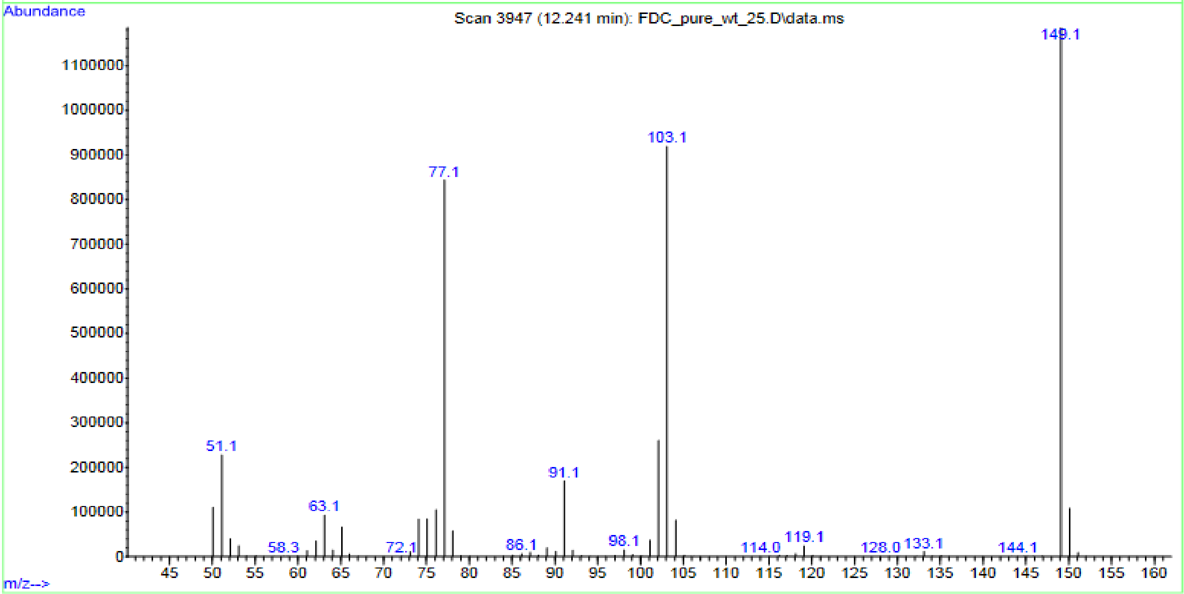

Figure S12. GC-MS-analysis: *An*FDC^{UbiX}-catalysed decarboxylation of *m*-nitrocinnamic acid 15a showing the product (top) and a mass spectrum of product of *An*FDC-catalysed biotransformation (bottom). MS calcd. for $C_8H_7NO_2$ (*m*-nitrostyrene 15b) 149.15, found 149.10.

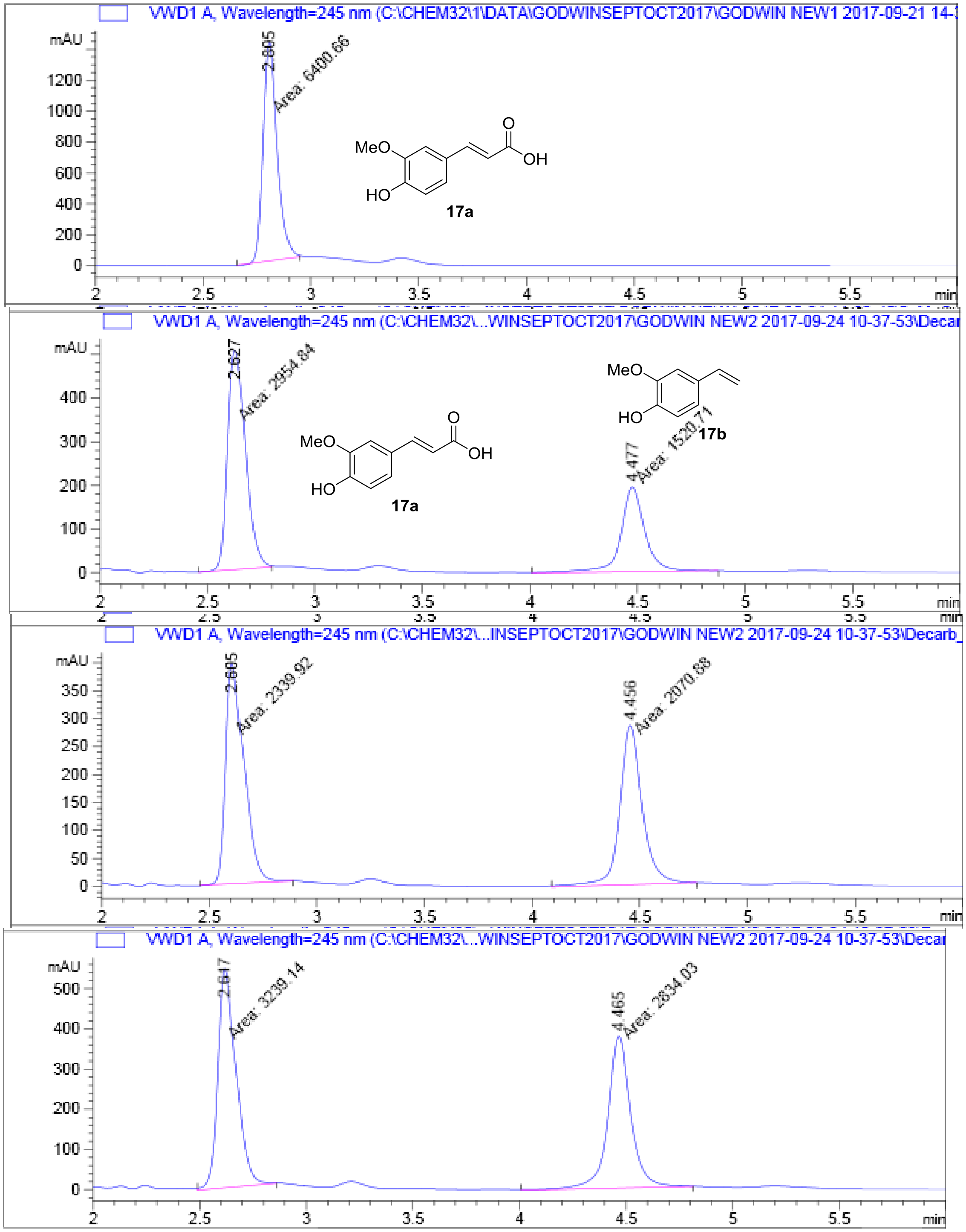

Figure S13. HPLC-analysis: FDC^{UbiX}-catalysed decarboxylation of ferulic acid 17a showing (from top to bottom): standard of 17a, reaction catalysed by *An*FDC^{UbiX}, reaction catalysed by *Sc*FDC^{UbiX} and reaction catalysed by *Cd*FDC^{UbiX}.

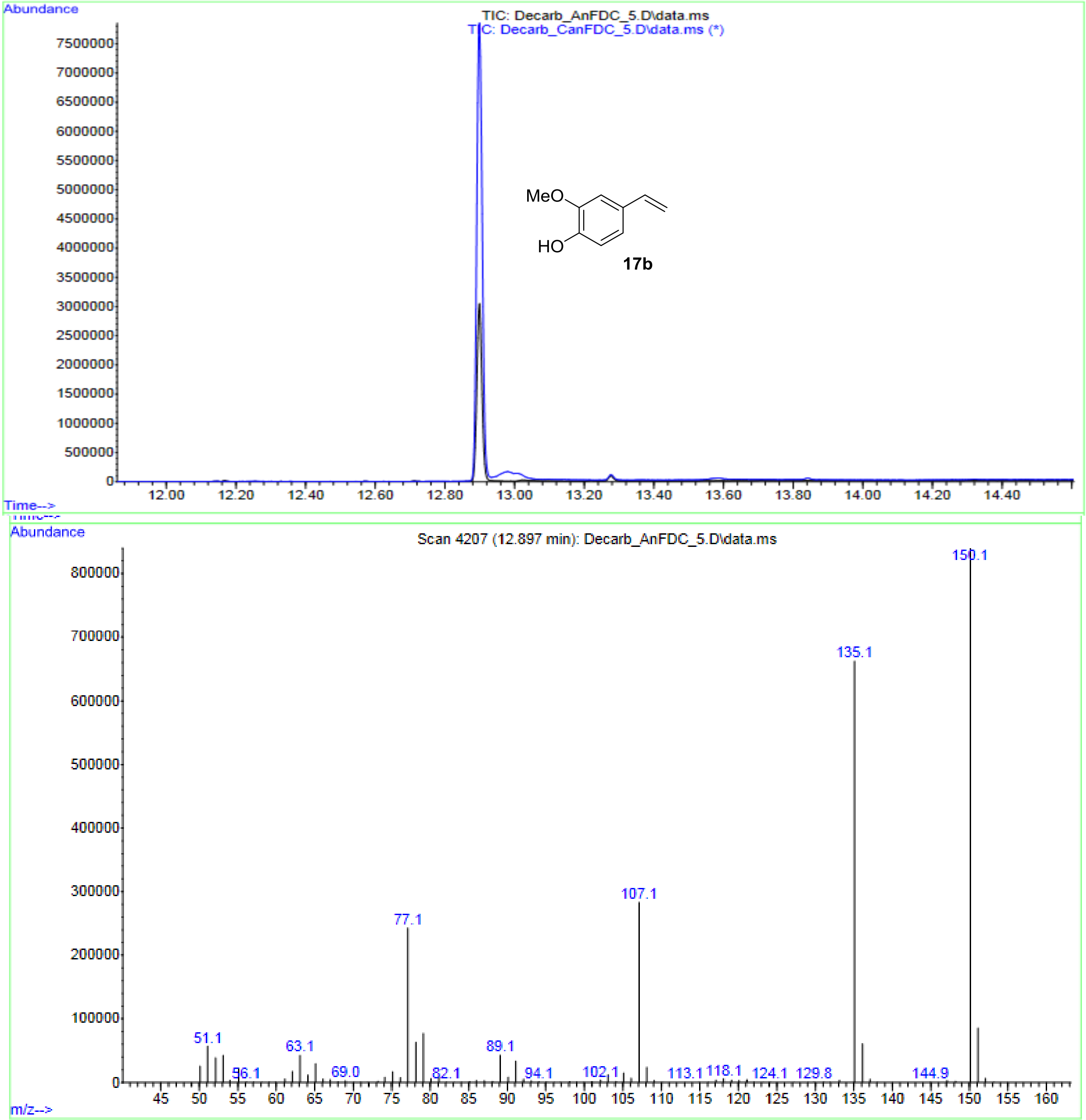

Figure S14. GC-MS-analysis: FDC-catalysed decarboxylation of 20 mM ferulic acid 17a. Row 1: product of *An*FDC^{UbiX}-catalysed reaction (black) overlaid with a trace of *Cd*FDC^{UbiX}-catalysed reaction (blue). Row 2: mass spectrum of product 2-vinylguaicol (17b) of *An*FDC-catalysed biotransformation. MS calcd. for $C_9H_{10}O_2$ (2-vinylguaicol 17b) 150.18, found 150.10.

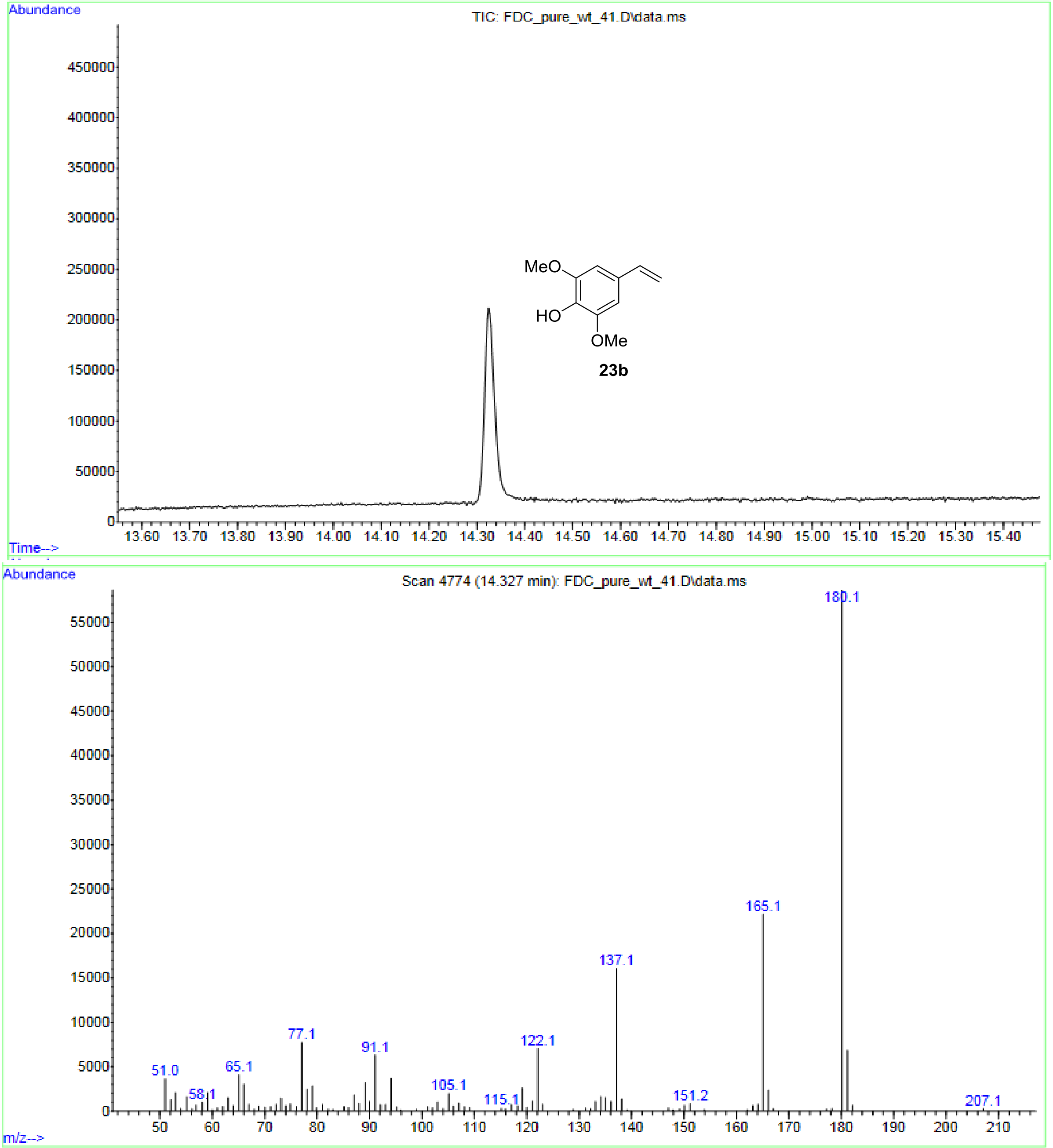

Figure S15. GC-MS-analysis: *An*FDC-catalysed decarboxylation of sinapic acid 23a showing the product of biotransformation (top) and a mass spectrum of product of biotransformation (bottom). MS calcd. for $C_{10}H_{12}O_3$ (23b) 180.08, found 180.10.

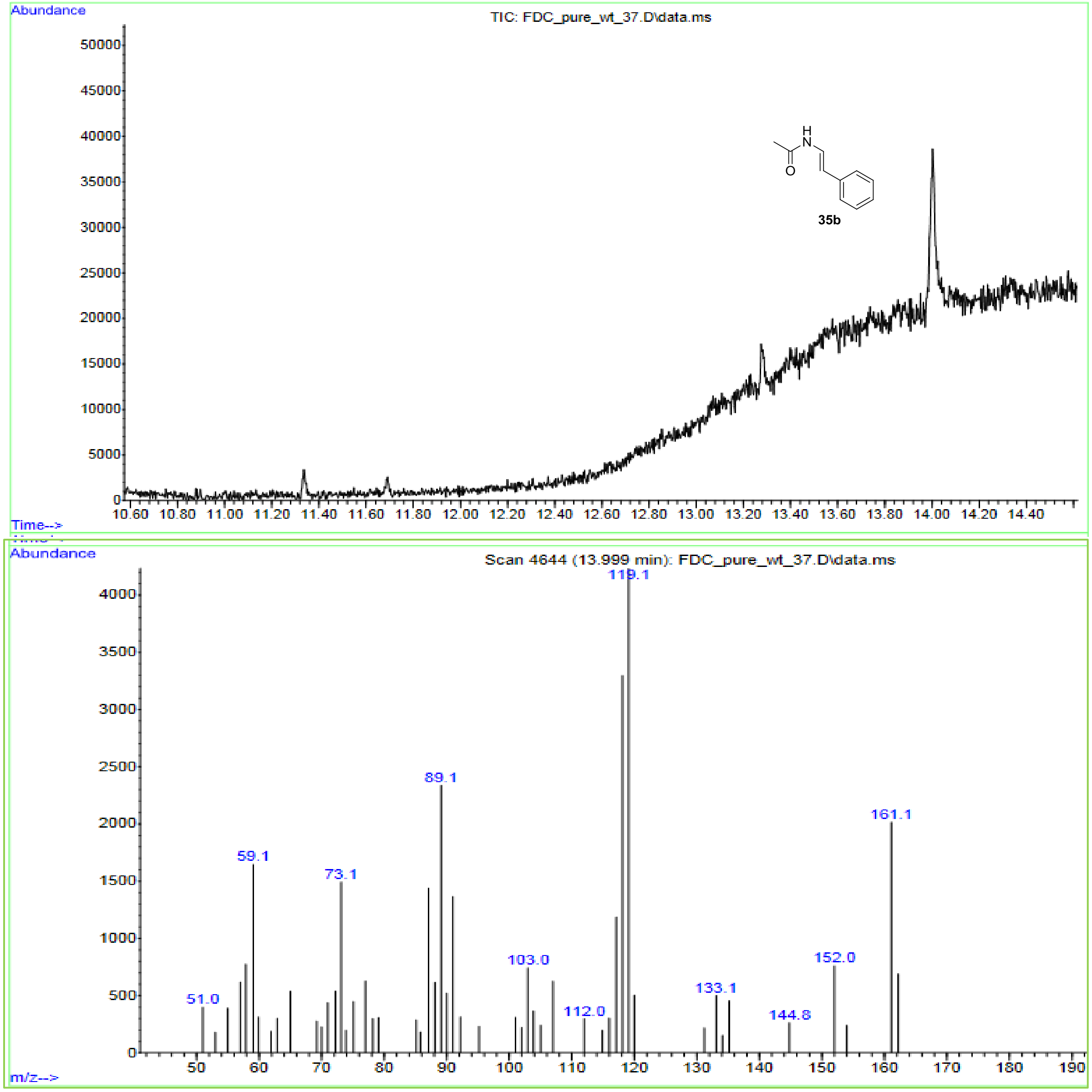

Figure S16. GC-MS-analysis: FDC-catalysed decarboxylation of acetamidocinnamic acid 35a showing the product 35b of *An*FDC^{UbiX}-catalysed reaction (top), mass spectrum of product of *An*FDC-catalysed biotransformation (bottom). MS calcd. for $C_{10}H_{11}NO$ [(*E*)-*N*-styrylacetamide, 63] 161.08, found 161.10.

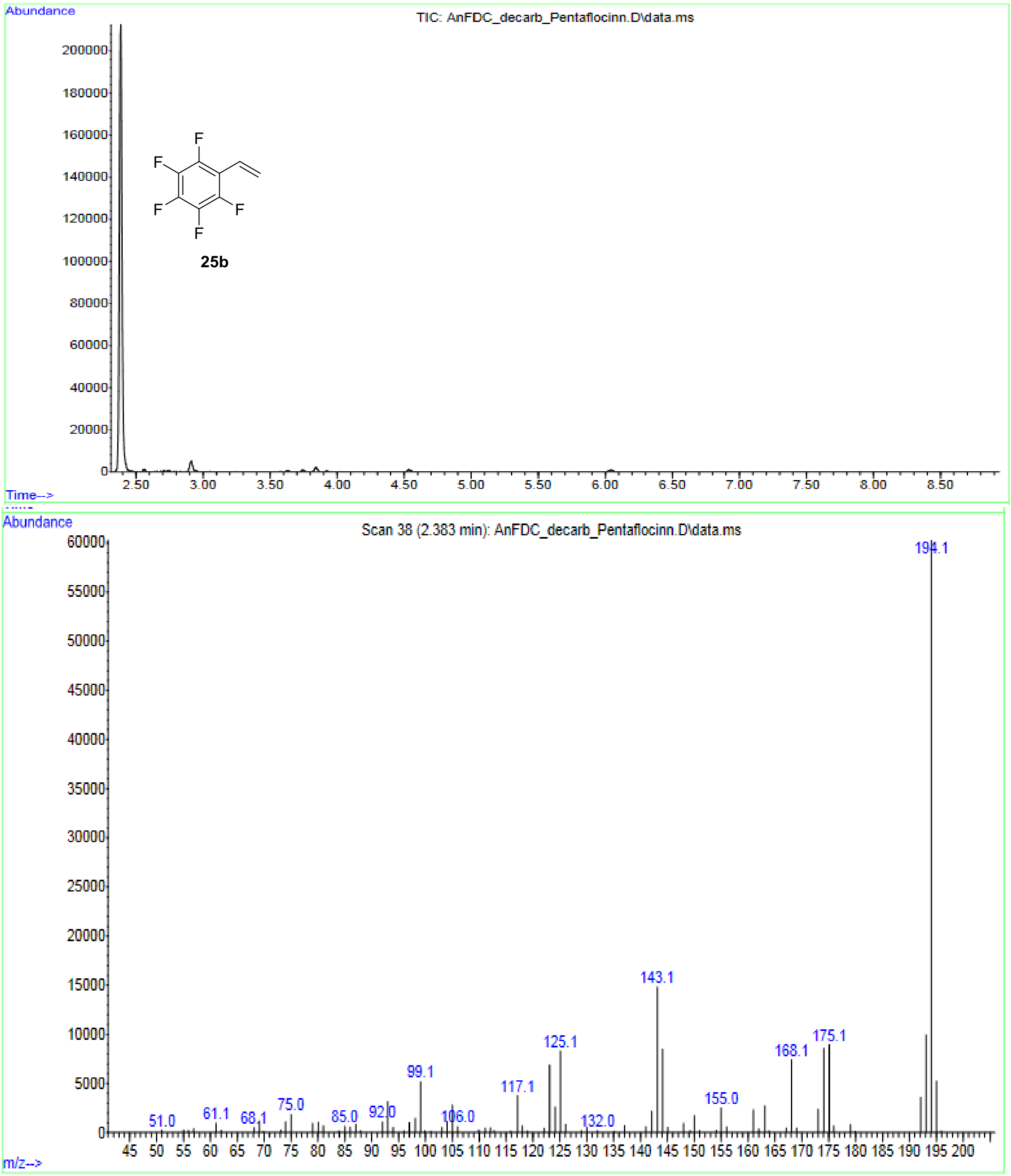

Figure S17. GC-MS-analysis: *An*FDC^{UbiX}-catalysed decarboxylation of pentafluoro cinnamic acid 25a showing the product (top) and a mass spectrum of product of biotransformation (bottom). MS calcd. for $C_8H_3F_5$ (pentafluorostyrene, 25b) 194.02, found 194.20.

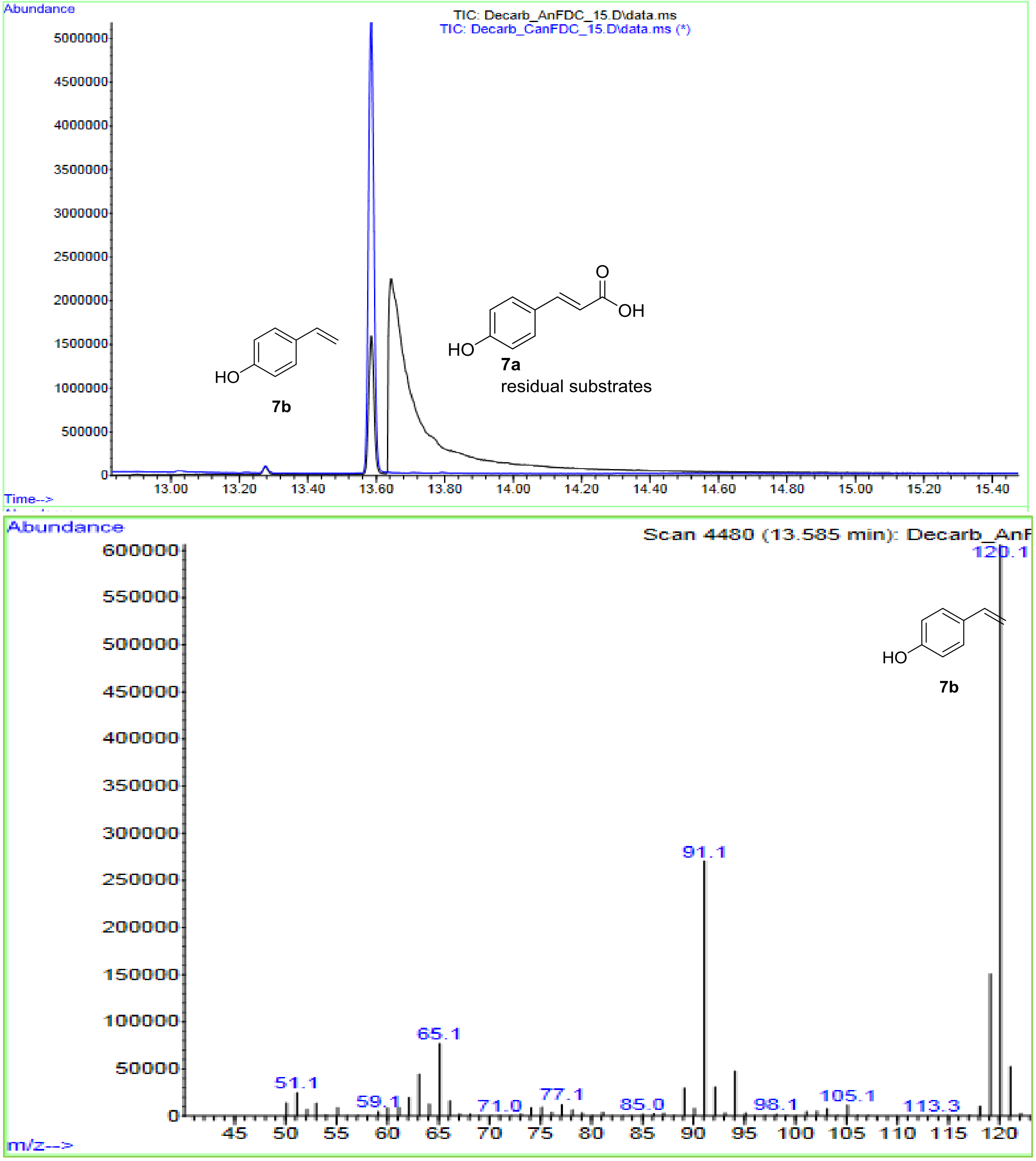

Figure S18. GC-MS-analysis: FDC-catalysed decarboxylation of 20 mM *p*-hydroxy cinnamic acid 7a. Row 1: product of *An*FDC^{UbiX}-catalysed reaction (black) overlaid with a trace of *Cd*FDC^{UbiX}-catalysed reaction (blue). Row 2: mass spectrum of product of *An*FDC-catalysed biotransformation. MS calcd. for C_8H_8O (*p*-hydroxystyrene 7b) 120.06, found 120.10.

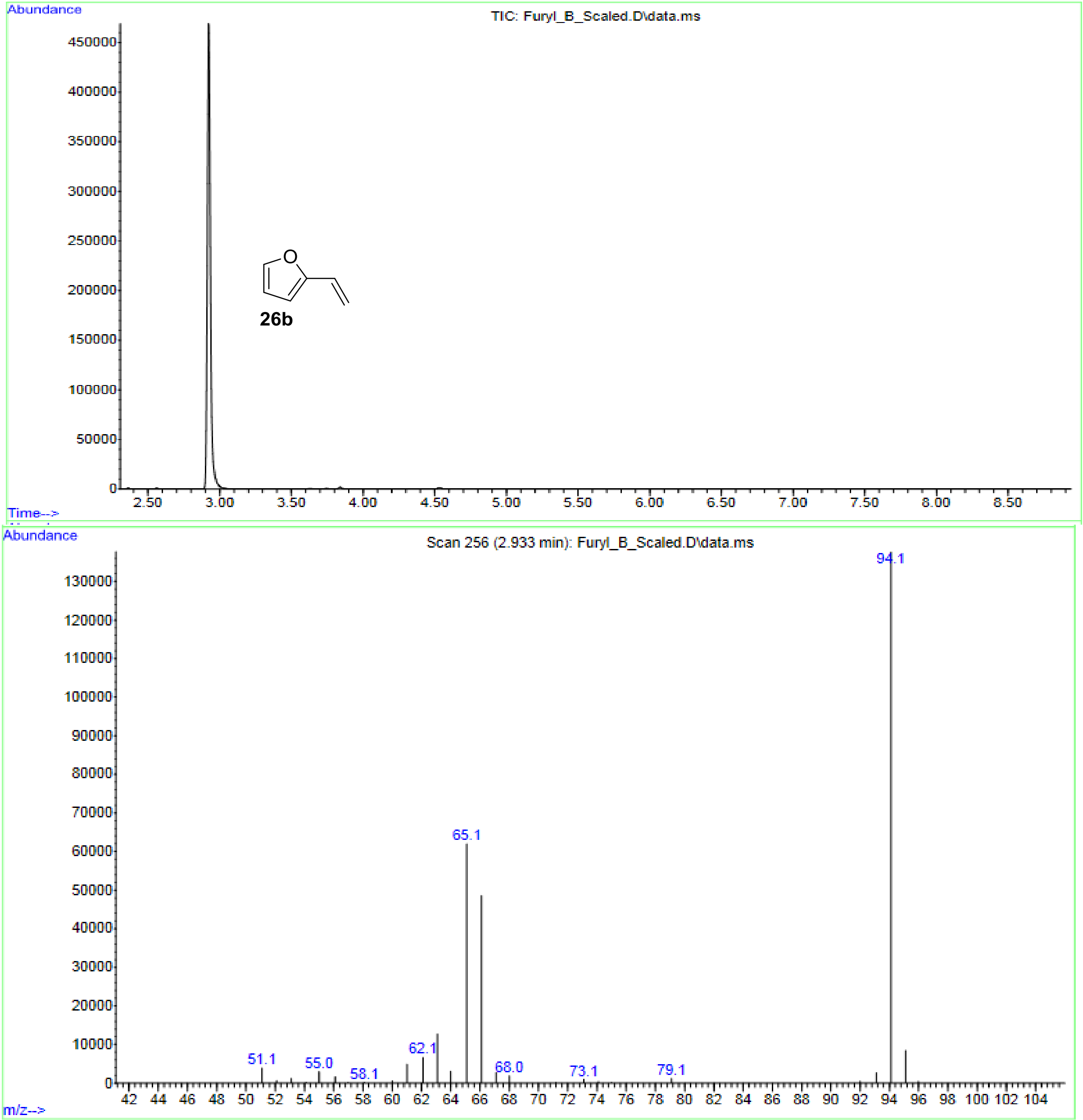

Figure S19. GC-MS-analysis: *An*FDC^{UbiX}-catalysed decarboxylation of 40 mM 3-(2-furyl)acrylic acid 26a showing the product (top), mass spectrum of product of biotransformation (bottom). MS calcd. for C_6H_6O (2-vinylfuran 26b) 94.04, found 94.10.

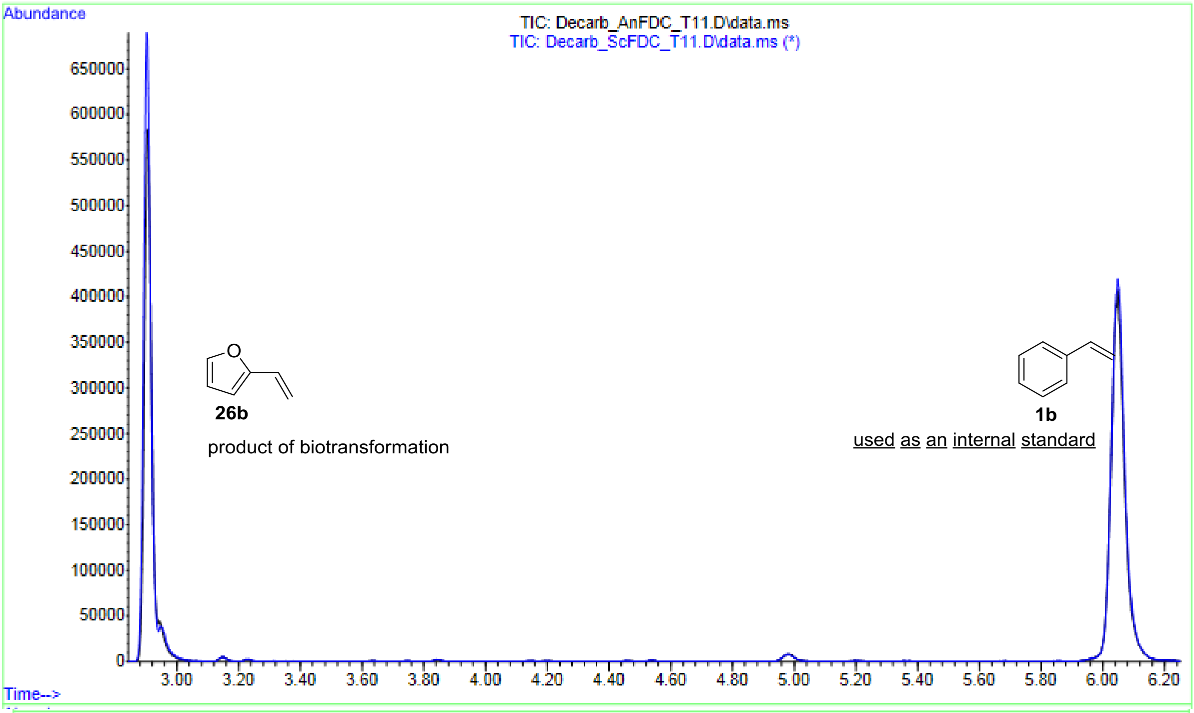

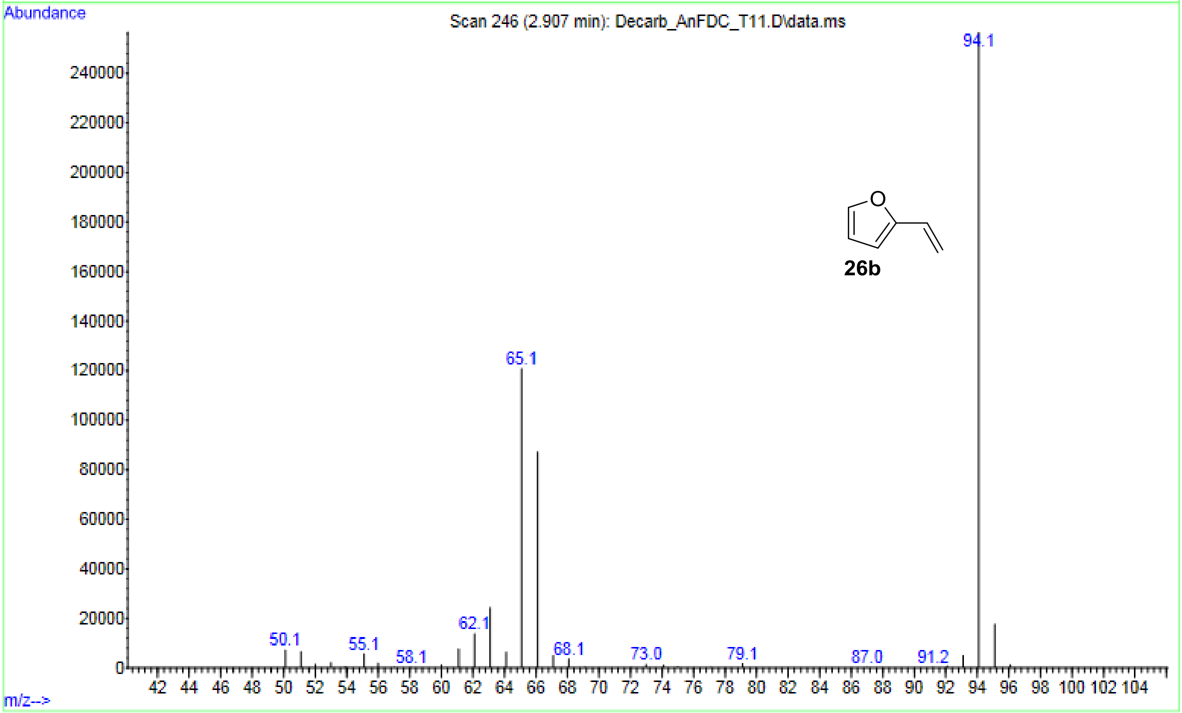

Figure S20. GC-MS-analysis: FDC-catalysed decarboxylation of 40 mM 3-(2-furyl)acrylic acid 26a showing the product of *An*FDC^{UbiX}-catalysed reaction (top), mass spectrum of product of *An*FDC-catalysed biotransformation (bottom). MS calcd. for C_6H_6O (2-vinylfuran 26b) 94.04, found 94.10.

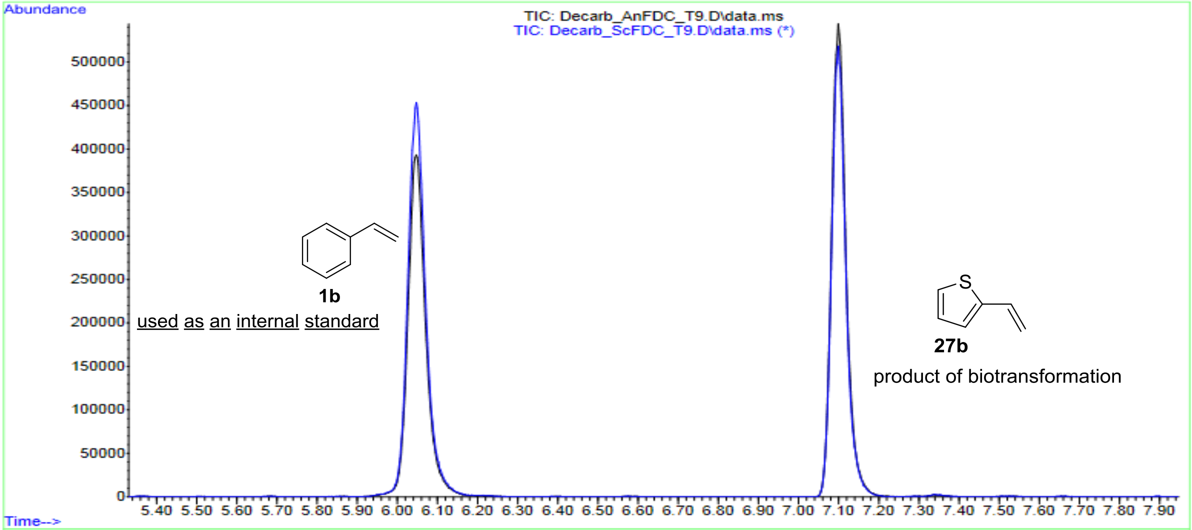

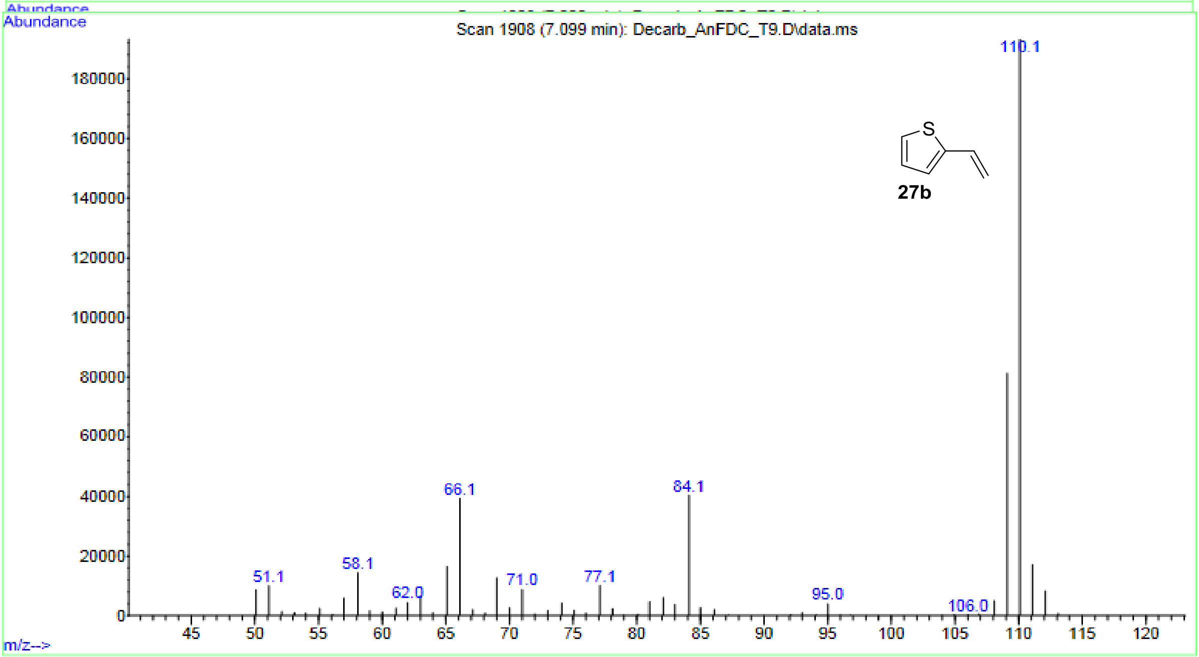

Figure S21. GC-MS-analysis: FDC-catalysed decarboxylation of 40 mM 3-(2-thienyl)acrylic acid 27a. Row 1: product of *An*FDC^{UbiX}-catalysed reaction (black) overlaid with a trace of *Sc*FDC^{UbiX}-catalysed reaction (blue), with styrene used as internal standard. Row 2: mass spectrum of product of *An*FDC-catalysed biotransformation. MS calcd. for C_6H_6S (2-vinylthiophene 27b) 110.02, found 110.10.

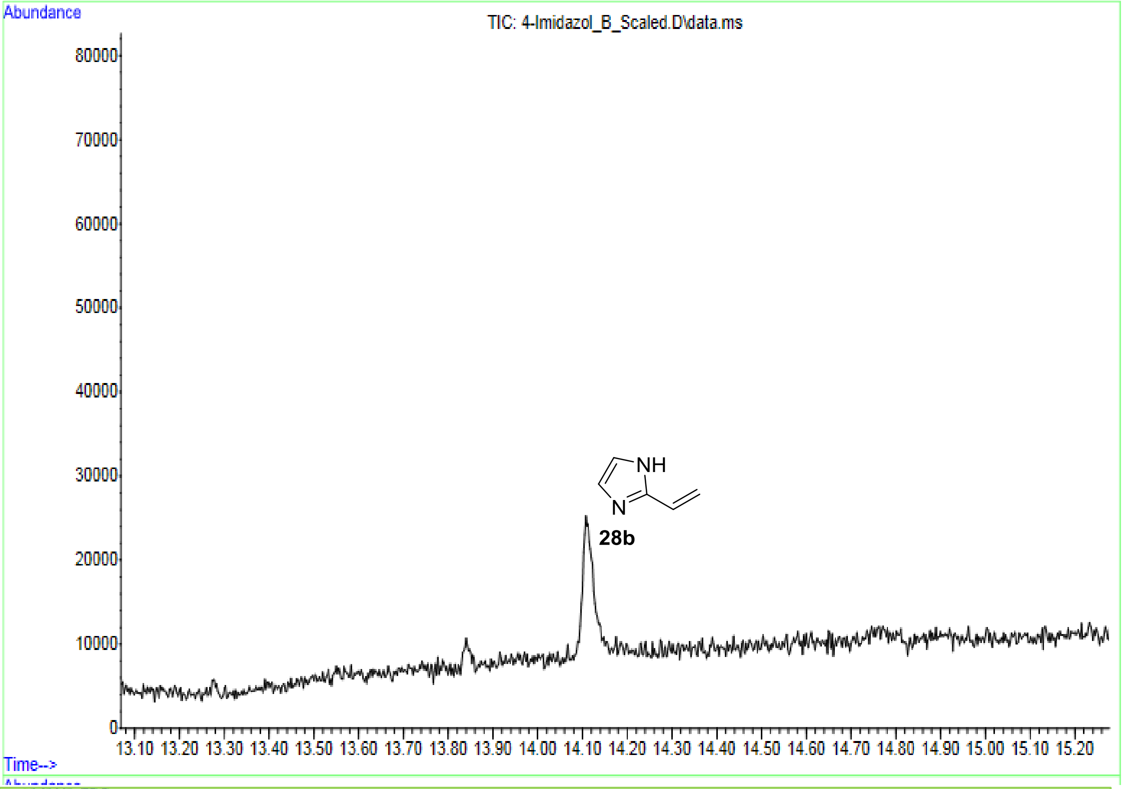

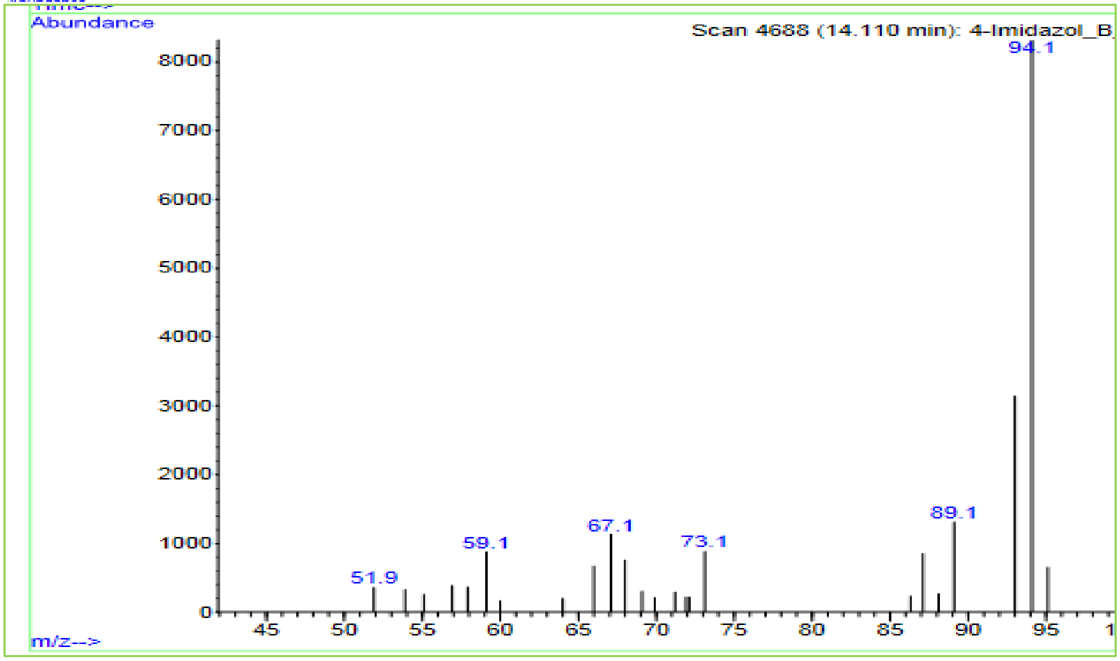

Figure S22. GC-MS-analysis: FDC-catalysed decarboxylation of 4-imidazolyl acrylic acid 28a. Product of *An*FDC^{UbiX}-catalysed reaction (top), mass spectrum of product of *An*FDC-catalysed biotransformation (bottom). MS calcd. for $C_5H_6N_2$ (2-vinyl-1*H*-imidazole 28b) 94.05, found 94.10.

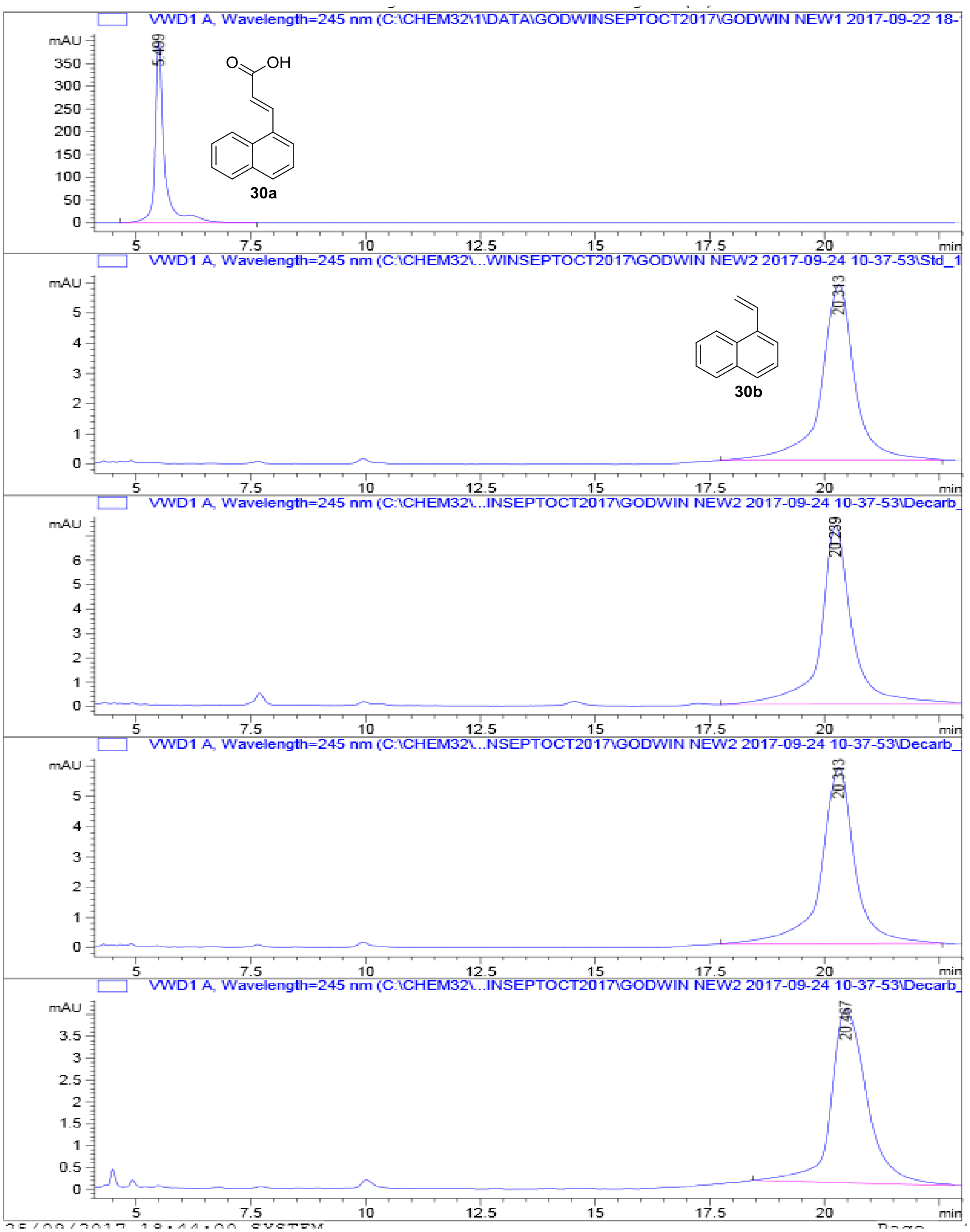

Figure S23. HPLC analysis: FDC-catalysed decarboxylation of 3-(1-naphthyl)acrylic acid 30a. Standards of starting material 30a (row 1), product 1-vinylnapthalene 30b (row 2) and reactions catalysed by *An*FDC^{UbiX} and *Sc*FDC^{UbiX} and *Cd*FDC^{UbiX}, respectively (rows 3-5).

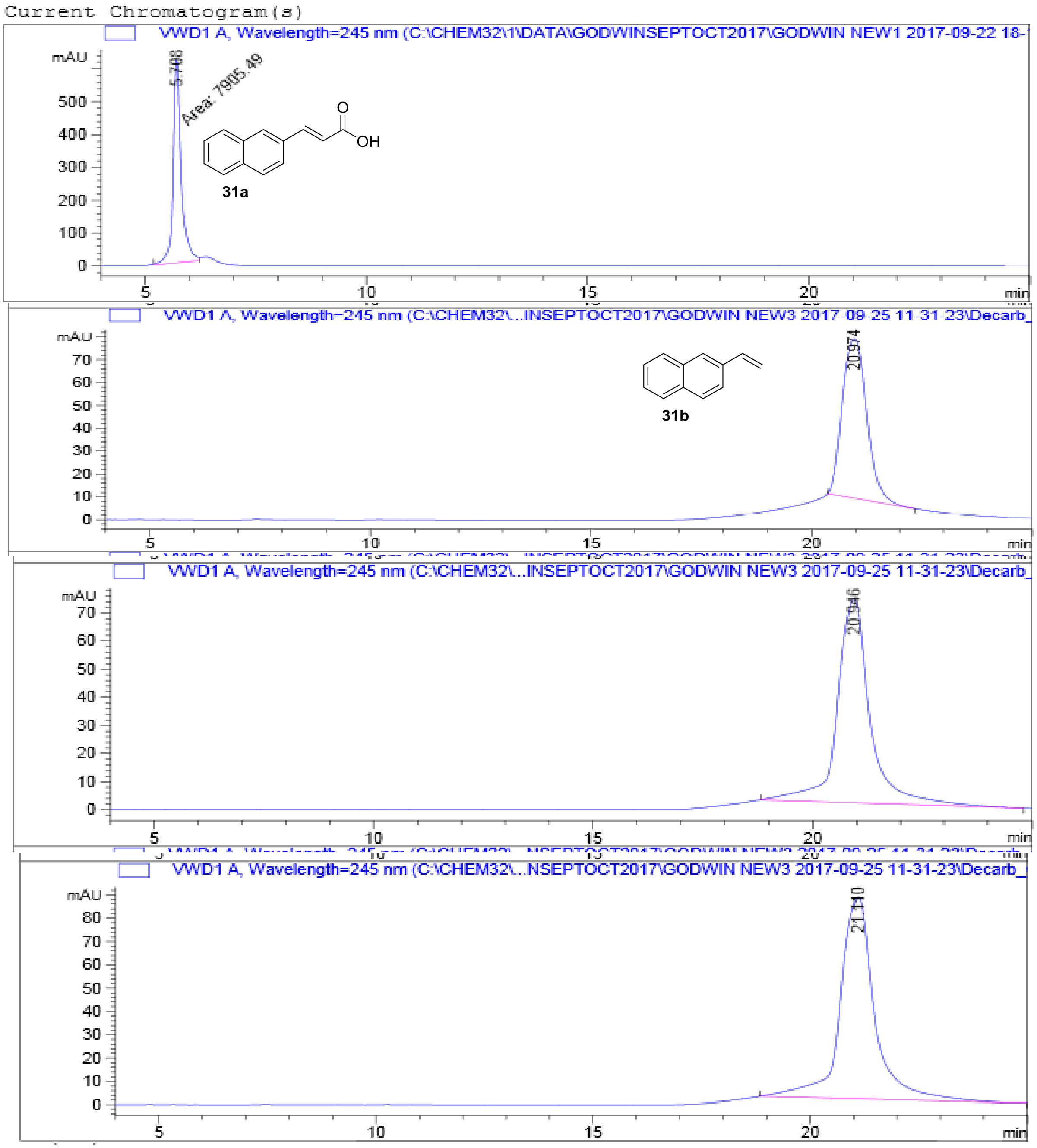

Figure S24. HPLC analysis: FDC-catalysed decarboxylation of 3-(2-naphthyl)acrylic acid 31a. Standards of starting material 31a (row 1), product 2-vinylnapthalene 31b (row 2) and reactions catalysed by *An*FDC^{UbiX} and *Sc*FDC^{UbiX}, respectively (rows 3 & 4).

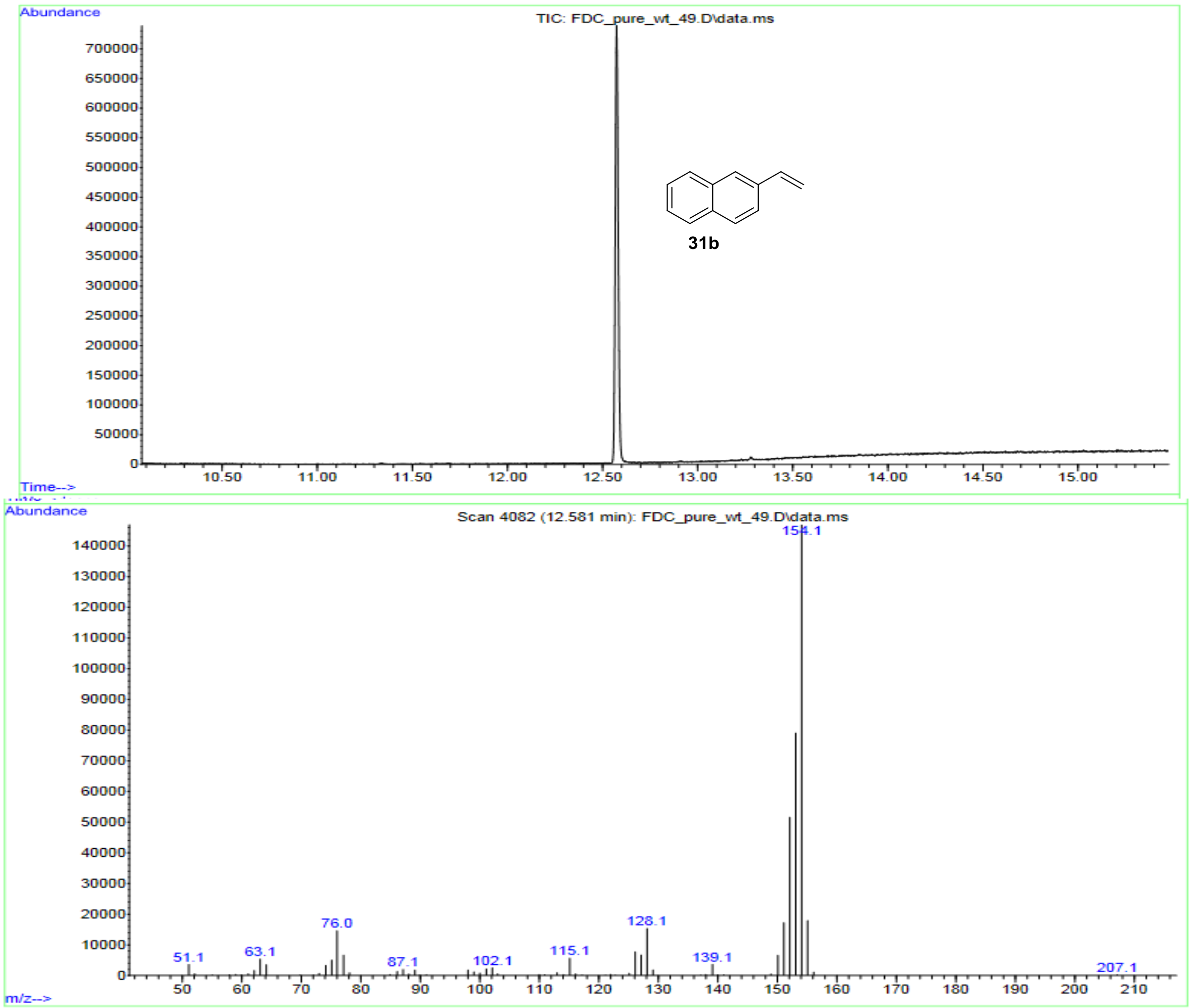

Figure S25. GC-MS-analysis: FDC-catalysed decarboxylation of 30 mM 3-(2-naphthyl)acrylic acid 31a. Product of *An*FDC^{UbiX}-catalysed reaction (top), mass spectrum of product of *An*FDC-catalysed biotransformation (bottom). MS calcd. for $C_{12}H_{10}$ (2-vinylnapthalene 31b) 154.08, found 154.10.

Section S7. Synthesis of reference material

Section S7.1. General procedure

1.1 eq. of methyltriphenylphosphonium bromide was added to a heat-dried two-neck flask (stored at 80 °C in a drying oven overnight) and argon was flushed through the vessel for several minutes. 10 mL of distilled THF was added and 1.2 eq. of lithium bis(trimethylsilylamide) solution (1 M in THF) was added under stirring. As soon as the solids were dissolved, 1.0 eq. aldehyde (see below) was added to the stirred ylide solution. The reaction was monitored by TLC control (Merck silica gel 60) until completion. The mixture was quenched with mild acid, dried over Na_2SO_4 and evaporated, followed by flash chromatography to yield the styrene derivatives.

Section S7.2. 4-Hydroxy-3,5-dimethoxystyrene 23b

Syringaldehyde (703 mg, 3.9 mmol, 1.0 eq.); yielding 105 mg of product as colorless oil (0.6 mmol, 15% yield, $R_f \approx 0.77$).

^{1}H NMR (300 MHz, $CDCl_3$): δ = 6.71 – 6.52 (m, 3H), 5.62 (dd, *J* = 17.5, 0.8 Hz, 1H), 5.16 (dd, *J* = 10.8, 0.7 Hz, 1H), 3.83 (s, 6H); ^{13}C NMR (75 MHz, $CDCl_3$): δ = 151.74, 137.09, 134.36, 130.67, 112.21, 103.53, 56.03.[1]

Section S7.3. 2,5-Dimethoxystyrene 21b

2,5-Dimethoxybenzaldehyde (610 mg, 3.7 mmol, 1.0 eq.); yielding 426 mg of product as colorless oil (2.6 mmol, 70% yield, $R_f \approx 0.84$).

^{1}H NMR (300 MHz, $CDCl_3$): δ = 7.16 – 6.93 (m, 2H), 6.89 – 6.71 (m, 2H), 5.74 (dd, *J* = 17.7, 1.5 Hz, 1H), 5.37 – 5.22 (m, 1H), 3.81 (d, *J* = 2.0 Hz, 3H), 3.80 (s, 3H); ^{13}C NMR (75 MHz, $CDCl_3$): δ = 153.80, 151.32, 131.62, 127.70, 114.81, 113.89, 112.35, 111.97, 56.34, 55.85.[2]

Section S7.4. 2,3-Dimethoxystyrene 22b

2,3-Dimethoxybenzaldehyde (619 mg, 3.7 mmol, 1.0 eq.); yielding 356 mg of product as colorless oil (2.2 mmol, 59% yield, $R_f \approx 0.82$).

^{1}H NMR (300 MHz, $CDCl_3$): δ = 7.18 – 6.98 (m, 3H), 6.83 (dd, *J* = 8.0, 1.5 Hz, 1H), 5.76 (dd, *J* = 17.8, 1.4 Hz, 1H), 5.31 (dd, *J* = 11.1, 1.4 Hz, 1H), 3.87 (s, 3H), 3.81 (s, 3H); ^{13}C NMR (75 MHz, $CDCl_3$): δ = 153.11, 146.82, 131.92, 131.28, 124.12, 117.92, 115.30, 111.64, 61.04, 55.93.[3]

Section S7.5. 2-Vinylthiophene 27b

Thiophene-2-carboxaldehyde (330 µL, ρ=1.223 g mL^{-1}, 3.6 mmol, 1.0 eq.); yielding 131 mg of product as yellow oil (1.2 mmol, 33% yield, $R_f \approx 0.94$).

^1H NMR (300 MHz, $CDCl_3$): δ = 7.24 – 7.14 (m, 1H), 7.04 – 6.93 (m, *J* = 5.4, 2.6 Hz, 2H), 6.83 (dd, *J* = 17.3, 10.8 Hz, 1H), 5.59 (d, *J* = 17.3 Hz, 1H), 5.16 (d, *J* = 10.8 Hz, 1H); ^{13}C NMR (75 MHz, $CDCl_3$): δ = 143.17, 129.98, 127.42, 125.91, 124.43, 113.36.[4]

Section S7.6. 2-Vinylfuran 26b

Furfural (330 μL, ρ=1.160 g mL^{-1}, 3.6 mmol, 1.0 eq.); yielding 61 mg of product as yellow oil (0.6 mmol, 18% yield, $R_f \approx 0.72$). Due to the instability of the product, no NMR spectra or HPLC chromatograms could be obtained.

Section S7.7. 3,4-Dihydroxystyrene 18b

3,4-Dihydroxybenzaldehyde (542 mg, 3.9 mmol, 1.0 eq.); giving 115 mg of product as colorless oil (0.8 mmol, 22% yield, $R_f \approx 0.88$).

^1H NMR (300 MHz, $CDCl_3$): δ = 6.98 – 6.88 (m, 2H), 6.80 (dd, *J* = 7.2, 1.3 Hz, 1H), 6.62 (dd, *J* = 17.6, 10.8 Hz, 1H), 5.59 (dd, *J* = 17.6, 0.9 Hz, 1H), 5.14 (dd, *J* = 10.8, 0.9 Hz, 1H); ^{13}C NMR (75 MHz, $CDCl_3$): δ = 146.67 (d), 136.49, 131.91, 125.66, 121.03, 120.25, 118.83, 112.01.[5]

Section S8. Spectra

Section S8.1. 2-Methoxy-4-vinylphenol 17b[6]

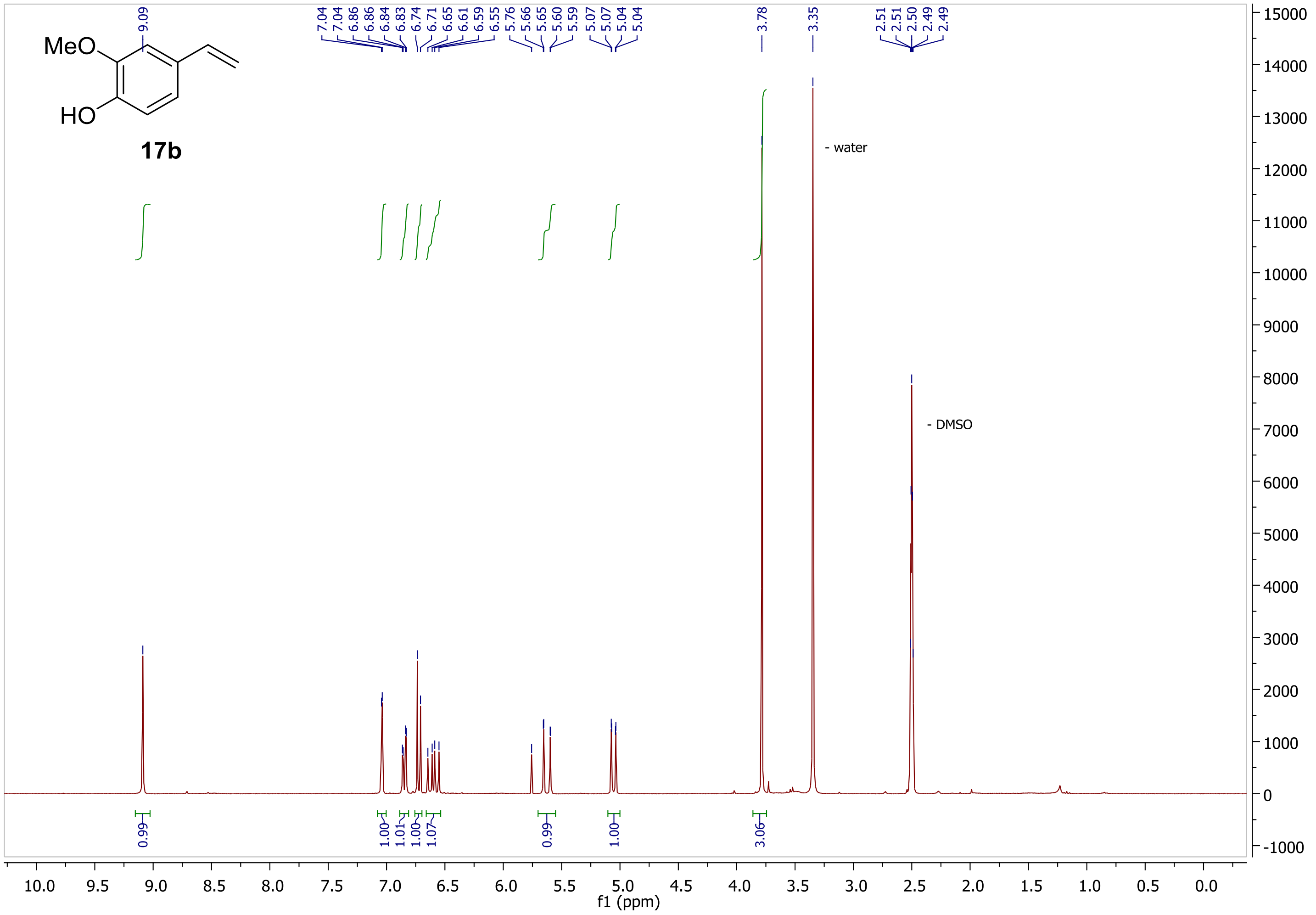

Figure S26. ^{1}H NMR spectrum of 17b (300 MHz, DMSO-d_6).

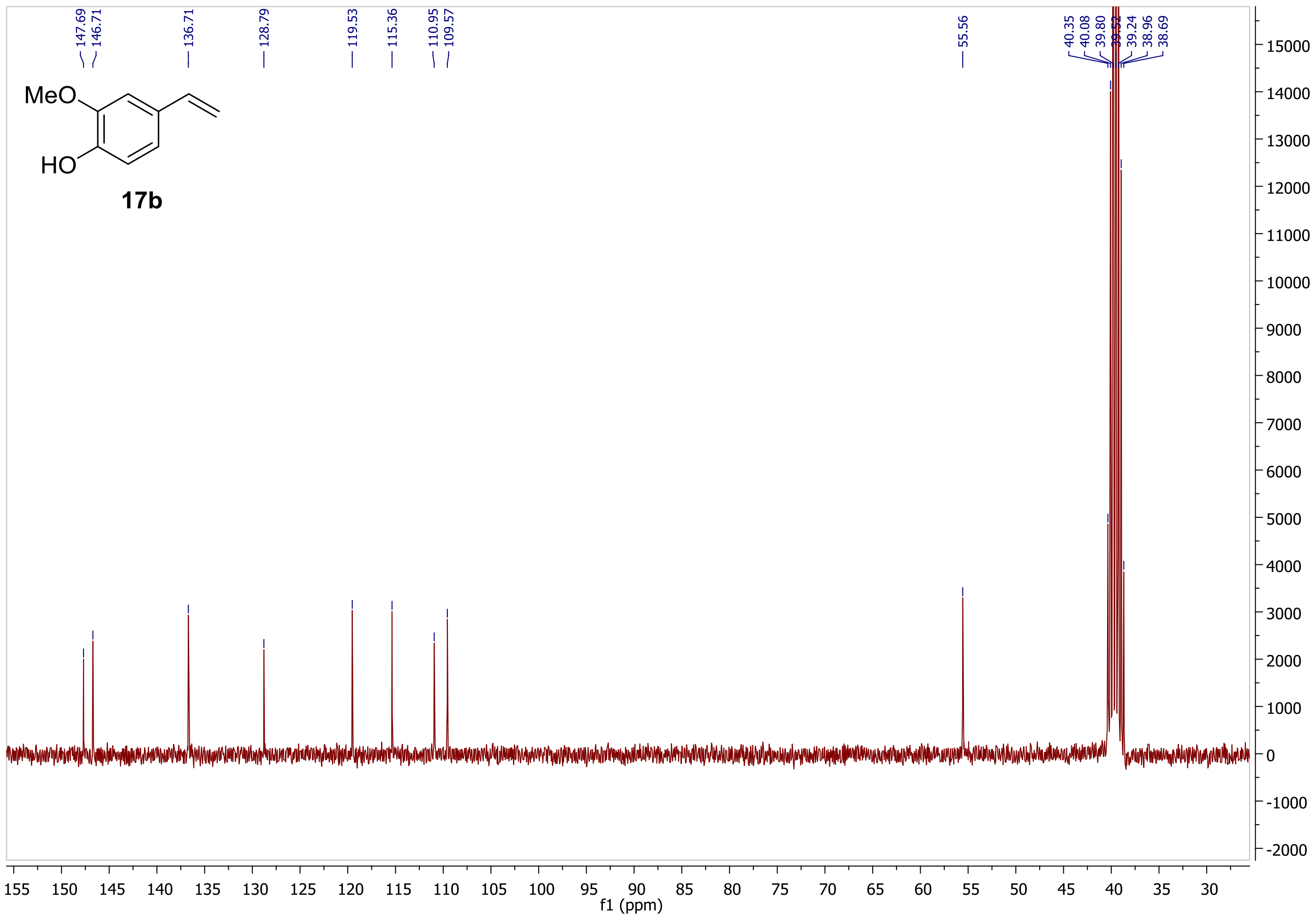

Figure S27. ^{13}C NMR spectrum of 17b (75 MHz, DMSO-d_6).

Section S8.2. 4-Hydroxy-3,5-dimethoxystyrene 23b[1]

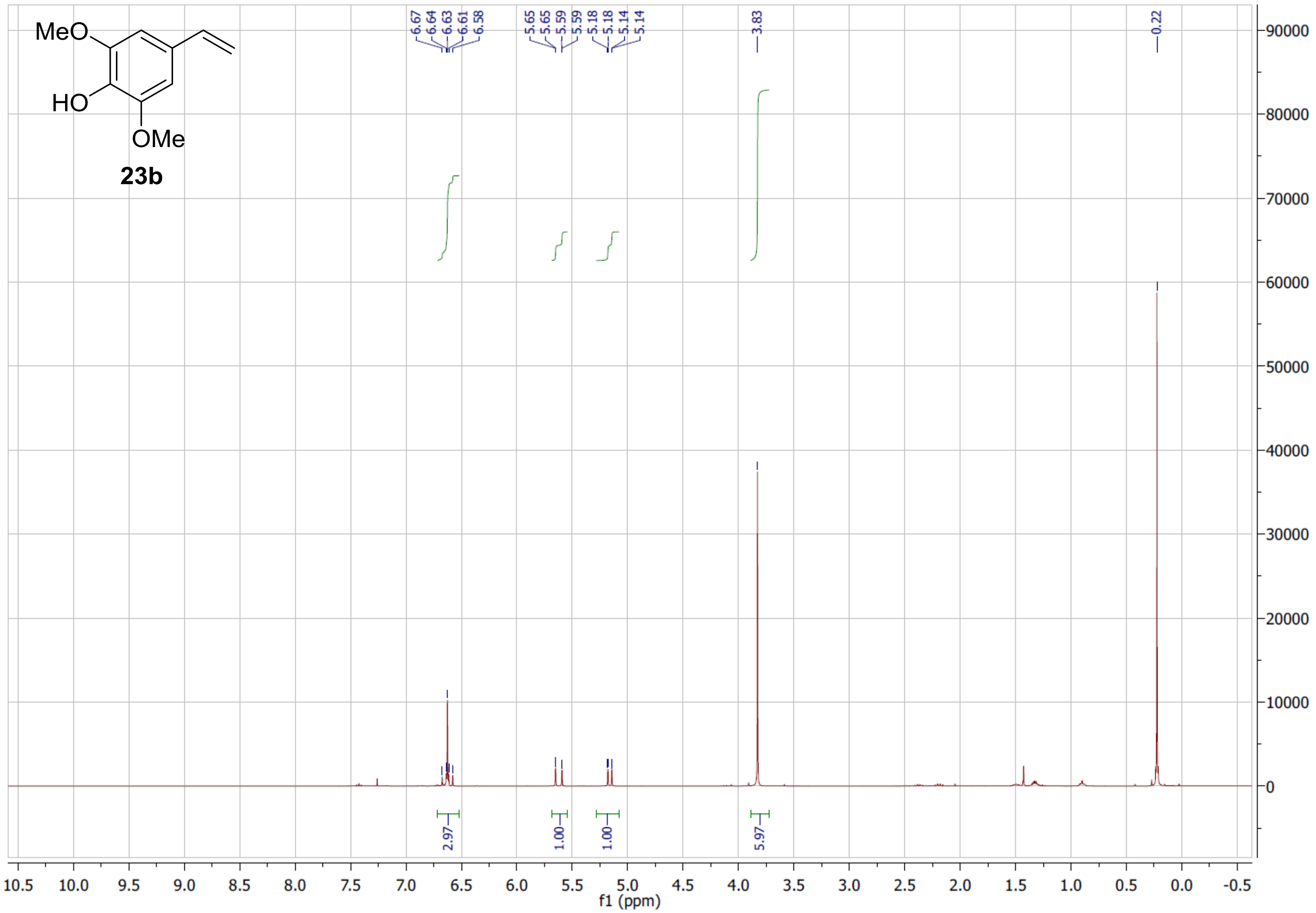

Figure S28. ^{1}H NMR spectrum of 23b (300 MHz, $CDCl_3$).

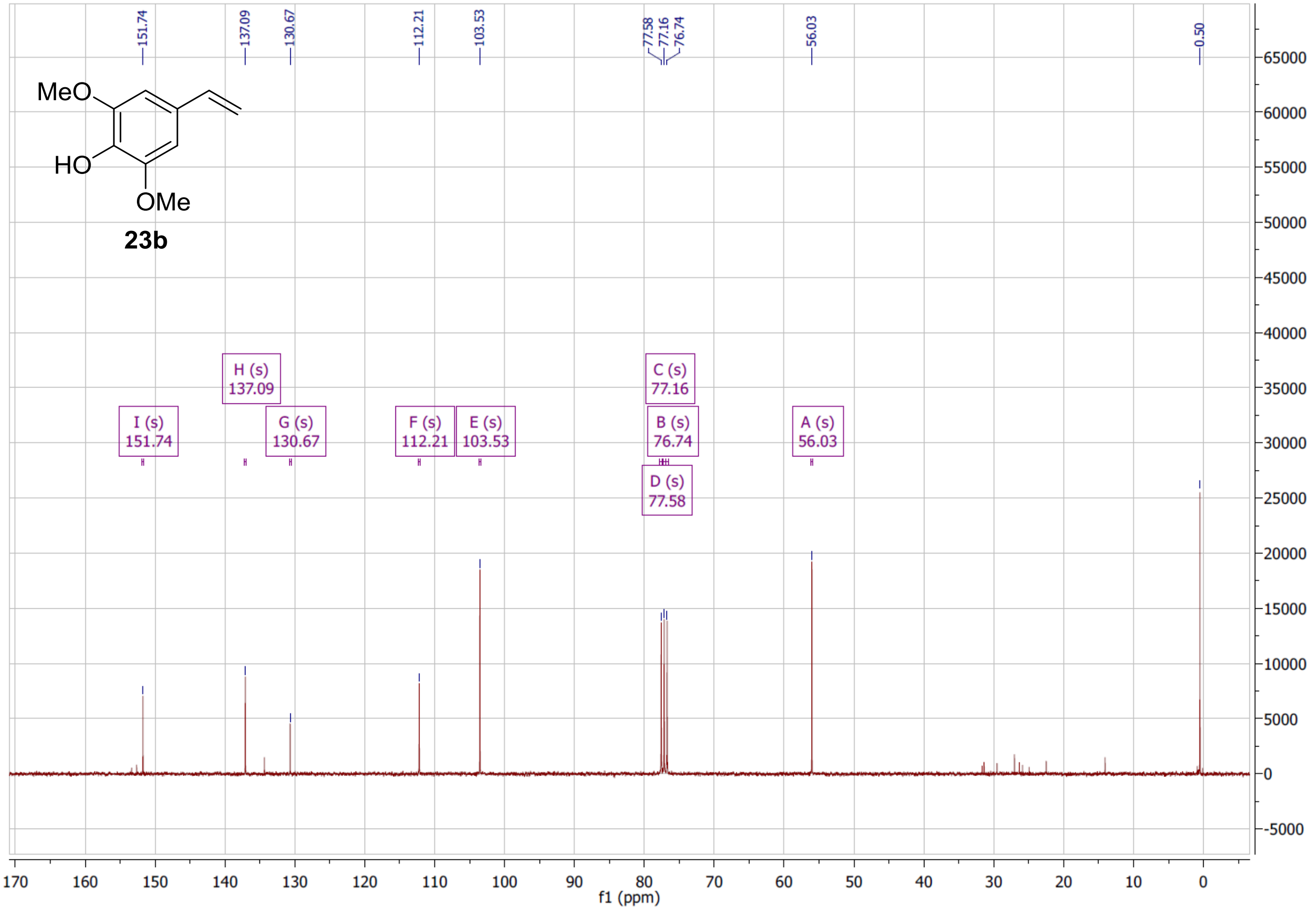

Figure S29. ^{13}C NMR spectrum of 23b (75 MHz, $CDCl_3$).

Section S8.3. 2,5-Dimethoxystyrene 21b[2]

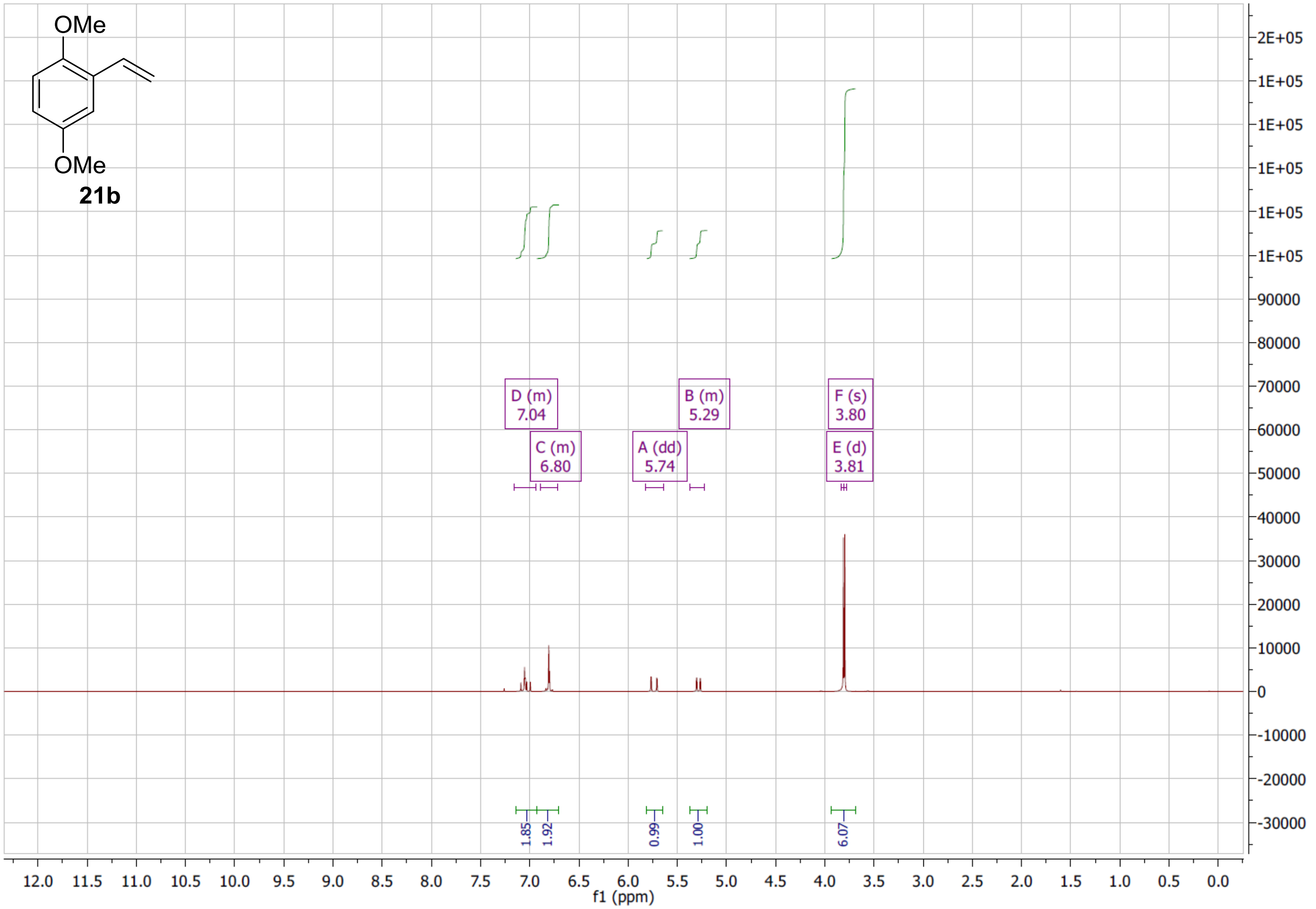

Figure S30. ^{1}H NMR spectrum of 21b (300 MHz, $CDCl_3$).

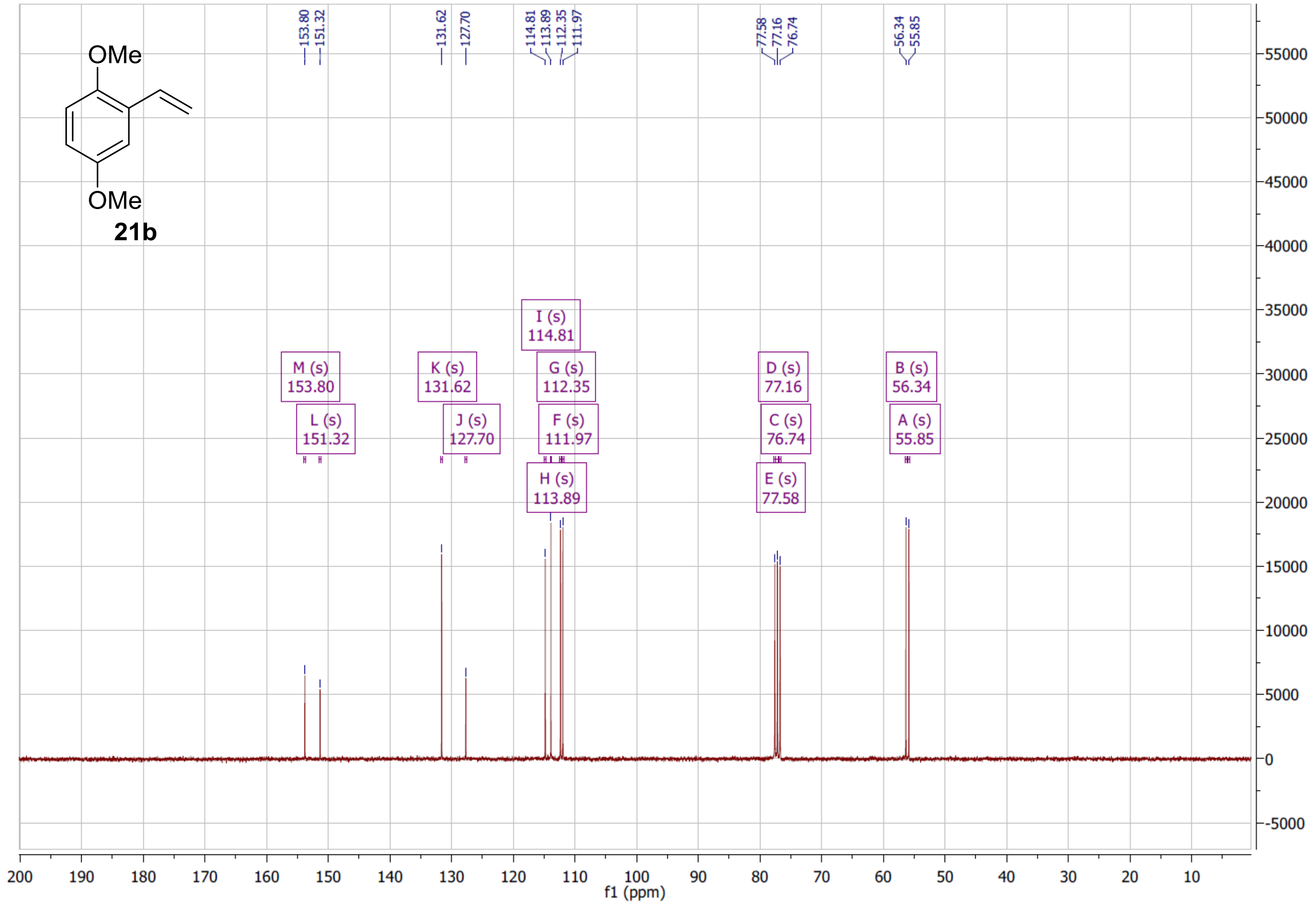

Figure S31. ^{13}C NMR spectrum of 21b (75 MHz, $CDCl_3$).

Section S8.4. 2,3-Dimethoxystyrene 22b[3]

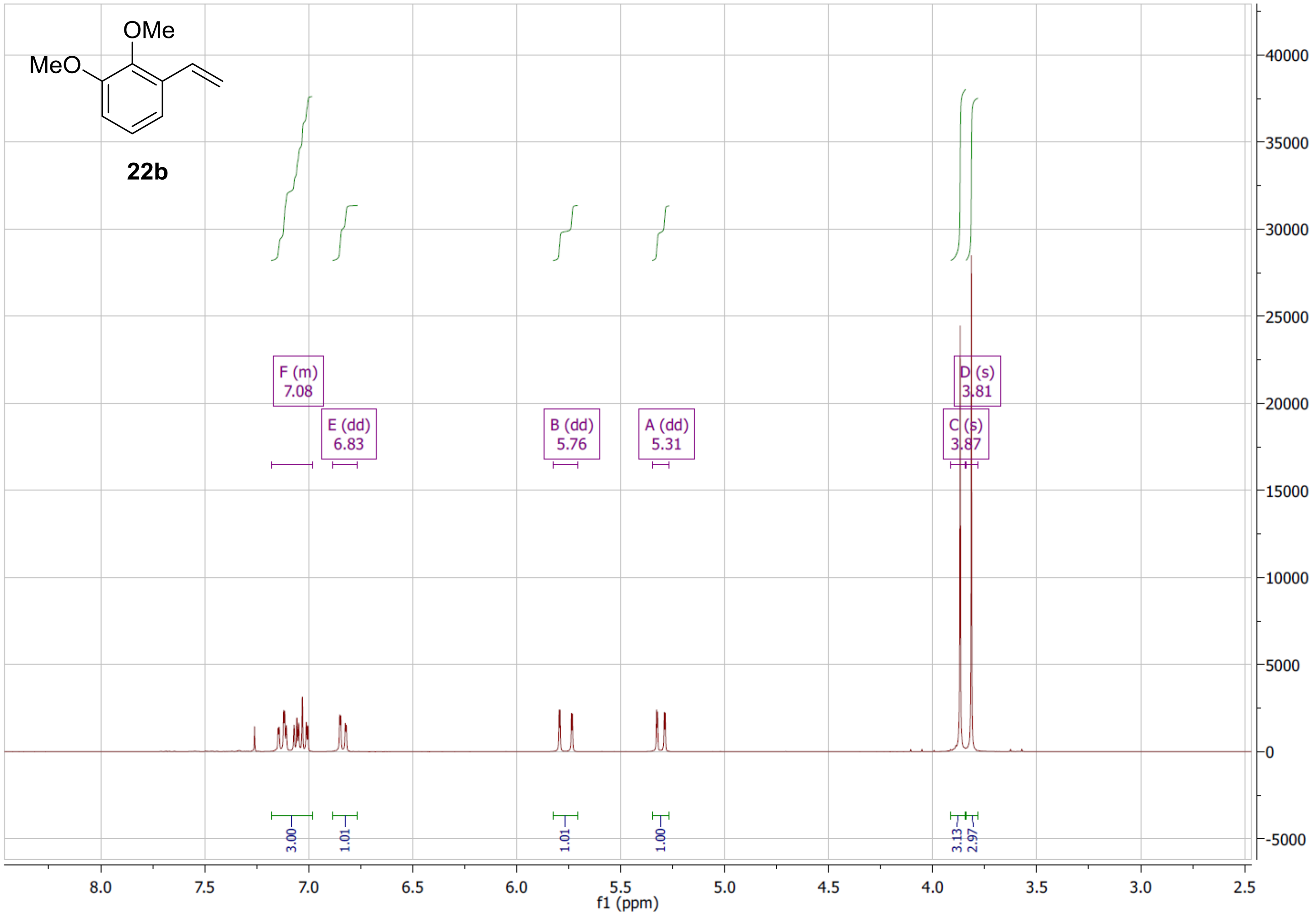

Figure S32. ^{1}H NMR spectrum of 22b (300 MHz, $CDCl_3$).

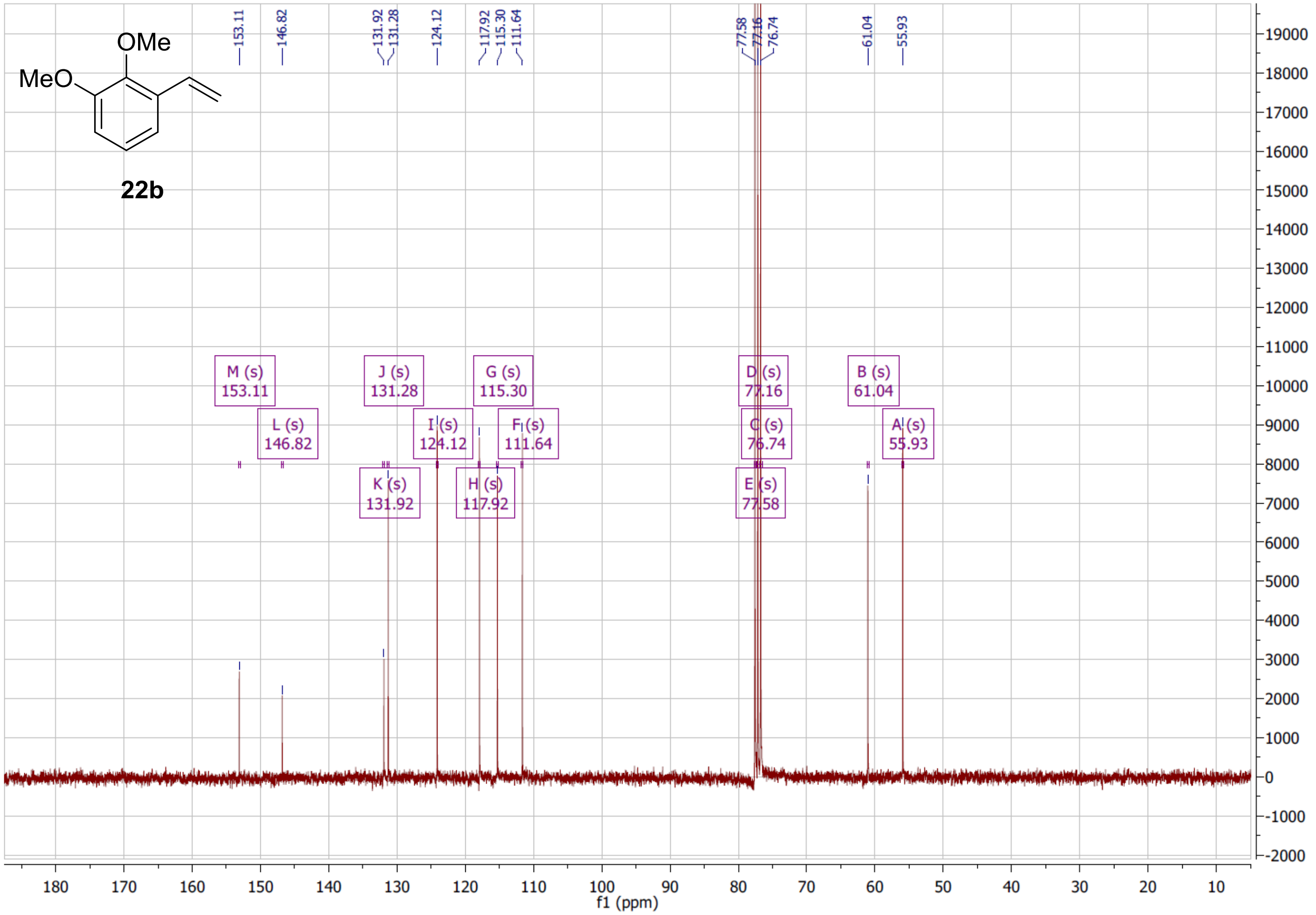

Figure S33. ^{13}C NMR spectrum of 22b (75 MHz, $CDCl_3$).

Section S8.5. 2-Vinylthiophen 27b[4]

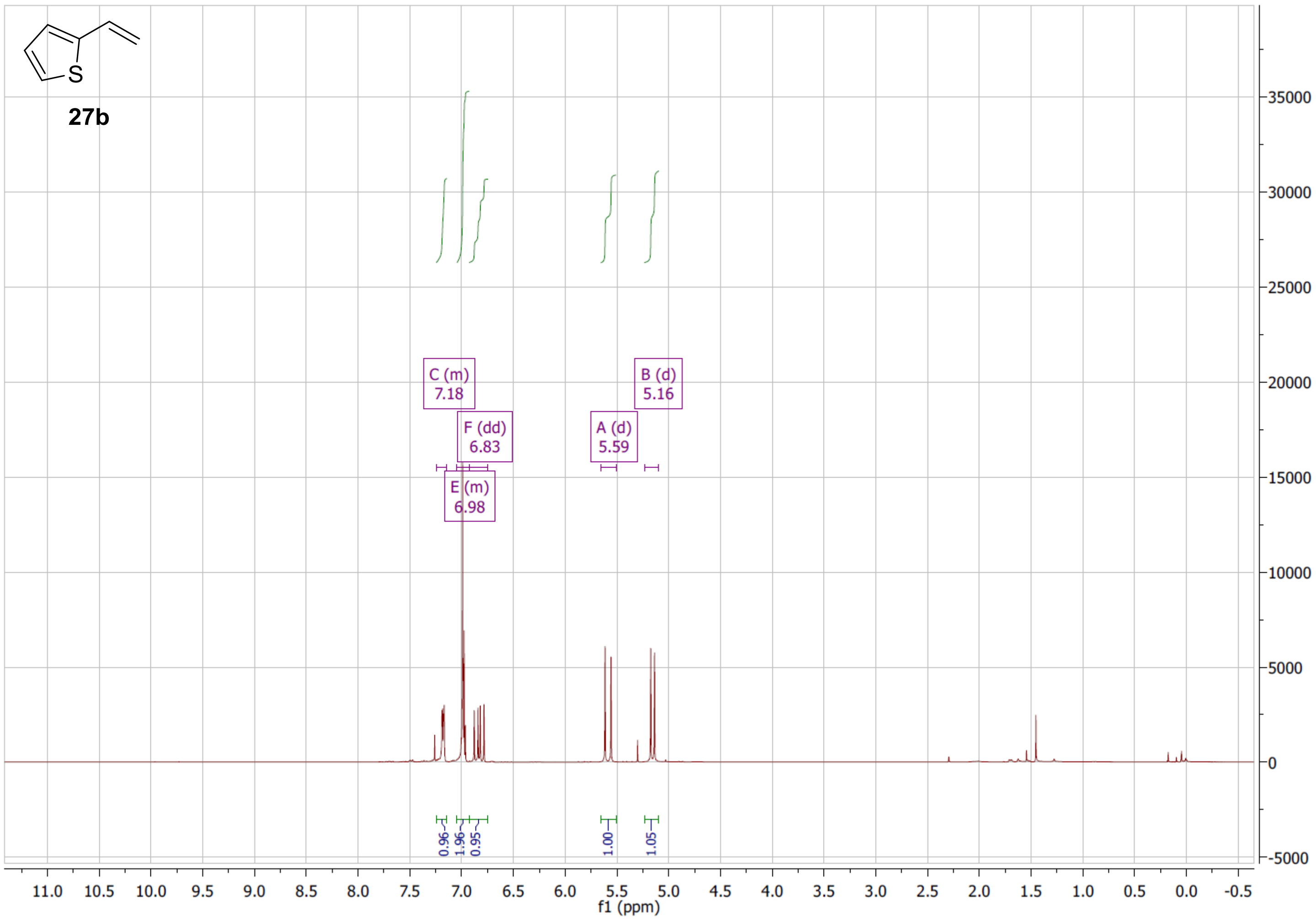

Figure S34. ^{1}H NMR spectrum of 27b (300 MHz, $CDCl_3$).

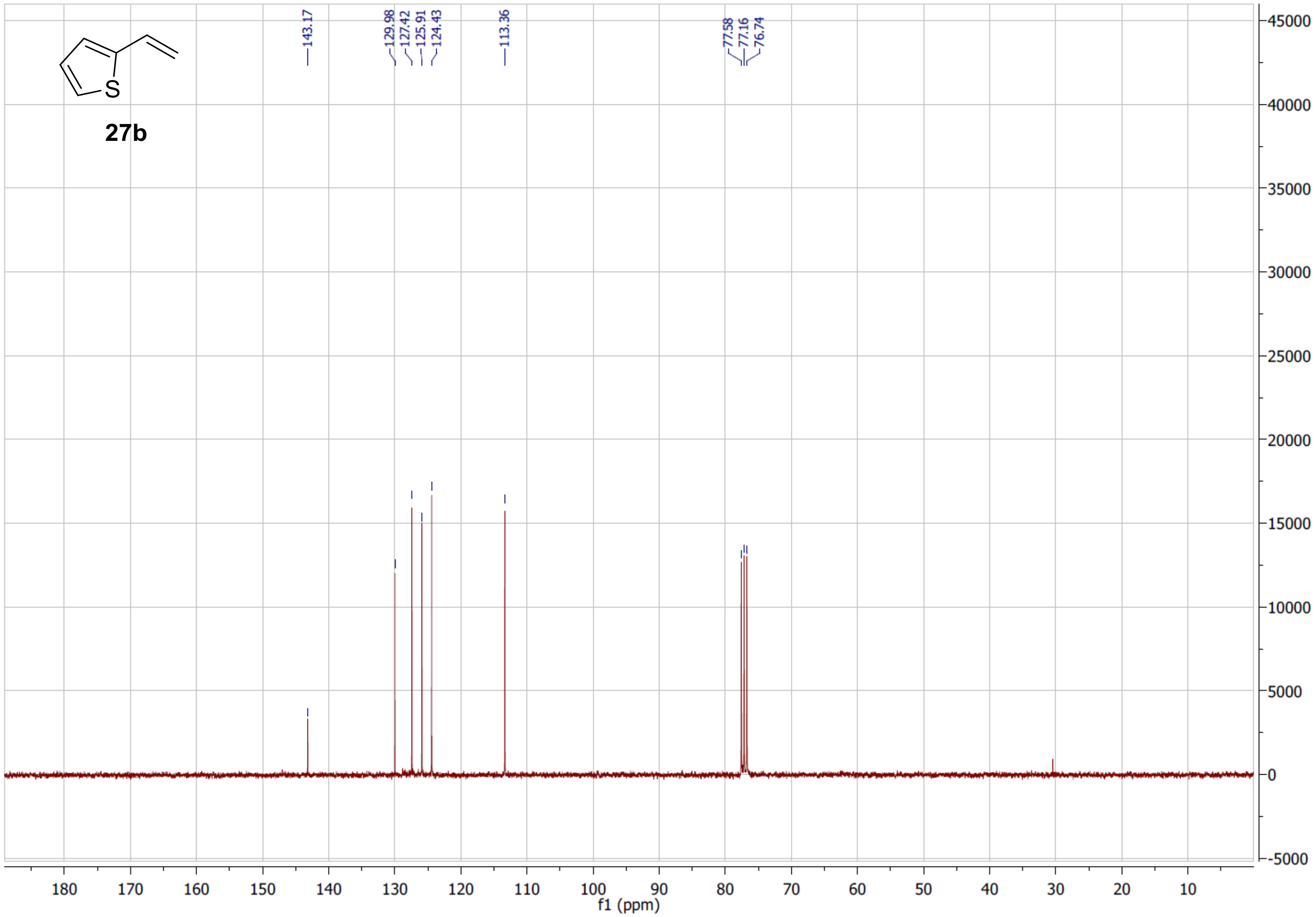

Figure S35. ^{13}C NMR spectrum of 27b (75 MHz, $CDCl_3$).

Section S8.6. 3,4-Dihydroxystyrene 18b[5]

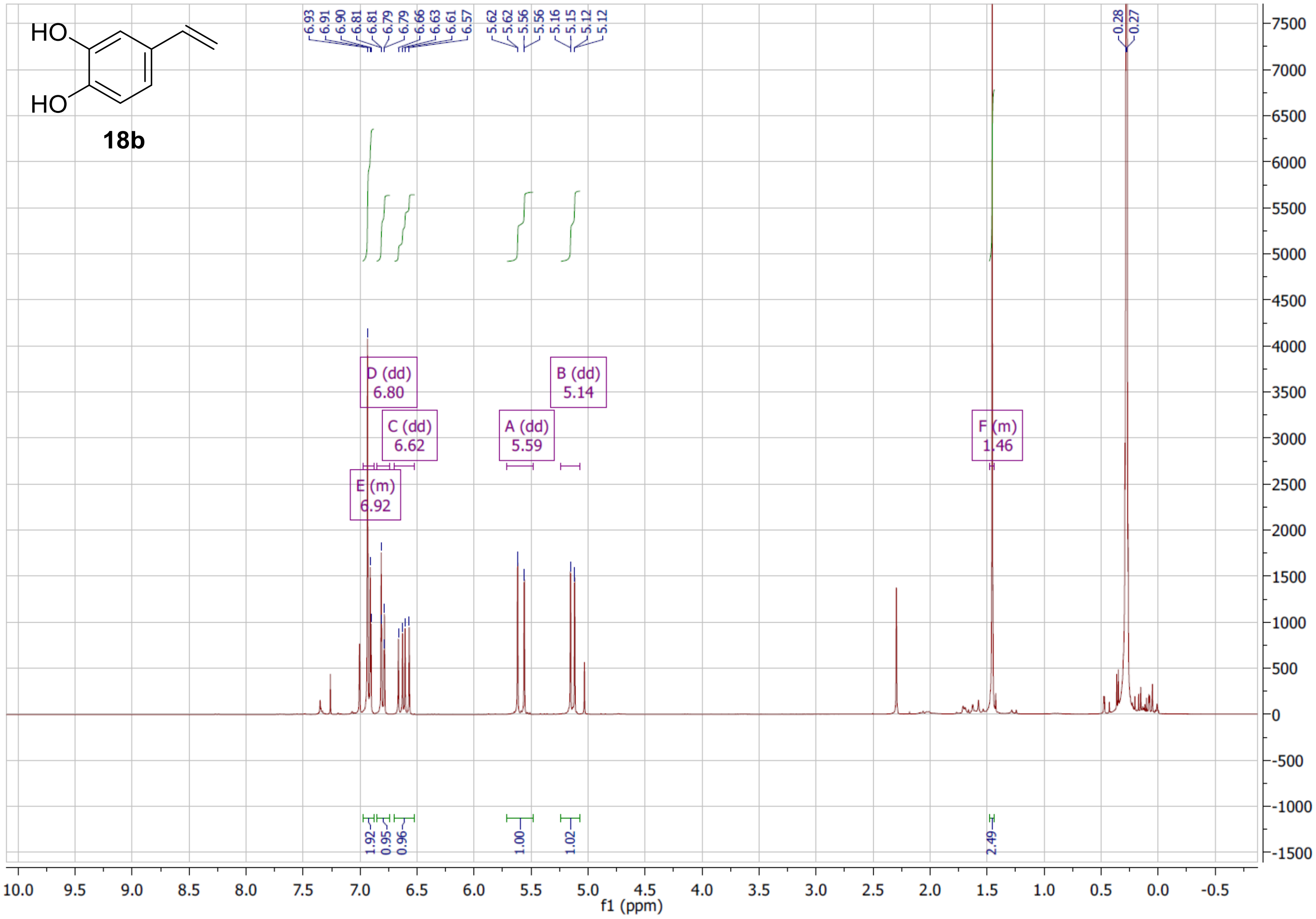

Figure S36. ^{1}H NMR spectrum of 18b (300 MHz, $CDCl_3$).

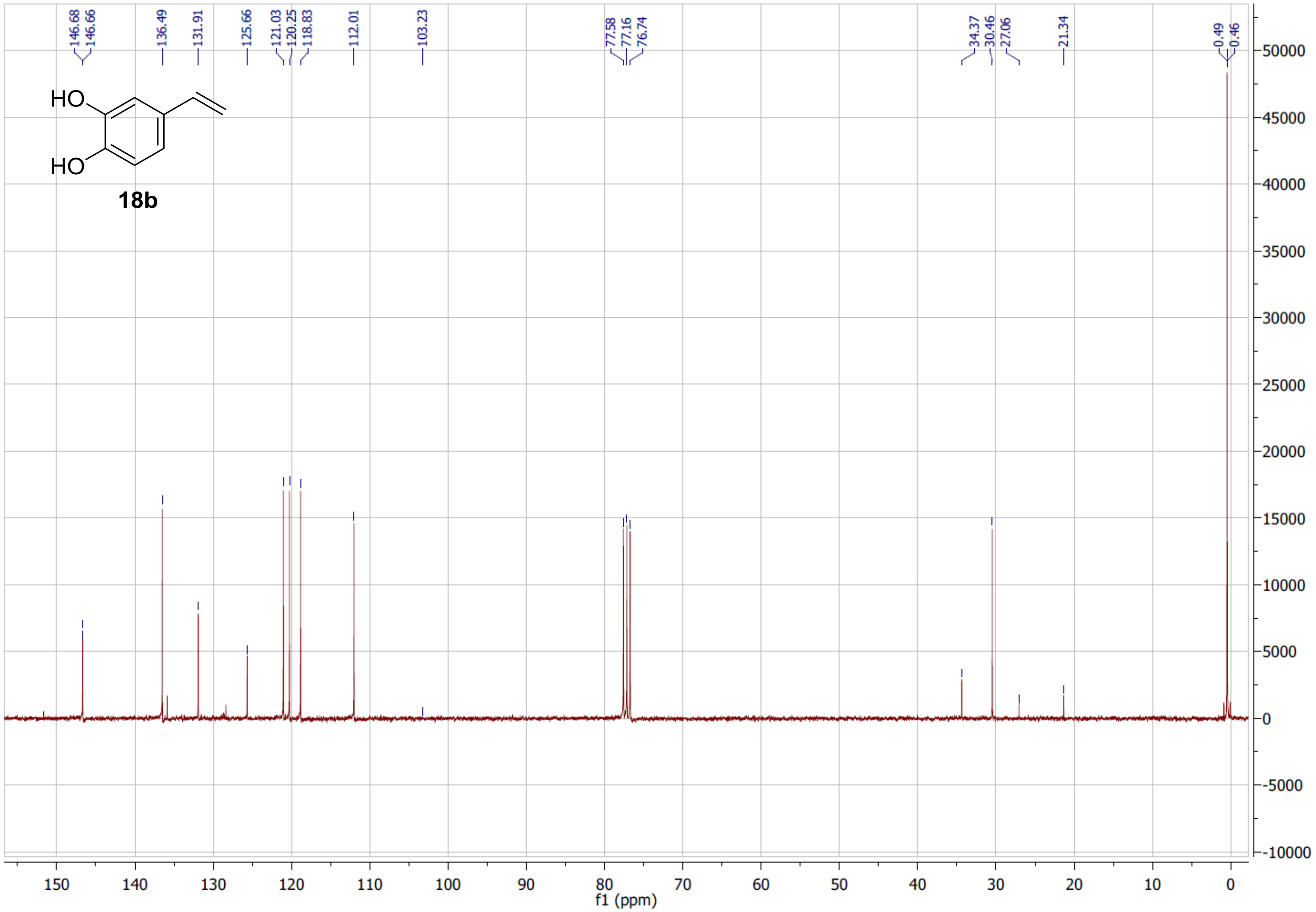

Figure S37. ^{13}C NMR spectrum of 18b (75 MHz, $CDCl_3$).

Section S8.7. 1,3-Pentadiene 36b[7]

a)

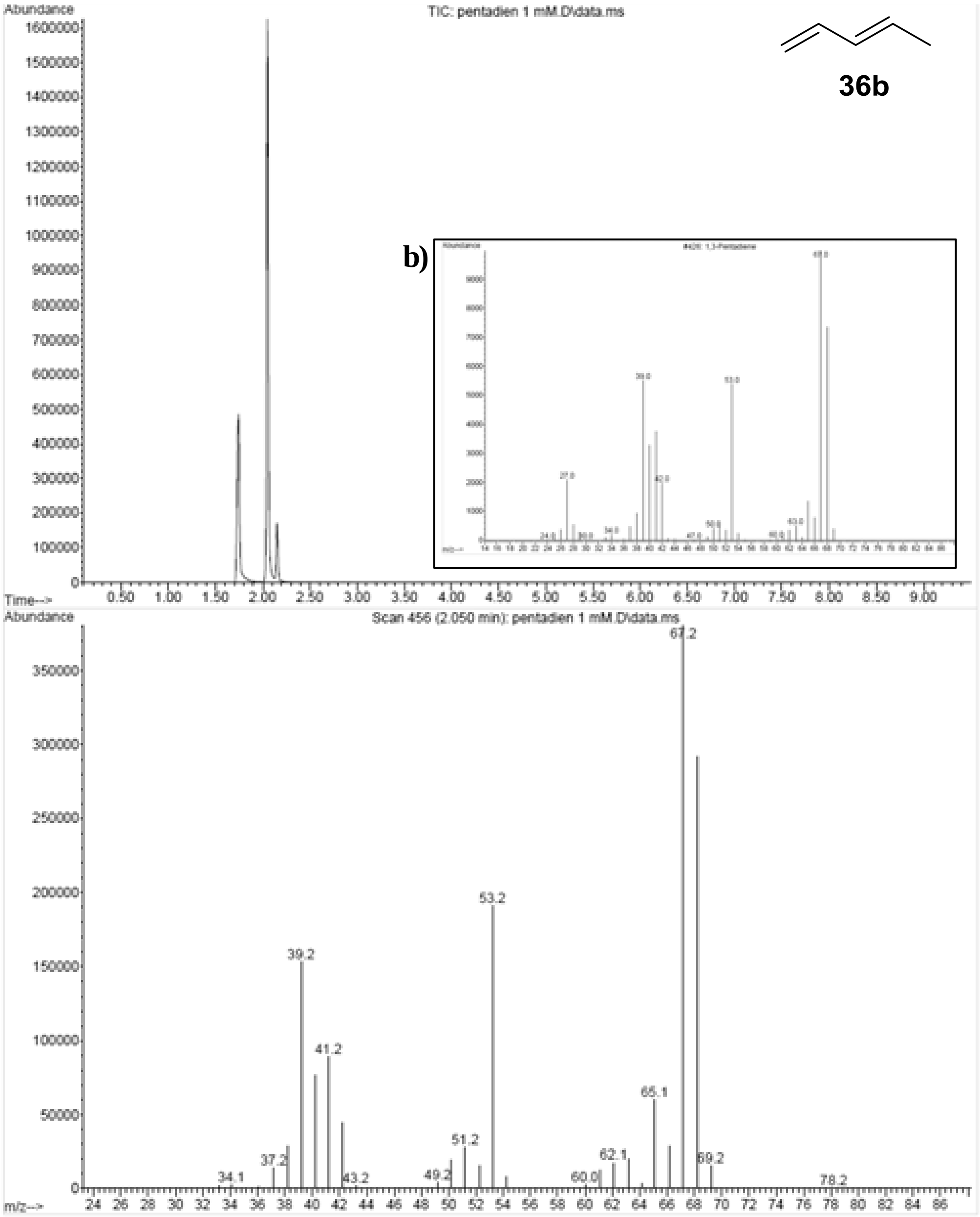

Figure S38. a) GC-MS spectra of crude 36b (68.06 g mol^{-1}) obtained by conversion of 36a with Fdc1; b) reference GC-MS spectra of 1,3-pentadiene (36b).

Section S8.8. Butadiene 37b[7a,8]

a)

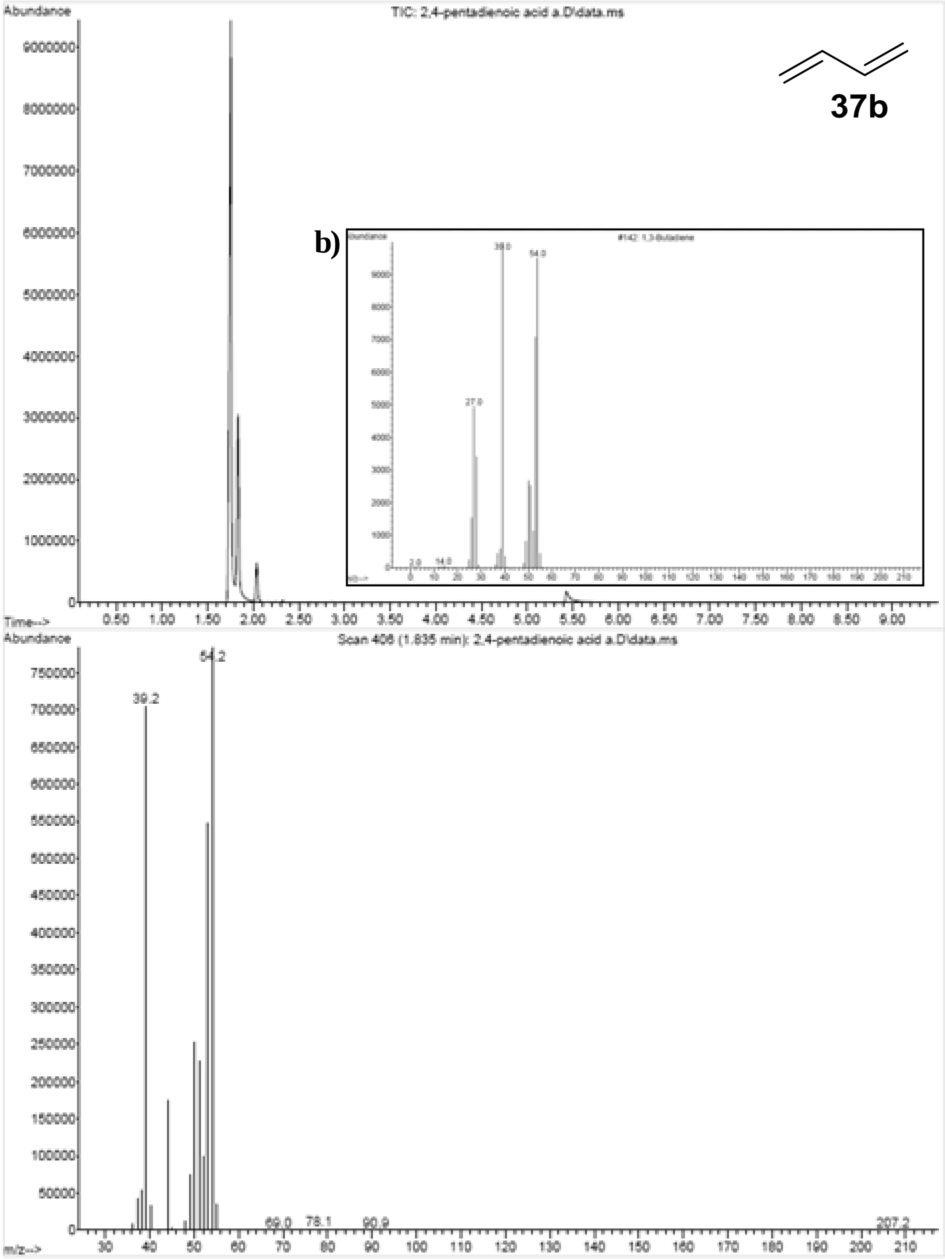

Figure S39. a) GC-MS spectra of crude 37b (54.05 g mol^{-1}) obtained by conversion of 37a with Fdc1; b) reference GC-MS spectra of butadiene (37b).

Section S9. References

(1) S. Cadot, N. Rameau, S. Mangematin, C. Pinel, L. Djakovitch, *Green Chem.* **2014**, *16*, 3089–3097.

(2) A. P. Kostikov, N. Malashikhina, V. V. Popik, *J. Org. Chem.* **2009**, *74*, 1802–1804.

(3) T. R. Valkute, E. K. Aratikatla, A. K. Bhattacharya, *Synth. Commun.* **2017**, *47*, 581–589.

(4) C.-X. Song, P. Chen, Y. Tang, *RSC Adv.* **2017**, *7*, 11233–11243.

(5) M. F. El-Behairy, E. Sundby, *Tetrahedron: Asymmetry* **2013**, *24*, 285–289.

(6) S. Baader, D. M. Ohlmann, L. J. Gooßen, *Chem. Eur. J.* **2013**, *19*, 9807–9810.

(7) a) M. Shiramizu, F. D. Toste, *Angew. Chem. Int. Ed.* **2012**, *51*, 8082–8086; b) E. M. Townsend, R. R. Schrock, A. H. Hoveyda, *J. Am. Chem. Soc.* **2012**, *134*, 11334–11337.

(8) B. C. Wiegand, M. E. Napier, C. M. Friend, P. Uvdal, *J. Am. Chem. Soc.* **1996**, *118*, 2962–2968.

S10. Full List of references from Manuscripts

[1] R. Tinikul, P. Chenprakhon, S. Maenpuen, P. Chaiyen, *Biotechnol. J.* **2018**, DOI 10.1002/biot.201700632.

[2] R. Kourist, J.-K. Guterl, K. Miyamoto, V. Sieber, *ChemCatChem* **2014**, *6*, 689–701.

[3] K. Fesko, M. Gruber-Khadjawi, *ChemCatChem* **2013**, *5*, 1248–1272.

[4] S. M. Glueck, S. Gümüs, W. M. F. Fabian, K. Faber, *Chem. Soc. Rev.* **2010**, *39*, 313–328.

[5] T. Ren, M. Patel, K. Blok, *Energy* **2006**, *31*, 425-451.

[6] M. Tuomaala, M. Hurme, A.-M. Leino, *Appl. Therm. Eng.* **2010**, *30*, 45–52.

[7] D.-H. Jung, W. Choi, K.-Y. Choi, E. Jung, H. Yun, R. J. Kazlauskas, B.-G. Kim, *Appl. Microbiol. Biotechnol.* **2013**, *97*, 1501–1511.

[8] G. A. Aleku, H. Man, S. P. France, F. Leipold, S. Hussain, L. Toca-Gonzalez, R. Marchington, S. Hart, J. P. Turkenburg, G. Grogan, N.J. Turner, *ACS Catal.* 2016, *6*, 3880–3889.

[9] G. A. Aleku, S. P. France, H. Man, J. Mangas-Sanchez, S. L. Montgomery, M. Sharma, F. Leipold, S. Hussain, G. Grogan, N. J. Turner, *Nat. Chem.* **2017**, *9*, 961–969.

[10] G. A. Aleku, J. Mangas-Sanchez, J. Citoler, S. P. France, S. L. Montgomery, R. S. Heath, M. P. Thompson, N. J. Turner, *ChemCatChem* **2018**, *10*, 515–519.

[11] S. P. France, G. A. Aleku, M. Sharma, J. Mangas-Sanchez, R. M. Howard, J. Steflik, R. Kumar, R. W. Adams, I. Slabu, R. Crook, G. Grogan, T. W. Wallace, N.J. Turner, *Angew. Chem. Int. Ed.* **2017**, *56*, 15589–15593.

[12] B. M. Nestl, S. C. Hammer, B. A. Nebel, B. Hauer, *Angew. Chem. Int. Ed.* **2014**, *53*, 3070–3095.

[13] A. J. L. Wood, N. J. Weise, J. D. Frampton, M. S. Dunstan, M. A. Hollas, S. R. Derrington, R. C. Lloyd, D. Quaglia, F. Parmeggiani, D. Leys, et al., *Angew. Chem. Int. Ed Engl.* 2017, *56*, 14498–14501.

[14] S. Noda, Y. Kawai, T. Tanaka, A. Kondo, *Bioresour. Technol.* **2015**, *180*, 59–65.

[15] M. Takemoto, K. Achiwa, *Tetrahedron Lett.* **1999**, *40*, 6595-6598.

[16] J. T. Claypool, D. R. Raman, L. R. Jarboe, D. R. Nielsen, *J. Ind. Microbiol. Biotechnol.* **2014**, *41*, 1211–1216.

[17] R. Fujiwara, S. Noda, T. Tanaka, A. Kondo, *J. Biosci. Bioeng.* **2016**, *122*, 730–735.

[18] R. McKenna, D. R. Nielsen, *Metab. Eng.* **2011**, *13*, 544–554.

[19] R. McKenna, B. Thompson, S. Pugh, D. R. Nielsen, *Microb. Cell Fact.* **2014**, *13*, 123.

[20] J. M. Salgado, R. Rodríguez-Solana, J. A. Curiel, B. de Las Rivas, R. Muñoz, J. M. Domínguez, *Enzyme Microb. Technol.* **2014**, *58–59*, 22–28.

[21] A. Dennig, M. Kuhn, S. Tassoti, A. Thiessenhusen, S. Gilch, T. Bülter, T. Haas, M. Hall, K. Faber, *Angew. Chem. Int. Ed.* **2015**, *54*, 8819–8822.

[22] M. A. Rude, T. S. Baron, S. Brubaker, M. Alibhai, S. B. Del Cardayre, A. Schirmer, *Appl. Environ. Microbiol.* **2011**, *77*, 1718–1727.

[23] J. L. Grant, C. H. Hsieh, T. M. Makris, *J. Am. Chem. Soc.* **2015**, *137*, 4940–4943.

[24] Z. Rui, X. Li, X. Zhu, J. Liu, B. Domigan, I. Barr, J. H. D. Cate, W. Zhang, *Proc. Natl. Acad. Sci. U. S. A.* **2014**, *111*, 18237–18242.

[25] N. A. Herman, W. Zhang, *Curr. Opin. Chem. Biol.* **2016**, *35*, 22–28.

[26] C. Wuensch, T. Pavkov-Keller, G. Steinkellner, J. Gross, M. Fuchs, A. Hromic, A. Lyskowski, K. Fauland, K. Gruber, S. M. Glueck, K. Faber, *Adv. Synth. Catal.* 2015, *357*, 1909–1918.

[27] M. Stratford, A. Plumridge, D. B. Archer, *Appl. Environ. Microbiol.* **2007**, *73*, 6534–6542.

[28] K. A. P. Payne, M. D. White, K. Fisher, B. Khara, S. S. Bailey, D. Parker, N. J. W. Rattray, D. K. Trivedi, R. Goodacre, R. Beveridge, P. Barran, Rigby S. E., N. S. Scrutton, S. Hay, D. Leys, *Nature* 2015, *522*, 497–501..

[29] S. A. Marshall, K. Fisher, A. N. Cheallaigh, M. D. White, K. A. P. Payne, D. A. Parker, S. E. J. Rigby, D. Leys, *J. Biol. Chem.* **2017**, *292*, 4623-4637.

[30] G. A. Aleku, B. Nowicka, N. J. Turner, *ChemCatChem* **2018**, *10*, 124–135.

[31] S. A. Marshall, K. A. P. Payne, D. Leys, *Arch. Biochem. Biophys.* **2017**, *632*, 209–221.

[32] M. D. White, K. A. P. Payne, K. Fisher, S. A. Marshall, D. Parker, N. J. W. Rattray, D. K. Trivedi, R. Goodacre, S. E. J. Rigby, N. S. Scrutton, S. Hay, D. Leys, *Nature* 2015, *522*, 502–506.

[33] K. L. Ferguson, N. Arunrattanamook, E. N. G. Marsh, *Biochemistry* **2016**, *55*, 2857–2863.

[34] F. Lin, K. L. Ferguson, D. R. Boyer, X. N. Lin, E. N. G. Marsh, *ACS Chem. Biol.* **2015**, *10*, 1137–1144.

[35] K. L. Ferguson, J. D. Eschweiler, B. T. Ruotolo, E. N. G. Marsh, *J. Am. Chem. Soc.* **2017**, *139*, 10972–10975.

[36] H. C. Kolb, M. G. Finn, K. B. Sharpless, *Angew. Chem. Int. Ed.* **2001**, *40*, 2004–2021.

[37] R. Huisgen, *Proc. Chem. Soc.* **1961**, *0*, 357–396**.**

[38] D. Leys, N. S. Scrutton, *Curr. Opin. Struct. Biol.* **2016**, *41*, 19–26.

[39] V. Piano, B. A. Palfey, A. Mattevi, *Trends Biochem. Sci.* **2017**, *42*, 457–469.

[40] C. T. Walsh, T. A. Wencewicz, *Nat. Prod. Rep.* **2013**, *30*, 175–200.

[41] M. Baunach, C. Hertweck, *Angew. Chem. Int. Ed.* **2015**, *54*, 12550–12552.

[42] K. Plasch, V. Resch, J. Hitœ, J. Popłoński, K. Faber, S. M. Glueck, *Adv. Synth. Catal.* **2017**, *359*, 959–965.

[43] C. Wuensch, N. Schmidt, J. Gross, B. Grischek, S. M. Glueck, K. Faber, *J. Biotechnol.* **2013**, *168*, 264–270.

[44] C. Wuensch, S. M. Glueck, J. Gross, D. Koszelewski, M. Schober, K. Faber, *Org. Lett.* **2012**, *14*, 1974–1977.

[45] S. E. Payer, S. A. Marshall, N. Bärland, X. Sheng, T. Reiter, A. Dordic, G. Steinkellner, C. Wuensch, S. Kaltwasser, K. Fisher, S. E. J. Rigby, P. Macheroux, J. Vonck, K. Gruber, K. Faber, F. Himo, D. Leys, T. Pavkov-Keller, S. M. Glueck, *Angew. Chem. Int. Ed.* 2017, *56*, 13893–13897.

[46] P. Pérez, L. R. Domingo, M. J. Aurell, R. Contreras, *Tetrahedron* **2003**, *59*, 3117–3125.

[47] A. L. Cardoso, T. M. V. D. Pinho e Melo, *Eur. J. Org. Chem.* **2012**, *2012*, 6479–6501.

[48] P. Dauban, G. Malik, *Angew. Chem. Int. Ed.* **2009**, *48*, 9026–9029.

[49] H. Pellissier, *Tetrahedron* **2007**, *16*, 3235–3285.

[40] F. Méndez, J. Tamariz, P. Geerlings, *J. Phys. Chem. A* **1998**, *102*, 6292–6296.

[51] D. H. Ess, G. O. Jones, K. N. Houk, *Adv. Synth. Catal.* **2006**, *348*, 2337–2361.

[52] K. N. Houk, *Acc. Chem. Res.* **1975**, *8*, 361–369.

[53] P. K. Chattaraj, *J. Phys. Chem. A* **2001**, *105*, 511–513.

[54] T. A. Rogers, A. S. Bommarius, *Chem. Eng. Sci.* **2010**, *65*, 2118–2124.

[55] H. Kohls, F. Steffen-Munsberg, M. Höhne, *Curr. Opin. Chem. Biol.* **2014**, *19*, 180–192.

[56] C. Wuensch, J. Gross, G. Steinkellner, A. Lyskowski, K. Gruber, S. M. Glueck, K. Faber, *RSC Adv.* **2014**, *4*, 9673–9679.

[57] Y. Ishii, Y. Narimatsu, Y. Iwasaki, N. Arai, K. Kino, K. Kirimura, *Biochem. Biophys. Res. Commun.* **2004**, *324*, 611–620.

[58] T. Matsui, T. Yoshida, T. Yoshimura, T. Nagasawa, *Appl. Microbiol. Biotechnol.* **2006**, *73*, 95–102.

[59] M. Wieser, N. Fujii, T. Yoshida, T. Nagasawa, *Eur. J. Biochem.* **1998**, *257*, 495–499.

[60] M. Wieser, T. Yoshida, T. Nagasawa, *J. Mol. Catal. B Enzym.* **2001**, *11*, 179–184.

[61] M. Wieser, T. Yoshida, T. Nagasawa, *Tetrahedron Lett.* **1998**, *39*, 4309–4310.

[62] T. Yoshida, K. Fujita, T. Nagasawa, *Biosci. Biotechnol. Biochem.* **2002**, *66*, 2388–2394.

[63] R. Bernini, E. Mincione, M. Barontini, G. Provenzano, L. Setti, *Tetrahedron* **2007**, *63*, 9663–9667.

[64] E. Zago, E. Dubreucq, J. Lecomte, P. Villeneuve, F. Fine, H. Fulcrand, C. Aouf, *New J. Chem.* **2016**, *40*, 7701–7710.

[65] P. Terpinc, T. Polak, N. Šegatin, A. Hanzlowsky, N. P. Ulrih, H. Abramovič, *Food Chem.* **2011**, *128*, 62–69.
